# Systematic Review of the Microbiological Performance of Household Water Treatment Technologies

**DOI:** 10.1021/acs.est.4c03494

**Published:** 2025-03-26

**Authors:** Gouthami Rao, Emma Wells, Catherine Reynolds, Rebecca Yoo, Erin Kowalsky, Jennifer DeFrance, Karl Linden, Joe Brown

**Affiliations:** † Department of Environmental Sciences and Engineering, 41474 University of North Carolina−Chapel Hill, Chapel Hill, North Carolina 27514, United States; ‡ Department of Civil, Environmental, and Architectural Engineering, 1877 University of Colorado Boulder, Boulder, Colorado 80303, United States; § Department of Civil and Environmental Engineering, 1372 Georgia Institute of Technology, Atlanta, Georgia 30332, United States; ∥ World Health Organization, Geneva 1211, Switzerland

**Keywords:** household water, water treatment, log-removal values, systematic review

## Abstract

Household water treatment (HWT) is a prevalent method for improving the safety of drinking-water. We conducted a systematic review of peer-reviewed literature from 1997 to 2021 on microbiological performance of common HWT technologies including porous ceramic filters, carbon block or membrane filtration, granular media filtration, thermal treatment, solar disinfection, UV irradiation, chemical disinfection, and combined coagulation-disinfectant products. We used the Preferred Reporting Items for Systematic Reviews and Meta-Analyses (PRISMA) best practices and searched SCOPUS, Web of Science, PubMed, and Agricola and further consulted an expert working group to add relevant publications reporting microbial performance of HWT (n = 396 peer-reviewed studies). Reported log_10_ reduction values (LRVs) varied widely across and within technology categories. We summarize microbial performance by technology type; pathogen class (bacteria, virus, protozoa); and study setting (field or laboratory). Combined coagulation-disinfectant products had the highest LRV performance for bacterial (6.3) and viral (5.3) classes, and porous ceramic had the highest LRV for protozoa (3) when *n* > 3. Findings can inform the selection of candidate HWT options, but factors such as product design, time burden of use, cost, and long-term maintenance requirements are important considerations in technological performance. Microbiological performance is meaningless if the technology is not used consistently, correctly, and sustainably.

## Graphical abstract

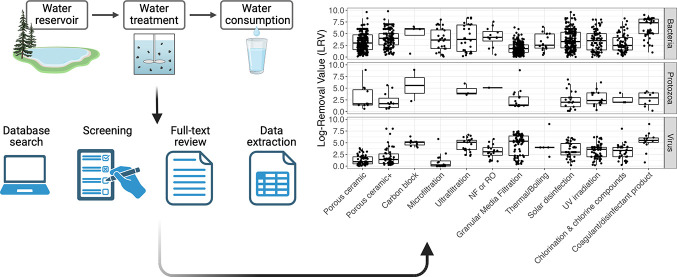

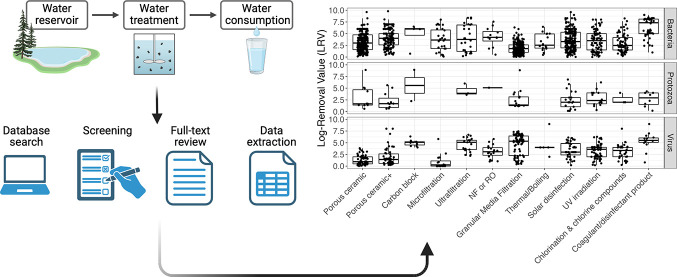

## Introduction

Approximately 2.2 billion people (approximately 27% of the global population) do not have access to safely managed drinking-water: water that is safe, available when needed, and accessible on premises.^1^ Safely managed drinking-water should be free from fecal contamination and its provision can save the lives of children, the immunocompromised, pregnant women, the elderly and even healthy adults from enteric or diarrheal diseases. Global progress in expanding safely managed drinking-water has not kept pace with needs.^2,3^

When and where drinking-water may be unsafe,^4^ the World Health Organization (WHO) recommends the interim solution of treating water before consumption at the point-of-use (POU) or point-of-entry (POE) for a household, a strategy often referred to as household water treatment (HWT).^5^ HWT treatment is necessary when and where centralized treatment is unavailable or inadequate, where supplies are intermittent,^6^ or where household water storage is required. When water must be fetched outside the home–a reality for millions of people, primarily women and children in low- and middle-income countries (LMICs) – microbial water quality diminishes during storage and handling.^1,7–12^ For these reasons and others, HWT remains a common global practice^13,14^ and has been shown to substantially reduce diarrheal diseases among users,^15^ despite concerns about the sustainability^16^ and scalability^17^ of this means of safe water provision.

A wide range of HWT technologies have been developed to remove or inactivate microbial contaminants. These include porous ceramic, carbon block, or membrane filtration;^13^ granular media filtration;^18^ heat inactivation;^19^ UV irradiation;^20^ solar disinfection;^21^ chemical disinfection;^22^ or multiple barrier treatments.^23^ The performance of technologies in reducing microbial contaminants and improving water safety has been reported in a large and rapidly expanding literature. Studies include controlled laboratory (i.e., efficacy) trials, trials under real-world or field conditions (i.e., effectiveness trials), and in epidemiology or risk assessment studies. There are also a number of systematic testing programs for evaluating performance using comparable methods, including the World Health Organization’s International Scheme to Evaluate Household Water Treatment Technologies (WHO Scheme), which serve an important function in evaluating efficacy of specific technologies under controlled conditions.^24,25^ Although treatment efficacy is not the sole criterion that should be used in evaluating the suitability of specific HWT approaches for a given application, adherence or compliance, cost, appropriateness for the context, and other factors can be equally important^26^—HWT technologies can only improve the microbiological safety of drinking-water if they achieve substantial reduction of pathogens. For HWT, WHO has established tiered health-based targets of no greater than 1 × 10^–6^ and 1 × 10^–4^ disability-adjusted life years per person per year attributable to drinking-water. The log_10_ reduction values (LRVs) required to meet these targets typically are at least 4 log_10_ reduction (99.99%) in the top tier, and at least 2 log_10_ reduction in the lower tier.

Practitioners and researchers use reported HWT technology microbial reductions to inform selection of drinking-water treatment options in underserved settings, where their role in improving water safety may be most important. Since 2008, the World Health Organization’s Guidelines for Drinking-water Quality (GDWQ) have included a summary of expected HWT LRVs across categories of common technologies and pathogen class, with further notes on specific factors that may influence performance within these particular ranges. This information has been retained in more recent versions of the GDWQ including the latest edition, published in March 2022.^5,27^ As interest in HWT has grown and the number of HWT performance studies in the peer-reviewed literature has expanded, there is a need for a systematic assessment of HWT performance to inform the update of the GDWQ and better guide the HWT practice community. Our objective in this study was to conduct a systematic, replicable review of the peer-reviewed literature to summarize reported microbial LRVs across common HWT technologies. Although treatment performance is known to vary widely across study settings, technologies, and by method of evaluation, we hypothesized that a systematic review of the evidence would yield generalizable and useful insight of widespread interest in the water and health community of researchers, practitioners, and policymakers.

## Materials and Methods

### Search Strategy, Eligibility Criteria, and Data Extraction

We followed Preferred Reporting Items for Systematic Reviews and Meta-Analyses (PRISMA) best practices for the review and included the PRISMA checklist in Table S2.^28^ We conducted literature searches from January 1, 1997 to March 23, 2021 using a standardized search string query for the identified target databases, PubMed, Agricola, SCOPUS, and Web of Science (Supporting Information Text S1). Additionally, we consulted subject-matter experts (SMEs) by e-mail and in a series of workshops to identify missing literature regardless of publication year. SME workshops further defined technology types to include in the review, reviewed appropriateness of binning technologies by type, and added important context on the interpretation of individual studies. Lastly, we included WHO evidence on microbial performance of HWT from a key document supporting data in the current GDWQ Table 7.8 and the WHO Scheme Rounds I and II.^29–31^ Eligible studies included in the final systematic review consisted of *a priori* categories of common HWT with reported quantitative microbial reduction estimates, typically microbial count data, in pre- and post-treatment water. We excluded studies that reviewed existing literature. We further excluded combination or multibarrier treatment technologies (aside from coagulation and disinfection), as the wide variability of these technologies precludes binning them by category to produce summary statistics. We did not consider treatment performance with respect to chemical or radiological contaminants. We excluded bench-scale studies of novel methods that are not currently used in practice and methods that have been excluded from WHO recommendations. These included standalone silver or silver compounds for toxicity and efficacy concerns, outside of *Legionella* control applications;^32^ iodine because of toxicity concerns in long-term use;^33^ and simple sedimentation as a standalone treatment. We considered peer-reviewed publications with no language restrictions.

### Selection Process, Data Collection Process, and Data Items

After the electronic database search, we imported all references into a reference management software (Zotero version 6.0) for initial title and abstract review to remove duplicates and exclude out of scope studies. We reviewed full manuscripts to assess the availability of data meeting inclusion criteria. Two reviewers independently screened all studies to determine review inclusion. Any questionable studies were screened through one senior reviewer (JB) to come to a consensus.

We extracted extensive data from papers reflecting key methodological details; the full database is publicly available at https://osf.io/jdeyx/. Variables included the scale of studies as lab (bench-scale), pilot (in between lab and field), or field-based (full implementation of intervention). Relevant entries were determined as “efficacy” or “effectiveness” studies. Efficacy was defined as studies performed under ideal conditions with trained professionals in which intervention protocols are strictly enforced and standardized. Effectiveness was defined as studies in real-world conditions in which interventions are implemented in the field and intervention protocols may vary on a user-basis. The origin of microbes in pretreatment water was defined as naturally occurring or spiked-in experimentally. The challenge water or pretreatment water was defined as drinking/potable water, from an environmental source (lake, pond, river, etc.), or artificially spiked deionized water from lab. We also documented whether or not water quality data were included. We recorded pathogen type (bacteria, virus, protozoa), including genera and species for each pathogen tested, along with details of measurement (e.g., molecular or culture). We noted the technology type, binned into *a priori* categories. We recorded arithmetic mean pre- and post-treatment counts, standardized per specified volume or percent microbial reduction. Microbial count and LRV estimates existed in paper text, tables, and graphs; we included any of these provided the data were interpretable as presented. If standard deviation, standard errors, confidence intervals, or other distributional statistics were reported in studies assessed, then we included those data. We noted whether positive and negative controls were reported, the time and volume over LRV demonstrated, and if LRV estimates were constrained by detection limits. For example, in effectiveness studies where microbes are typically not spiked in experimentally, the highest possible LRV is constrained by pretreatment microbial density. In such cases, reported LRVs may underestimate potential microbial performance.

There has been some debate on the most appropriate method of presenting LRVs. While some argue for the use of effective LRVs or a weighted-averaged LRV,^34^ others indicate arithmetic mean LRVs are still of valuable importance when uncertainty estimates or other statistical distribution values are generated to give context to point estimates.^35^ Here, we report either arithmetic mean LRVs or calculated arithmetic mean LRVs and distributional statistics including the 95% confidence interval and interquartile range. We further supply the full database in case other specific estimates of central tendency or distributional statistics would be of value (https://osf.io/jdeyx/).

### Effect Measures and Synthesis Methods

We report effect measures for each treatment technology as a reported LRV or a calculated LRV using eq 1 if pre- and post-treatment microbial counts were provided using arithmetic means. If a percent reduction of microbial concentrations was given, we calculated the pre- and post-treatment values using eq 2 and then used eq 1 to calculate the LRV:

Calculated LRV using pre- and post-treatment concentrations.

1 $$\mathrm{LRV}=\log_{10}\frac{MeanPre‐treatmentmicrobialconcentration}{MeanPost‐treatmentmicrobialconcentration}$$

Calculated post-treatment using percent reductions.

2 $$Meanpre‐treatmentconcentration\times \left(1-\left(\frac{\%Reduction}{100}\right)\right)=Meanpost‐treatmentconcentration$$

We assumed nondetects in post-treatment water to be at a detection limit of 1 for the purposes of calculation, representing a conservative estimate of the true LRV. We included LRV calculations for nondetect estimates with other studies that had preand post-treatment microbial concentrations. We binned studies into treatment groups and first assessed LRVs between microbial classes. We further disaggregated data by study type (efficacy vs effectiveness). We extracted all relevant data into Microsoft Excel with statistical analyses in R version 3.6.3 for arithmetic mean LRVs, 95% confidence intervals (CI), interquartile ranges (IQR), and data quality control such as standardizing variable names and formats. We included 95% CIs as a range of values that likely includes the population mean value with a 95% degree of confidence.^36^

## Results and Discussion

The total number of studies screened through the electronic databases was 15,139, with an additional n = 129 from SMEs, 39 from WHO Scheme sources, and 55 references from the Managing Water in the Home key document.^25,30,31^ From the total screened, 14,272 were considered irrelevant and removed. Of the remaining 1090 relevant records, we removed 205 duplicates. We assessed full-text articles for eligibility (n = 885) and included 396 studies in the final analysis, meeting all inclusion criteria (Figure 1). The full data set is available at https://osf.io/jdeyx/.

**Figure 1:**
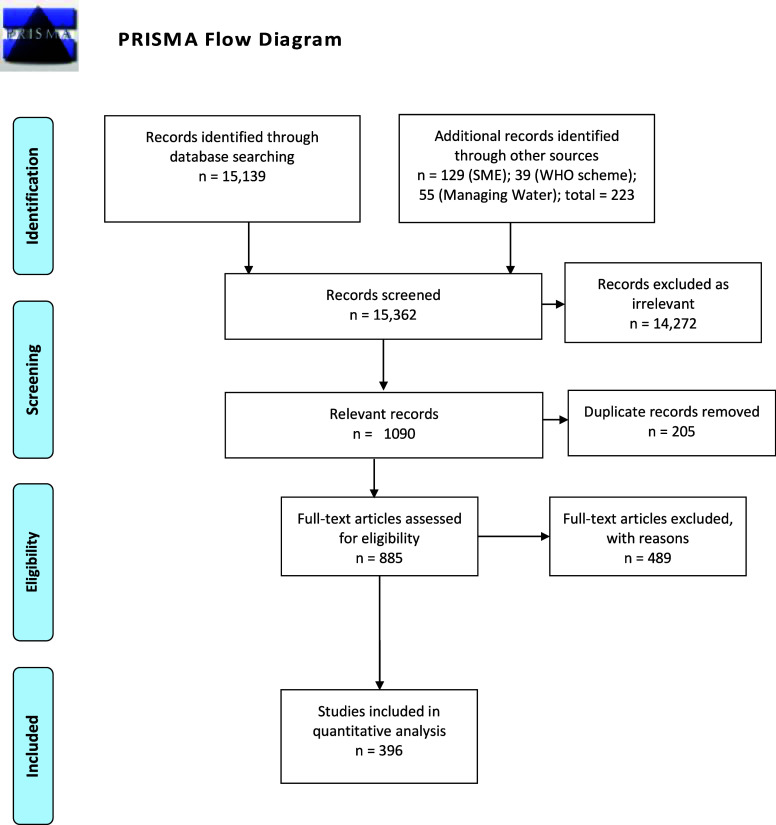
PRISMA flow diagram depicting the identification, screening, eligibility, and included study numbers for household water treatment technologies.

A comprehensive microbial LRV by pathogen type, efficacy vs effectiveness study, household water treatment technology, number of journal articles and total data points for each category is summarized in Table 1 and LRVs compared from this search to the WHO GDWQ are found in Supporting Information Table S3. We also include a Supporting Information Table S4 of LRV interquartile ranges (25th and 75th percentiles) for each household water treatment technology as another metric of how the data was distributed. Generally, performance testing under laboratory conditions yielded higher LRVs than studies conducted under field or typical operating conditions. The category with the highest number of papers included was solar treatment (n = 86) and the lowest was carbon block (n = 4). Further, for protozoa, there were inadequate data (one to two data points) to calculate mean LRVs for carbon block and NF/RO while there was no data for microfiltration or boiling. The full reference citations are included as Supporting Information Text S2. Each household water treatment category’s LRV results was considered in alignment with the WHO GDWQ’s recommended LRVs.

**Table 1:** Log-Removal Value Table for Pathogen Types across Household Water Treatment Technologies^d^

**Treatment process**	**Pathogen** **group** ^a^	**Total number of studies**	**Total number of point estimates**	**Mean** **log_10_** **reduction**value: all studies (95% CI)	**n**	**Mean** **log**_**10**_ **reduction**value: controlled laboratory conditions (95% CI)	**n**	**Mean** **log**_**10**_ **reduction**value: typical operational conditions (95% CI)
**Porous ceramic, carbon block or membrane filtration**								
Porous ceramic	Bacteria	75	226	3.2 (3.0–3.5)	182	3.5 (3.2–3.8)	44	2.1 (1.6–2.7)
	Viruses		56	1.3 (1.0–1.6)	54	1.3 (1.0–1.6)	2	1.6
	Protozoa		11	3.0 (1.3–4.7)	8	3.1 (0.79–5.5)	3	2.6 (−2.6–7.8)
Porous ceramic, including modification	Bacteria	49	160	4.0 (3.7–4.3)	132	4.4 (4.1–4.7)	28	2.2 (1.5–2.9)
	Viruses		52	1.9 (1.4–2.5)	51	2.0 (1.5–2.5)	1	1.22
	Protozoa		12	2.5 (1.3–3.7)	12	2.5 (1.3–3.7)	0	^b^
Carbon block	Bacteria	4	4	4.8 (0.46–9.1)	3	6.2 (5.4–6.9)	1	0.72
	Viruses		11	5.0 (4.5–5.4)	11	5.0 (4.5–5.4)	0	^b^
	Protozoa		2	5.6	2	5.6	0	^b^
Membrane, microfiltration	Bacteria	14	28	3.6 (2.7–4.6)	25	3.8 (2.8–4.8)	3	2.0 (−1.9–5.8)
	Viruses		17	0.85 (0.07–1.6)	17	0.85 (0.07–1.6)	0	^b^
	Protozoa		0	^b^	0	^b^	0	^b^
Membrane, ultrafiltration	Bacteria	21	26	4.0 (2.9–5.2)	12	5.6 (4.1–7.1)	14	2.7 (1.2–4.2)
	Viruses		21	4.6 (4.0–5.3)	20	4.7 (4.0–5.4)	1	3.0
	Protozoa		3	4.5 (1.3–7.7)	1	3.6	2	5.0
Membrane, NF or RO	Bacteria	10	12	4.2 (2.9–5.6)	8	5.1 (3.6–6.5)	4	2.6 (−0.08–5.3)
	Viruses		19	3.1 (2.4–3.8)	15	3.0 (2.2–3.9)	4	3.4 (2.2–4.6)
	Protozoa		1	5.1	1	5.1	0	^b^
**Granular media filtration**								
Granular Media Filtration (Biosand (BSF) + slow sand filtration)	Bacteria	73	220	1.9 (1.7–2.0)	156	2.0 (1.8–2.2)	64	1.6 (1.4–1.9)
	Viruses		77	4.5 (4.0–5.0)	72	4.7 (4.2–5.2)	5	1.7 (1.1–2.3)
	Protozoa		17	2.5 (1.5–3.5)	8	2.5 (0.30–4.7)	9	2.5 (1.5–3.5)
**Thermal (heat) treatment**								
Boiling	Bacteria	17	19	3.0 (2.1–3.9)	4	5.3 (3.8–6.8)	15	2.4 (1.6–3.3)
	Viruses		7	4.4 (2.4–6.4)	7	4.4 (2.4–6.4)	0	^b^
	Protozoa		0	^b^	0	^b^	0	^b^
**Solar disinfection**								
Solar Treatment (solar UV radiation + thermal, solar photocatalysis)	Bacteria	86	209	3.6 (3.3–3.9)	88	3.9 (3.5–4.4)	121	3.4 (3.0–3.7)
	Viruses		41	3.5 (3.0–4.0)	23	4.5 (4.0–4.9)	18	2.2 (1.6–2.8)
	Protozoa		21	2.4 (1.6–3.3)	15	3.0 (1.9–4.0)	6	1.0 (−0.06–2.2)
**UV Irradiation**								
UV irradiation (UV lamps + UV LED)	Bacteria	64	89	3.5 (3.0–3.9)	83	3.4 (2.9–3.8)	7	4.5 (2.4–6.7)
	Viruses		74	3.4 (3.0–3.8)	74	3.4 (3.0–3.8)	0	^b^
	Protozoa		14	2.8 (1.9–3.8)	14	2.8 (1.9–3.8)	0	^b^
**Chemical disinfection**								
Chlorination and chlorine compounds^c^	Bacteria	39	77	3.1 (2.6–3.5)	55	3.3 (2.8–3.9)	22	2.5 (1.8–3.2)
	Viruses		36	3.4 (2.7–4.1)	34	3.5 (2.7–4.2)	2	2.8
	Protozoa (non-*Cryptosporidium*)		4	2.0 (−0.69–4.6)	2	1.0	2	3.0
**Combination (multiple-barrier) treatment**								
Coagulation/disinfection (e.g., commercial powder sachets or tablets)	Bacteria	18	51	6.3 (5.8–6.9)	46	6.5 (6.0–7.1)	5	4.3 (0.93–7.7)
	Viruses		17	5.3 (4.4–6.2)	16	5.3 (4.4–6.3)	1	4.6
	Protozoa		8	2.6 (1.3–3.9)	7	2.4 (1.0–3.9)	1	4.0

a NB: only technologies supported by the minimum number of technology–pathogen class pairs required for computation of a mean LRV (n = 3 studies) are included in this table. NB: a significant number of POU technologies use multiple mechanisms; such combined technologies are not included here, because there are too many to list individually and collapsing them into a single category would not be useful due to the differences between technologies. Many combination technologies have been tested and performed in the WHO Household Water treatment scheme and where applicable on specific performance and technologies can be accessed at the scheme (link below). NB: Since performance also depends on specifics of the method/device it is preferable to evaluate the performance of individual products. Products specifically tested under the WHO Household Water Treatment Scheme can be found at https://www.who.int/tools/international-scheme-to-evaluate-household-water-treatment-technologies/products-evaluated

b Includes data from surrogates for that pathogen class.

c Fewer than 3 estimates; insufficient data to compute 95% CI. If < 3 estimates, only the mean LRV is shown.

d Including chlorine-based disinfection treatment such as sodium dichloroisocyanurate (NaDCC), sodium hypochlorite, calcium hypochlorite, *N*-halamine polymer disinfectant beads, chlorine dioxide and chlorinated resins.

### Porous Ceramic, Carbon Block, or Membrane Filtration

#### Porous Ceramic

Ceramic filters use porous fired clay to capture microbes. In underserved settings, these filters are often made in a pot design.^13^ Ceramic filters have been amended using various materials such as metal oxides such zinc,^37^ iron^38,39^ or diatomaceous earth.^40,41^ Some studies considered chemical augmentation, most commonly applied in the form of silver or copper salts,^42^ producing performance data from porous ceramic filters with and without additives.^43–46^ These additives have been used as bacteriostatic agents or to improve performance of the filters.^47–49^ As shown in Table 1, for ceramic filters supplemented with metal oxides or silver nanoparticles, we canculate an overall mean LRV of 4.0 for bacteria (95% CI: 3.7–4.3), followed by protozoa at 2.5 (1.3–3.7), and viral reductions at 1.9 (1.4–2.5). All of these microbial classes had higher LRVs compared to only porous ceramic without additives, except for protozoa at 3.0 (1.3–4.7). However, the findings comparing filters with and without additives should be interpreted with caution because of the inconsistent results between pathogen classes, wide heterogeneity of amendment types (including sizes and shapes of nanoparticles and capping agents used in silver nanoparticle synthesis) encompassing the additive group and also there are questions on whether or not the additives leach over time. Further research is therefore warranted.

#### Carbon Block

Carbon block treatment technology typically uses activated carbon to retain microbial organisms or particles by size (∼0.1 μM).^50^ Water may flow slower through this filtration step while the electrostatic attraction of the negatively charged microbe attaches to the positively charged carbon surface. For carbon block alone, an LRV upward of 5.0 (4.5–5.4) was calculated for viruses and 6.2 (5.4–6.9) for bacteria across efficacy studies. The few data points (n = 3) for bacteria efficacy studies suggest the findings should be interpreted with caution.

#### Membrane–microfiltration, ultrafiltration, nanofiltration or reverse osmosis

Membrane filtration relies on size exclusion as the primary treatment mechanism. Water is forced through a membrane, or gravity driven, and contaminants larger than the effective pore size are excluded. By convention, we defined pore size categories as microfiltration (∼0.1 μm), ultrafiltration (∼0.01 μm), nanofiltration (∼0.001 μm), and reverse osmosis (∼0.0001 μm)^51^ and we used authors’ own definitions when categorizing filtration technologies. HWT technologies that use membrane filtration often have higher LRV when tested in the laboratory as compared to LRV results from in-field testing.^52^ This is also seen in the summary data in Table 1 where bacterial and viral classes consistently performed better in the laboratory controlled environment compared to field environments across all membrane filtration categories. Unexpectedly, bacterial LRVs from efficacy studies were similar between ultrafiltration and nanofiltration/RO, with average LRVs calculated at 5.6 (4.1–7.1) and 5.1 (3.3–6.9), respectively, whereas for viruses overall lower LRVs were calculated across efficacy studies, at 4.7 (4.0–5.4) for ultrafiltration and 3.0 (2.2–3.9) for nanofiltration/RO. Microfiltration data were scarce across field-based studies for all pathogen classes.

### Granular Media Filtration (GMF)

At a household level, most technologies considered as granular media filtration include the biosand filter (BSF), which uses intermittently operated slow sand filtration–and the associated biological processes–to reduce microbial contaminants in drinking-water. Slow sand filters have a biologically active layer called the *schmutzdecke*, which occurs at the interface between sand and water usually on the surface and harbors dense and diverse microbial populations. This layer then retains incoming microbes and leads to their inactivation and biodegration. The BSF can vary in design and be intermittently operated with varying flow rates.^53,54^ Advantages of using a BSF is it can be used without electricity, is relatively inexpensive, and may be easy to use.^55^ Unexpectedly, GMF technology overall performed better for viral microbes (LRV = 4.5 [4.0–5.0]) over bacterial and protozoan classes (LRV = 1.9 [1.7–2.0] and 2.5 [1.5–3.5], respectively).

### Thermal (Heat) Inactivation

#### Boiling

Thermal, or heat-based, inactivation through boiling water or heating with fuel is one of the oldest, most accessible, and consistently effective water treatment technologies.^29^ Another advantage to boiling water is that most households already have the materials (i.e., fuel and a container), whereas other treatment options may require additional capital to maintain intervention use.^56^ An estimated 1.2 billion people have been estimated to practice boiling.^14^ Heating to pasteurization temperatures (e.g., > 63 °C for 30 min for milk) can also be effective, but may be more difficult to achieve in HWT applications because water temperature is not usually recorded with a thermometer in practice. The use of solar irradiation or a different combination of UV irradiation from sunlight and heat are in separate category for the purposes of this review (solar disinfection).

The WHO recommends that water temperature is raised so that a rolling boil is achieved, followed by removing the water from heat and allowing it to cool, then protecting the water from post-treatment contamination, i.e. safe water storage.^57^ Field trials have shown that recontamination of treated water often occurs through improper storage in wide-mouth or open containers^7,14,19,29,56,58^ or through contaminated hand contact during access.^59^ Indeed, several boiling studies included in this analysis represent boiling practices and water storage in suboptimal conditions. The boiling data in Table 1 indicate an LRV of 3.0 (2.1–3.9) for bacteria and 4.4 (2.4–6.4) for viruses, with no information available for protozoa.

### Solar Disinfection

#### Solar Treatment (Solar UV Radiation + Thermal, Solar Photocatalysis)

Solar irradiation is another water treatment technology to disinfect water. A general advantage to using solar treatment is the ability to use sunlight as a UV source. Most common is the solar water disinfection or SODIS system that uses clear, plastic containers (typically 2-L polyethylene terephthalate (PET)) and allows for penetration by UV radiation from sunlight up to 48 h.^60^ This technology mechanisms combines reaction of UV radiation, oxidative activity with dissolved oxygen and heat. Other types of solar disinfection systems include different containers such as UV-penetrable plastic bags and panels.^61^ Others use solar irradiation in dark or opaque containers and rely on heat from the sunlight energy to be primary driver to inactivate microbes. Additionally, solar photocatalysis uses a semiconductor as a catalyst for the chemical reaction that occurs between sunlight and microbial inactivation.^62–64^ Using a semiconductor catalyst can greatly reduce the microbial inactivation time.^65^ Additional studies also utilize compound parabolic concentrators (CPCs) to decrease the SODIS processing time.^66–68^ Our results indicate the bacterial category housed the greatest number of data points (n = 209) and had the highest mean LRV of 3.6 (3.3–3.9) for field and lab studies, with the protozoan class being approximately 1 log_10_ lower with an LRV of 2.4 (1.6–3.3).

### UV Irradiation

#### UV Irradiation (UV Lamps + UV LED)

Drinking-water treatment technologies can also use UV light irradiation to inactivate microbes, either from UV lamps or UV light-emitting diodes (LEDs). Most UV lamps use low-pressure mercury arc lamps to produce monochromatic UV radiation at a germicidal wavelength of 254 nm. These technologies expose water in a vessel or flow-through reactors to UV radiation at a sufficient dose or fluence to inactivate waterborne pathogens.^21,69,70^ Limitations on this technology mainly concern reliable electricity, cost and maintenance requirements making it challenging to implement in underserved settings. Newer germicidal UV-LED devices (wavelength between 255 and 285 nm) can be manufactured in small sizes, can be designed to have efficient on/off cycling conditions, emit little heat, and can be used with simple batteries and may be solar-powered or use mechanical energy from a hand crank.^20,71,72^ Advantages of this technology include ease of use and long battery life, which may appeal to low- and middle-income countries lacking consistently available electricity. The UV irradiation category characterized by the highest LRV was for bacteria tested in effectiveness studies with an LRV of 4.5, but with a mean LRV of 3.5 when considering both efficacy and effectiveness studies. Protozoan and viral classes had lower LRVs of 2.8 and 3.4, respectively and were only identified in efficacy studies.

### Chemical Disinfection

#### Chlorination and Chlorine Compounds

Chlorination is one of the most abundantly used HWT methods, largely due to its widespread availability and relatively low cost.^13^ Chlorine treatment can take many forms, including the use of solid tablets (sodium dichloroisocyanurate), liquid (sodium hypochlorite), or powders (calcium hypochlorite) with specific dosage recommendations to meet the WHO recommendation of a 0.2 mg/L concentration of free residual chlorine at the point-of-use.^73^ Free chlorine is an excellent oxidant that can readily inactivate a wide range of bacteria and viruses, with notably reduced efficacy for protozoa generally and *Cryptosporidium* specifically.^74^ Its use allows for maintenance of a chlorine residual, potentially protecting water from recontamination for a limited time. Chlorine demand limits the efficacy of the method, and typically it is most suited to water that is low in turbidity.^74^ Additionally, chlorine effectiveness is affected by pH and temperature conditions. However, even with appropriate chlorine dosing, improper drinking-water storage and handling can diminish the protective effect of chlorine.^22,75,76^ Chlorination has been widely and successfully used in outbreak and emergency settings, and has been especially critical in cholera response.^74,77–79^ In this HWT category, we observed the greatest average LRV of 3.4 for viruses, followed closely by the bacteria with an average LRV of 3.1. Neither viral and protozoan classes had sufficient data to calculate a 95% confidence interval for effectiveness studies. However, viral and bacterial efficacy studies showed a similar average LRVs of 3.5 and 3.3, respectively.

### Combination Treatment Approaches

#### Coagulation and Disinfection

Of the various combination treatment approaches that can be used for household drinking water treatment, there were numerous studies testing the LRV potential of several consumer products that have been developed to combine these two treatment steps: coagulation and disinfection. Typically these products function by using a coagulation step to chemically attract particles and flocculate to encourage sedimentation and simultaneously release a chemical disinfectant, often free chlorine, to inactivate microbes. The benefit of using these products includes ease of use, quality control/quality assurance performed during manufacturing to ensure functional product, and a relatively long and stable shelf life (e.g., 3 years). Within this category, we calculated the greatest LRV of 6.3 for bacteria, followed by a calculated LRV of 5.3 for viruses, and 2.6 LRV for protozoa. There were 18 studies included within this category. There were too few effectiveness studies for viral and protozoan classes to generate confidence intervals around LRV mean estimates.

### Summary

Individual LRVs by water treatment technology for bacteria, viruses, and protozoa are given in Figures 2, 3, and 4 and a combined figure comparing the boxplot distributions between treatment types is in Supporting Information Figure S1. Additional box-and-whisker plots were produced to differentiate bacterial, viral, and protozoan LRVs by household treatment technology and field versus lab studies in Figures S2–S4. Excluding coagulation/disinfection, for bacteria, carbon block technologies demonstrated the highest average LRV at 4.8, whereas granular media filtration showed the lowest mean of 1.9. Also again excluding coagulation/disinfection, viral targets had the highest average LRVs also with carbon block (5.0), compared to lowest mean LRVs with microfiltration (0.85). However, as previously noted, there were limited studies of carbon block filtration, with only four studies included in the analysis that included four data points for bacteria and 11 data points for viruses. When including 3 or more data points, protozoa had the greatest average LRV with ultrafiltration (4.5) and lowest mean LRV with chlorination (2.0), where the latter category excluded *Cryptosporidium* spp. as they are extremely resistant to chlorine. However, as previously indicated, there was more limited data for protozoa compared to bacteria and viruses and this is true for all treatment technologies.

**Figure 2:**
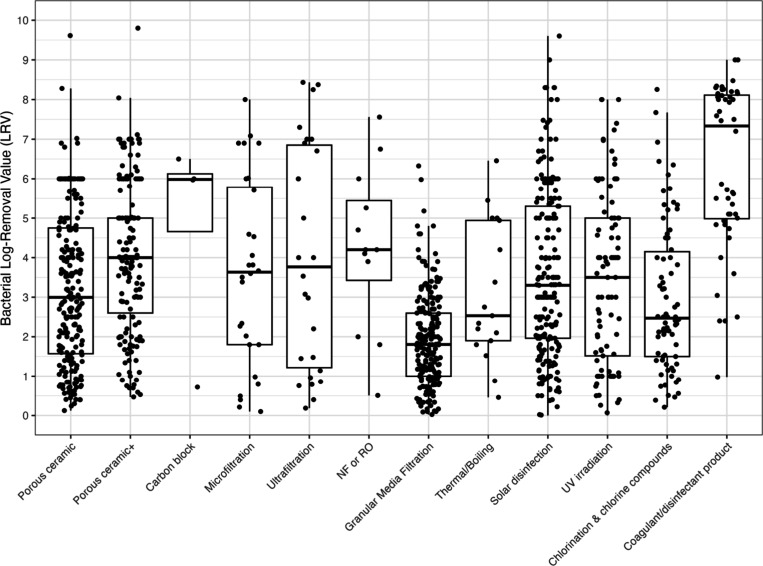
Bacterial LRVs by household water treatment type. Note: NF or RO – nanofiltration or reverse osmosis; UV – ultraviolet

**Figure 3:**
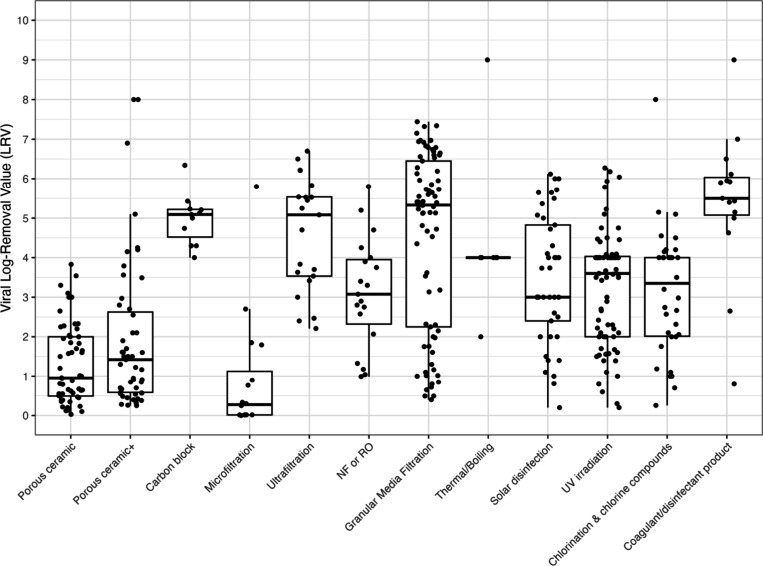
Viral LRVs by household water treatment type. Note: NF or RO – nanofiltration or reverse osmosis; UV – ultraviolet

**Figure 4:**
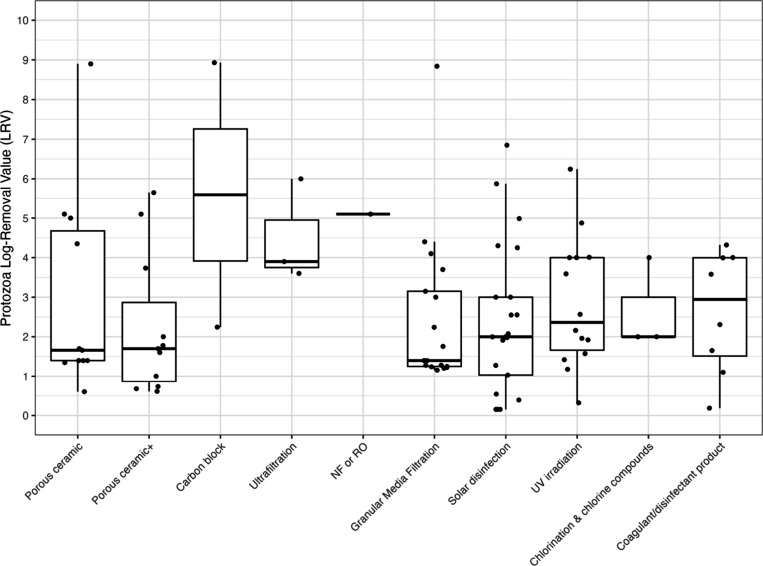
Protozoa LRVs by household water treatment type. Note: NF or RO – nanofiltration or reverse osmosis; UV – ultraviolet

We created a map to visualize where HWT performance trials were conducted and the number of articles included in this review from those countries (Supporting Information Figure S5). We encourage increased collaborations with local laboratories, universities, researchers, and practitioners in countries where HWT is most critical, as local data on local performance can be highly useful in evaluating HWT options with the greatest potential for local viability.

For most technologies, a range of variables influence specific LRVs, including the physical and chemical characteristics of the water being treated, specifics of the treatment technology or method and its variability, user behavior, and the specific microbe used in performance evaluation including the method for its measurement. For example, chlorine demand and turbidity are critical parameters that affect chemical disinfection effectiveness, and these variables may not be measured consistently across studies. Because of the underlying heterogeneity of microbial performance of HWT—including both measured and unmeasured factors—we observed a wide range of performance between technologies, across microbial classes, and even within similar methods for similar targets. While some findings are more intuitive, such as ultrafiltration performance being more effective at removing viral microbes than microfiltration, other findings also go against conventional wisdom such as the high LRVs associated with carbon block treatment alone for bacteria (mean LRV 4.8) and viruses (mean LRV 5.0). This is an example of the impact of what may be outlier values across a small number of data points (n = 4 for bacteria and n = 11 for viruses), resulting in estimates of mean LRVs that may not be representative. We urge caution in interpreting these results because of the wide variability we observed, particularly for categories where LRV estimates represent small numbers of studies and data points. Study-specific factors that may determine LRVs constrain our ability to compare individual estimates to one another, though we argue that, taken in the aggregate, these estimates are instructive in revealing the reported range of performance for key HWT technologies. While they show the reductions reported in the literature, they may not be representative of values that could be expected in practice. Published estimates may over-represent special cases (enhanced technologies or new applications of current technologies) or findings on the upper end of expected LRVs (reflecting possible publication bias). Because the science of drinking-water treatment is a mature discipline with many well-understood methods (e.g., chlorination with sodium hypochlorite, boiling), typical performance data is not considered novel and therefore may not be publishable, and would not appear in a systematic review informed mainly by peer-reviewed academic papers.

Previous estimates for boiling, based on expert review and a wider range of literature including pasteurization data, have assumed generally higher baseline LRVs for bacteria, viruses, and protozoa of 6,^5^ whereas our findings indicate a lower baseline LRV of 2.1 and 2.4 for bacteria and viruses, respectively, and with no data available for protozoa. Importantly, prior estimates solely considered efficacy of boiling while our analysis considered the practice of boiling, which included studies where boiling may not have been practiced consistently and correctly–e.g., when boiling was self-reported in surveys–and further included studies where recontamination occurred during storage and/or handling. In addition, for a number of technologies, the upper bound (95%) LRV determined for a technology from this review is substantively lower than the maximum LRV presented in Table 7.8 of the GDWQ for HWT technologies. While these values inherently represent different things, further studies on achievable maxima across technologies would be informative. Furthermore, granular media filtration from our review determined a specific maximum LRV for viruses at 5.0 compared with 4+ LRV outlined in Table 7.8 in the GDWQ for HWT technologies.^5^ A systematic review of the literature on microbial treatment performance of HWT shows that the variability of the technologies and the settings can result in a wide range of LRVs even for similar technologies. Acknowledging this variability is important given the need to recommend HWT options globally.

LRVs can guide evaluation and design criteria for future technologies. For example, if the purpose is to *evaluate* a system for safe water treatment, using the minimum LRV may be a conservative and defensible approach assuming adequate operation of treatment technology. If the purpose is to *design* a treatment system, perhaps the higher LRV range is most useful in representing optimal performance. While this work focuses on summarizing reported and calculated LRVs for a specific set of household water treatment technologies, the selection of technologies is beyond the scope of this paper and requires further guidance.^26^ The importance to assess individual products is useful because performance can vary even within the same technology groups. The WHO Scheme determines performance through systematic testing and transparent reporting, and additional research has indicated that consistent use even with basic efficacy can be critical to prevent waterborne infections.^26,30,31^

### Adoption beyond LRVs

There are many drivers beyond microbial performance that may influence adoption. Particularly where waterborne disease risk is high, consistent adherence to water treatment is the most critical factor.^26^ Risk modeling studies have demonstrated that HWT can only deliver meaningful reductions in risk when all or nearly all water consumed is treated.^16,80,81^ Adoption and correct, consistent, and sustained use of HWT can be influenced by the time required to use the method,^80^ the user interface, cost,^17^ long-term maintenance requirements,^81,82^ and the gendered nature of the user burden when it comes to household water management generally and HWT specifically.^17,83^ The selection of an HWT method or technology will be dependent upon all these variables as well as the cultural setting, current behaviors, availability of existing options, and willingness to adopt different methods. The optimal choice for an individual or household may come down to personal preferences as well, including taste, smell, or temperature of the treated water. HWT implementation requires a good understanding of what people want and will be willing and able to do consistently. Microbiological performance is meaningless if the technology is not used consistently, correctly, and sustainably.

### Limitations

This review has a number of important limitations. Updated LRVs indicate a variety of effective HWT options, but the generalizability of our findings is constrained by variable technology performance, the use of different microbial targets, variability across study settings, and a range of other factors that influence performance. The extreme heterogeneity and differences in quality and completeness of data across studies prevent a systematic examination of metadata to reveal systematic insights into why specific studies of similar technologies may have produced different results. Our goal has been to describe what is being reported–which is highly variable–rather than to establish a true or “correct” central tendency of LRV data by technology category and pathogen class. This reflects our motivation in this review to inform expected LRVs across a range of applications, which can be useful in technology assessment and recommendation, recognizing that different technologies in different settings will yield different performance. There are also several technical points that should be considered together with the LRV estimates we present in this review. First, the primary analysis focused on LRV arithmetic average values, which is affected by extreme or outlier LRVs. We used the 95% confidence interval to provide additional distribution context, because reporting of ranges would typically include extreme values on the high end and zero on the low end. We also included the IQR and provide a publicly available database (https://osf.io/jdeyx/) for further statistical analyses that may be useful, depending on the use case. Second, while we binned similar technologies together for the purposes of analysis, no such exercise is perfect and doing so obscures potentially important differences between and among closely related technologies. For example, we know that ceramic water filter design is highly variable and may be characterized by different flow rates, materials, and chemical amendments.^84^ Third, many studies included LRVs that were constrained by nondetects in post-treatment water, making LRVs a function of pretreatment water microbial counts only; this was widely prevalent in field-based trials of HWT and may explain the generally lower reported LRVs in that category. These LRVs may represent a lower bound for treatment performance overall, meaning that pooled mean LRV estimates including these values are likely to be conservative with respect to performance. Fourth, protozoan data were scarce for HWT technologies such as carbon block filtration, membrane filtration, and boiling (viral target data were also limited for boiling studies in the field (n = 0)). This review was constrained by the availability of data, and some targets were not widely assessed, either because the outcome of such testing would be straightforwardly induced (e.g., a filter relying on mechanical filtration might not test for protozoan reduction if it were shown to be effective for bacterial reduction based on size) or because there are known limitations of the technology or method that makes specific testing uninformative with respect to performance (e.g., standalone free chlorine disinfection to reduce *Cryptosporidium*). For these reasons, an absence of estimates in the peer-reviewed literature may not mean that we lack a good sense of potential reductions, but it means that such reductions would need to be inferred in addition to what is discoverable in the literature. This speaks to the need for expert judgment to augment the findings of this review, as it is necessarily limited by the sources of information we included (i.e., academic papers published in the peer-reviewed literature). This further implies that other reputable resources, in addition to academic papers, e.g. reports by government agencies, warrant consideration. It is also the case that some well characterized HWT methods, like boiling, are no longer widely represented in the peer-reviewed literature because their effectiveness is considered known and therefore of little interest in scientific discourse (reports of boiling performance from field trials would suggest otherwise, although it is recognized these studies largely have looked at evaluating boiling practices rather than efficacy of boiling *per se*, as previously discussed). Fifth, variability in test microbes and methods can influence performance, and the information reported in the literature on the state of microbes used in trials is often limited. There are also known differences between naturalized microbes in the environment versus those propagated under laboratory conditions, such as greater resistance to chlorine disinfection or increased capacity to handle stressors for environmental strains.^85,86^ Additional factors that may influence LRVs are microbial aggregation and microbe-particle association.^86^ Finally, publication bias may have influenced our results, resulting in a systematic review that is not representative of actual performance but that may be biased with the inclusion of novel, unexpected (e.g., outlier), or “positive” results showing good performance. We did not quantitatively assess these potential biases in this review, because we lacked consistent reporting of study sample size and precision estimates that would make such an analysis possible. Organizations and researchers writing about these technologies may sometimes be promoting or encouraging use of these technologies, which does not necessarily make the publications from an unbiased third party. Despite these limitations, the microbial performance estimates we report for HWT technologies can be an initial starting point for HWT selection and implementation, taken together as one input among many in recommending specific methods and technologies for specific contexts and conditions.

### Future Relevance and Applications of LRVs

Practitioners and researchers may use the LRV findings from this analysis as a future benchmark, in assessment of novel technologies, as a call for additional peer-reviewed literature on technologies with data issues (where there are gaps or where more research is needed to better understand the findings), and as a reference point for HWT technology performance. These methods may also be used in progressively updating the evidence as more data are available. Researchers may also use LRV point estimates and distributional measures of performance for quantitative microbial risk assessments (QMRAs).^87^

Considering the variable quality and quantity of data available for several technologies in this systematic review, these data may not fully capture LRVs expected from each technology. We recommend that our results be reviewed and considered alongside expert opinion and other reputable data sources, to inform the update of LRVs presented in the next GDWQ treatment table. However, given the variability in reported LRVs identified from this review and that achievable LRVs are context-specific, the need to provide a range in LRVs in the GDWQ, along with text encouraging localized assessment is critical. While we discuss the various LRV applications from lab- and field-based studies, it is imperative all stakeholders consider the importance of adherence: consistent, correct, and sustained use; adherence is key to delivering the expected health benefits of water quality interventions.^16,26,87–89^

### Reporting Guidelines

The quality and completeness of data and metadata between studies we reviewed were extremely variable. Critical metadata or minimum reporting guidelines accompanying treatment technology efficacy and effectiveness studies could lead to improved standardization of studies. We generated a checklist on suggested guidelines for studies to align with when assessing a pre- and post-treatment technology (Supporting Information Table S1). Major points to consider are the clear reporting of standard deviations or standard errors, if applicable; a comprehensive characterization of the challenge water and any water quality parameters measured; the reporting of any standard methods used for all measured parameters; and pre- and post-treatment raw microbial count values to calculate and confirm LRVs. Also, while graphs are useful to visualize pre- and post-treatment, we recommend a supplementary table that includes raw values and calculations performed to obtain LRVs.

### Emerging Technologies

In this review, we encountered many novel and emerging technologies that may have promise but are still undergoing rigorous testing, many from advances in materials science. For example, literature on novel biocoagulants and flocculation is expanding rapidly. Additionally, various nanoparticle-based technologies are becoming more common in membrane applications, including cellulose membranes, or as coagulant additives. While we excluded these promising new technologies *a priori*—and they do not appear in the GDWQ, and are not in current widespread use globally—they may represent an increasing share of HWT distribution in the future and should therefore be included in subsequent systematic reviews as evidence emerges. New methods and technology will become increasingly relevant and applicable as innovation proceeds in this area.

In summary, there is a large diversity of HWT technologies and approaches, most of which have sufficient data for LRV analysis, but some categories remain sparse. In binning the available published data by technology type, it is possible that important differences are masked based on testing conditions, methodological rigor, microbes, field settings for field studies, and other variables which may influence to performance data. The values determined through this study are a representation of peer-reviewed, published literature and may not be representative of “true” values. Finally, this review provides a replicable method for deriving performance data on HWT technologies. Future reviews may benefit from using a similar approach.

## Supplementary Material



## Notes

Additional results on combined LRVs for household water treatment types; individual pathogen class (bacterial, viral, protozoa) LRVs by field and lab studies; countries where household water treatment technologies were tested; PRISMA checklist and future performance checklist; LRV comparisons; IQR; search strings; and references (PDF)
