# Supplementary material for: Systematic Review of the Microbiological Performance of Household Water Treatment Technologies
Source: Environ Sci Technol. 2025 Mar 26;59(41):21776–89. doi: 10.1021/acs.est.4c03494 (PMC12550818; doi:10.1021/acs.est.4c03494)
Supplement: Supplementary file 1 [file es4c03494_si_001.pdf]

Supporting Information for

Systematic review of microbiological performance of household water treatment technologies

Gouthami Rao[1], Emma Wells[2], Catherine Reynolds[3], Rebecca Yoo[3], Erin Kowalsky[1], Jennifer DeFrance[4], Karl Linden[2], Joe Brown[1]*

[1] *Department of Environmental Sciences and Engineering, University of North Carolina – Chapel Hill, Chapel-Hill, NC, 27514, USA*

[2] *Department of Civil, Environmental, and Architectural Engineering, University of Colorado Boulder, Boulder, CO, 80303, USA*

[3] *Department of Civil and Environmental Engineering, Georgia Institute of Technology, Atlanta, GA, 30332, USA*

[4] *World Health Organization, Geneva, 1211, Switzerland*

***Corresponding author**: joebrown@unc.edu

Summary:

Number of Pages: 48

Number of Figures: 5 (Page S3–Page S7)

Number of Tables: 5 (Page S8–Page S17)

Number of Texts: 2 (Page S19–Page S21)

Table of Contents

Figure S1: Combined bacterial, protozoa, and viral LRVs across household water treatment types…………………………………………………………………………………………...S3

Figure S2: Bacterial LRVs across household water treatment types stratified by field and lab studies……………………………………………………………………………………....S4

Figure S3: Viral LRVs across household water treatment types stratified by field and lab studies……………………………………………………………………………………....S5

Figure S4: Protozoa LRVs across household water treatment types stratified by field and lab studies……………………………………………………………………………………S6

Figure S5: Countries where household water treatment technologies were tested for efficacy or effectiveness……………………………………………………………………….…S7

Table S1: Checklist of recommended minimum reporting requirements for future household water treatment technology performance evaluation…………………………..……………...S8

Table S2: PRISMA Checklist……………………………………………………………..S10

Table S3: The LRVs from the WHO's GDWQ (2022, 2008) compared to LRVs from this analysis…………………………………………………………………………………S13

Table S4: LRV interquartile ranges for household water treatment technology categories……S14

Table S5: Table 7.8 containing pathogen LRV guidelines from the WHO's last publication of the GDWQ…………………………………………………………………………………S17

Text S1: Search strings for Table 7.8……………………………………………………..S19

Text S2: References for Household Water Treatment Technologies…………………………..S21

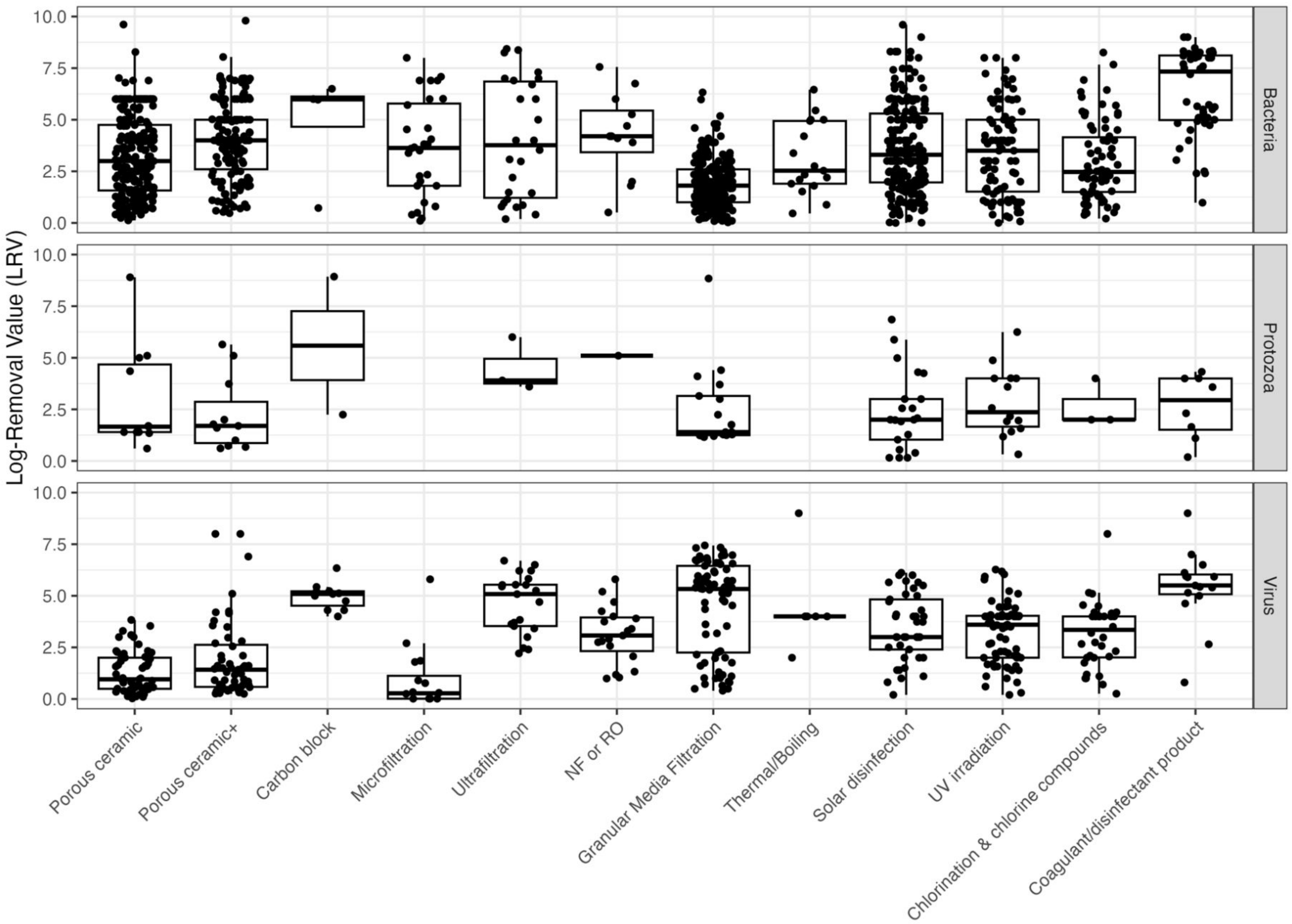

Figure S1. Combined bacterial, protozoa, and viral LRVs across household water treatment types

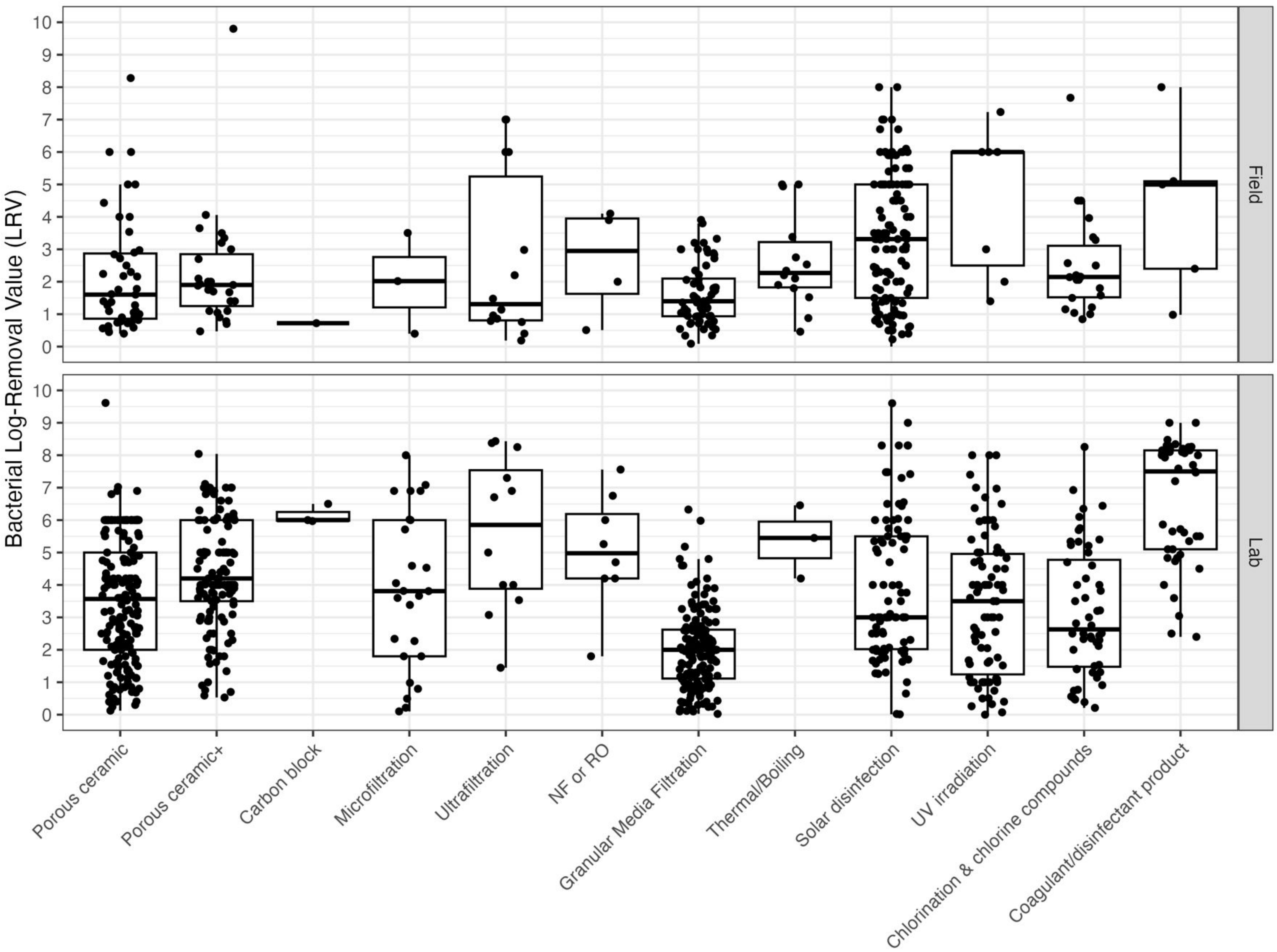

Figure S2. Bacterial LRVs across household water treatment types stratified by field and lab studies

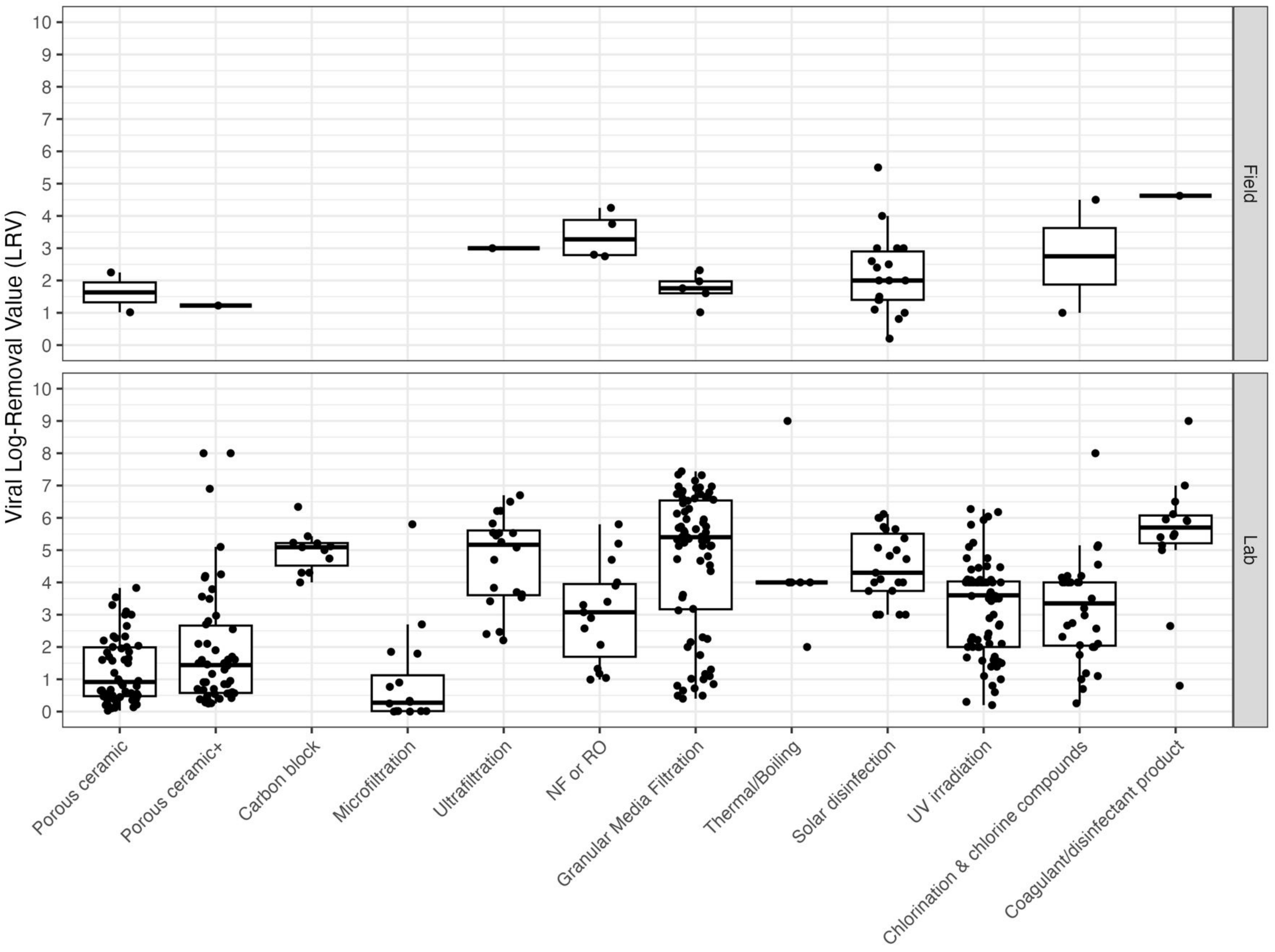

Figure S3. Viral LRVs across household water treatment types stratified by field and lab studies

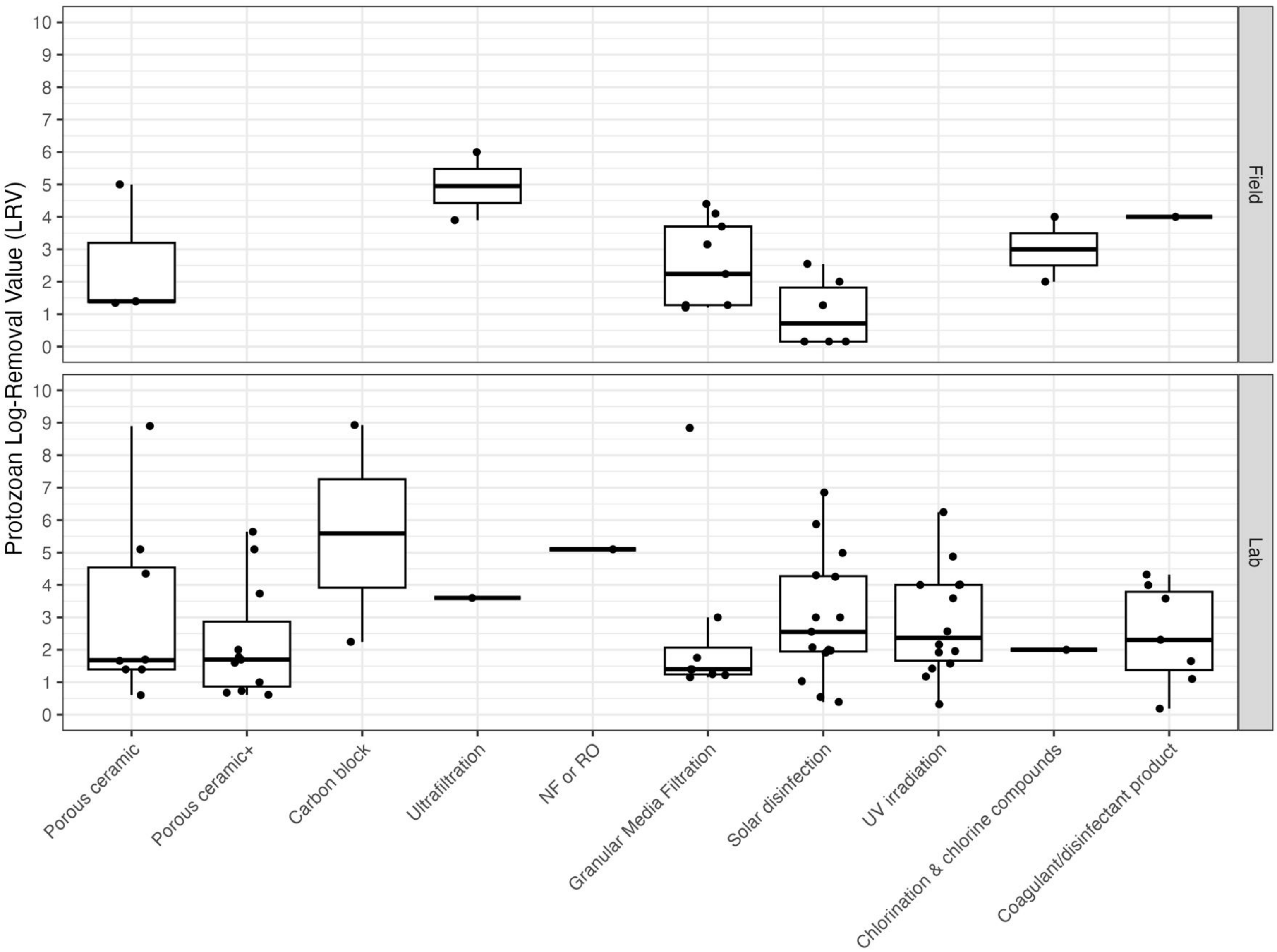

Figure S4. Protozoa LRVs across household water treatment types stratified by field and lab studies

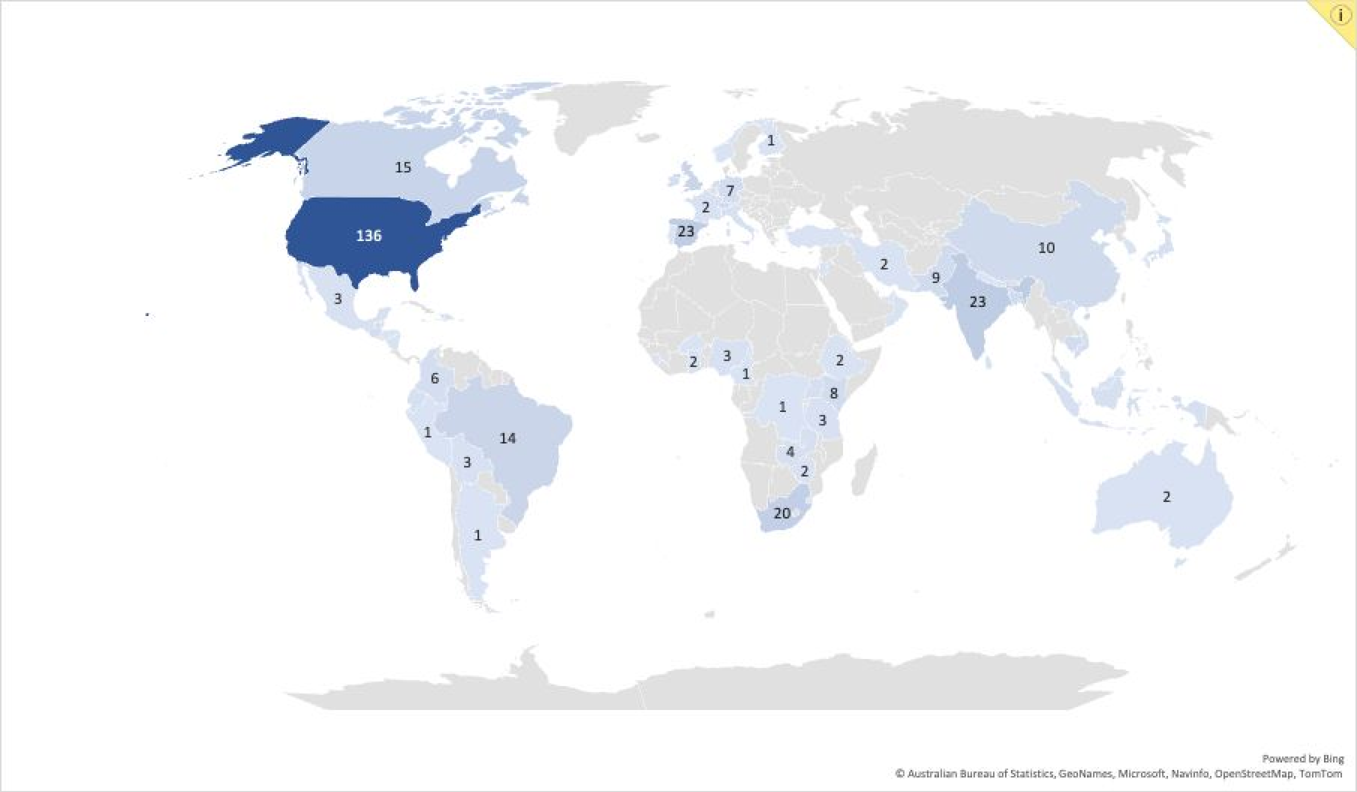

Figure S5. Countries where household water treatment technologies were tested for efficacy or effectiveness

Table S1: Checklist of recommended minimum reporting requirements for future household water treatment technology performance evaluation

Section	Item #	Checklist item	Location where included
Methods			
	1	Challenge water description	
	2	Water chemistry parameters defined and methods specified	
	3	Specific and technically accurate description of treatment technology, including known or suspected mechanism	
	4	Dose or CT and units	
	5	Target microbe; source; state (e.g., vegetative, spore); how isolated and handled and grown	
	6	Microbe in biofilm/aggregate or dispersed	
	7	Application context	
	8	Scale (household, etc)	
	9	Microbial assay type and reference, with appropriate methodological details (e.g., sufficient to meet EMMI/MIQE)	
	10	Number matched pre-post treatment pairs per unit	
	11	Number units reported	
	12	Analytical volume	
	13	LRV and indication of whether it is log10 or ln	
	14	Time over LRV demonstrated	
	15	Volume over LRV demonstrated	
	16	Positive controls reported	
	17	Negative controls reported	
	18	Limit of quantification/Limit of detection defined and reported	
	19	Specify geometric or arithmetic mean used in the analysis	
Results			
	20	Includes standard deviation of LRV	
	21	Includes standard error calculation	

22 95% confidence intervals of mean and/or median values
23 Report the final LRV from field or laboratory (in table format)
24 Report the limit of quantification
25 Report the limit of detection
26 Arithmetic mean pre-treatment count
27 Arithmetic mean post-treatment count
28 LRV limited by non-detect in product water
29 LRV limited by upper detection limit
30 LRV limited by lower detection limit

Table S2: PRISMA Checklist

Section and Topic	Item #	Checklist item	Location where item is reported
**TITLE**			
Title	1	Identify the report as a systematic review.	Title
**ABSTRACT**			
Abstract	2	See the PRISMA 2020 for Abstracts checklist.	Completed
**INTRODUCTION**			
Rationale	3	Describe the rationale for the review in the context of existing knowledge.	Introduction – lines 60-74
Objectives	4	Provide an explicit statement of the objective(s) or question(s) the review addresses.	Introduction – lines 69-74
**METHODS**			
Eligibility criteria	5	Specify the inclusion and exclusion criteria for the review and how studies were grouped for the syntheses.	Methods – lines 77-94
Information sources	6	Specify all databases, registers, websites, organisations, reference lists and other sources searched or consulted to identify studies. Specify the date when each source was last searched or consulted.	Methods – lines 77-94
Search strategy	7	Present the full search strategies for all databases, registers and websites, including any filters and limits used.	See Supplementary Text S1
Selection process	8	Specify the methods used to decide whether a study met the inclusion criteria of the review, including how many reviewers screened each record and each report retrieved, whether they worked independently, and if applicable, details of automation tools used in the process.	Methods – lines 115-145
Data collection process	9	Specify the methods used to collect data from reports, including how many reviewers collected data from each report, whether they worked independently, any processes for obtaining or confirming data from study investigators, and if applicable, details of automation tools used in the process.	Methods – lines 115-145
Data items	10a	List and define all outcomes for which data were sought. Specify whether all results that were compatible with each outcome domain in each study were sought (e.g. for all measures, time points, analyses), and if not, the methods used to decide which results to collect.	Methods – lines 115-145
	10b	List and define all other variables for which data were sought (e.g. participant and intervention characteristics, funding sources). Describe any assumptions made about any missing or unclear information.	Methods – lines 115-145
Study risk of bias assessment	11	Specify the methods used to assess risk of bias in the included studies, including details of the tool(s) used, how many reviewers assessed each study and whether they worked independently, and if applicable, details of automation tools used in the process.	Methods – lines 82-83
Effect measures	12	Specify for each outcome the effect measure(s) (e.g. risk ratio, mean difference) used in the synthesis or presentation of results.	Methods – lines 146-158
Synthesis methods	13a	Describe the processes used to decide which studies were eligible for each synthesis (e.g. tabulating the study intervention characteristics and comparing against the planned groups for each synthesis (item #5)).	Methods – lines 146-158

Section and Topic	Item #	Checklist item	Location where item is reported
	13b	Describe any methods required to prepare the data for presentation or synthesis, such as handling of missing summary statistics, or data conversions.	Methods – lines 146-158
	13c	Describe any methods used to tabulate or visually display results of individual studies and syntheses.	Methods – lines 146-158
	13d	Describe any methods used to synthesize results and provide a rationale for the choice(s). If meta-analysis was performed, describe the model(s), method(s) to identify the presence and extent of statistical heterogeneity, and software package(s) used.	Methods – lines 146-158
	13e	Describe any methods used to explore possible causes of heterogeneity among study results (e.g. subgroup analysis, meta-regression).	Methods – lines 146-158
	13f	Describe any sensitivity analyses conducted to assess robustness of the synthesized results.	No additional sensitivity analysis conducted
Reporting bias assessment	14	Describe any methods used to assess risk of bias due to missing results in a synthesis (arising from reporting biases).	Did not assess.
Certainty assessment	15	Describe any methods used to assess certainty (or confidence) in the body of evidence for an outcome.	95% confidence intervals around the arithmetic means
**RESULTS**			
Study selection	16a	Describe the results of the search and selection process, from the number of records identified in the search to the number of studies included in the review, ideally using a flow diagram.	Results – Figure 1
	16b	Cite studies that might appear to meet the inclusion criteria, but which were excluded, and explain why they were excluded.	Methods – lines 87-94
Study characteristics	17	Cite each included study and present its characteristics.	See Supplementary References: Text S2
Risk of bias in studies	18	Present assessments of risk of bias for each included study.	Did not assess
Results of individual studies	19	For all outcomes, present, for each study: (a) summary statistics for each group (where appropriate) and (b) an effect estimate and its precision (e.g. confidence/credible interval), ideally using structured tables or plots.	See Table 1.
Results of syntheses	20a	For each synthesis, briefly summarise the characteristics and risk of bias among contributing studies.	See Table 1.
	20b	Present results of all statistical syntheses conducted. If meta-analysis was done, present for each the summary estimate and its precision (e.g. confidence/credible interval) and measures of statistical heterogeneity. If comparing groups, describe the direction of the effect.	See Table 1 and Figures 2-4.
	20c	Present results of all investigations of possible causes of heterogeneity among study results.	See original data.
	20d	Present results of all sensitivity analyses conducted to assess the robustness of the synthesized results.	Did not assess
Reporting biases	21	Present assessments of risk of bias due to missing results (arising from reporting biases) for each synthesis assessed.	Did not assess, but included in discussion points.
Certainty of evidence	22	Present assessments of certainty (or confidence) in the body of evidence for each outcome assessed.	Results – confidence intervals

Section and Topic	Item #	Checklist item	Location where item is reported
			included
**DISCUSSION**			
Discussion	23a	Provide a general interpretation of the results in the context of other evidence.	Discussion – lines 364-373
	23b	Discuss any limitations of the evidence included in the review.	Discussion – limitations section
	23c	Discuss any limitations of the review processes used.	Discussion – limitations section
	23d	Discuss implications of the results for practice, policy, and future research.	Discussion – “Reporting Guidelines” and “Emerging Technologies” sections
**OTHER INFORMATION**			
Registration and protocol	24a	Provide registration information for the review, including register name and registration number, or state that the review was not registered.	This review was not registered.
	24b	Indicate where the review protocol can be accessed, or state that a protocol was not prepared.	Official protocol was not prepared
	24c	Describe and explain any amendments to information provided at registration or in the protocol.	N/A
Support	25	Describe sources of financial or non-financial support for the review, and the role of the funders or sponsors in the review.	World Health Organization
Competing interests	26	Declare any competing interests of review authors.	No competing interests
Availability of data, code and other materials	27	Report which of the following are publicly available and where they can be found: template data collection forms; data extracted from included studies; data used for all analyses; analytic code; any other materials used in the review.	Available upon request

Table S3: The LRVs from the WHO's GDWQ (2022, 2008) compared to LRVs from this analysis

		LRVs Recommended by the WHO GDWQ		LRVs Found in this Study^		
**Technology**	**Pathogen Type**	**Minimum**	**Maximum**	**Lower 95% C.I.**	**Average**	**Upper 95% C.I.**
Porous ceramic	Bacteria	2	6	3	3.2	3.5
	Viruses	1	4	1	1.3	1.6
	Protozoa	4	6	1.3	3.0	4.7
Porous ceramic, including modification	Bacteria	2	6	3.7	4	4.3
	Viruses	1	4	1.4	1.9	2.5
	Protozoa	4	6	1.3	2.5	3.7
Carbon block	Bacteria	2	6	0.46	4.8	9.1
	Viruses	1	4	4.5	5	5.4
	Protozoa	4	6	†	5.6	†
Membrane, microfiltration	Bacteria	2	4	2.7	3.6	4.6
	Viruses	0	4	0.07	0.85	1.6
	Protozoa	2	6	*	*	*
Membrane, ultrafiltration	Bacteria	3	6	2.9	4	5.2
	Viruses	3	6	4	4.6	5.3
	Protozoa	3	6	1.3	4.5	7.7
Membrane, NF or RO	Bacteria	3	6	2.9	4.2	5.6
	Viruses	3	6	2.4	3.1	3.8
	Protozoa	3	6	†	5.1	†
Granular Media Filtration (Biosand (BSF) + slow sand filtration)	Bacteria	1	3	1.7	1.9	2
	Viruses	0.5	2	4	4.5	5
	Protozoa	2	4	1.5	2.5	3.5
Boiling	Bacteria	6	9+	2.1	3	3.9
	Viruses	6	9+	2.4	4.4	6.4
	Protozoa	6	9+	*	*	*
Solar Treatment (solar UV radiation + thermal, solar photocatalysis)	Bacteria	3	5+	3.3	3.6	3.9
	Viruses	2	4+	3	3.5	4
	Protozoa	2	4+	1.6	2.4	3.3
UV irradiation	Bacteria	3	5+	3	3.5	3.9
	Viruses	2	5+	3	3.4	3.8
	Protozoa	3	5+	1.9	2.8	3.8
Chlorination and chlorine compounds	Bacteria	3	6	2.6	3.1	3.5
	Viruses	3	6	2.7	3.4	4.1
	Protozoa (non-*Cryptosporidium*)	3	5	-0.69	2	4.6
Coagulation/disinfection (e.g., commercial powder sachets or tablets)	Bacteria	7	9	5.8	6.3	6.9
	Viruses	4.5	6	4.4	5.3	6.2
	Protozoa	3	5	2.6	2.6	3.9

CI = Confidence interval
* = no studies found for this technology and/or pathogen type
^ = All mean LRVs reported are combined efficacy and effectiveness data
† = Any studies with 3 or fewer data points do not have CI's calculated

(green) = increased
(red) = decreased
(grey) = no change

Table S4: LRV interquartile ranges for household water treatment technology categories

Treatment process	Pathogen group*	Total number of studies	All - 25th percentile	All - 75th percentile	Laboratory - 25th percentile	Laboratory - 75th percentile	Field - 25th percentile	Field - 75th percentile
**Porous ceramic, carbon block or membrane filtration**								
Porous ceramic	Bacteria	75	1.5	4.7	2.0	5.0	0.81	2.9
	Viruses		0.49	2.0	0.48	2.0	*	*
	Protozoa		1.4	4.7	1.4	4.5	1.4	3.2
Porous ceramic, including modification	Bacteria	49	2.5	5.0	3.5	6.0	1.1	2.8
	Viruses		0.59	2.7	0.59	2.8	*	*
	Protozoa		0.93	4.1	0.93	4.1	*	*
Carbon block	Bacteria	4	4.7	6.1	6.0	6.3	*	*
	Viruses		4.5	5.2	4.5	5.2	*	*
	Protozoa		*	*	*	*	*	*
Membrane, microfiltration	Bacteria	14	1.8	5.8	1.8	6.0	1.2	2.8
	Viruses		0.01	0.90	0.01	0.90	0	***
	Protozoa		0	***	0	***	0	***
Membrane, ultrafiltration	Bacteria	21	1.2	6.9	3.9	7.5	0.81	5.2
	Viruses		3.5	5.5	3.6	5.6	*	*
	Protozoa		3.8	5.0	*	*	*	*
Membrane, NF or RO	Bacteria	10	3.4	5.4	4.2	6.2	1.6	4.0
	Viruses		2.3	4.0	1.7	4.9	2.8	3.9
	Protozoa		*	*	*	*	*	*

**Granular media filtration**									
Granular Media Filtration (Biosand (BSF) + slow sand filtration)	Bacteria	73	1.0	2.6	1.1	2.6	0.93	2.1
	Viruses		2.3	6.5	3.2	6.5	1.6	2.0
	Protozoa		1.3	3.2	1.2	2.1	1.3	3.7
**Thermal (heat) treatment**									
Boiling	Bacteria	17	1.8	4.8	4.8	6.0	1.7	3.1
	Viruses		4.0	4.0	4.0	4.0	*	*
	Protozoa		*	*	*	*	*	*
**Solar disinfection**									
Solar Treatment (solar UV radiation + thermal, solar photocatalysis)	Bacteria	86	2.0	5.3	2.0	5.5	1.5	5.0
	Viruses		2.4	4.8	3.7	5.5	1.4	2.9
	Protozoa		1.0	3.0	1.9	4.3	0.15	1.8
**UV Irradiation**									
UV irradiation (UV lamps + UV LED)	Bacteria	64	1.5	5.0	1.2	5.0	2.5	6.0
	Viruses		2.0	4.0	2.0	4.0	*	*
	Protozoa		1.7	4.0	1.7	4.0	*	*
**Chemical disinfection**									
Chlorination and chlorine compounds****	Bacteria	39	1.5	4.5	1.5	5.2	1.5	3.1
	Viruses		2.0	4.0	2.1	4.0	*	*
	Protozoa (non-*Cryptosporidium*)		1.5	2.5	*	*	*	*
**Combination (multiple-barrier) treatment**									
Coagulation/disinfection (e.g., commercial powder sachets or tablets)	Bacteria	18	5.1	8.1	5.2	8.1	2.4	5.1

	Viruses		5.0	6.0	5.0	6.0	*	*
	Protozoa		1.5	4.0	1.4	3.8	*	*

*A 25th and 75th percentile was not calculated for categories with <3 data points

Table S5: Table 7.8 containing pathogen LRV guidelines from the WHO's last publication of the GDWQ[5]

Table 7.8 Reductions of bacteria, viruses and protozoa achieved by household water treatment technologies

Treatment process	Enteric pathogen group	Baseline removal (LRV)	Maximum removal (LRV)	Notes
**Chemical disinfection**				
Free chlorine disinfection	Bacteria	3	6	Free chlorine × contact time predicts efficacy; not effective against *Cryptosporidium* oocysts. Turbidity and chlorine-demanding solutes inhibit this process; hence, turbidity should be kept below 1 NTU to support effective disinfection. Where this is not practical, the aim should be to keep turbidities below 5 NTU, although disinfection should still be practiced if 5 NTU cannot be achieved. At turbidities of more than 1 NTU, higher chlorine doses or contact times will be required[a]
	Viruses	3	6	
	Protozoa, non-*Cryptosporidium*	3	5	
	Cryptosporidium	0	1	
**Membrane, porous ceramic or composite filtration**				
Porous ceramic and carbon block filtration	Bacteria	2	6	Varies with pore size, flow rate, filter medium and inclusion of augmentation with silver or other chemical agents
	Viruses	1	4	
	Protozoa	4	6	
Membrane filtration (microfiltration, ultrafiltration, nanofiltration, reverse osmosis)	Bacteria	2 MF; 3 UF, NF or RO	4 MF; 6 UF, NF or RO	Varies with membrane pore size, integrity of filter medium and filter seals, and resistance to chemical and biological ("grow-through") degradation; maximum reductions associated with filtered water turbidity of < 0.1 NTU[a]
	Viruses	0 MF; 3 UF, NF or RO	4 MF; 6 UF, NF or RO	
	Protozoa	2 MF; 3 UF, NF or RO	6 MF; 6 UF, NF or RO	
Fibre and fabric filtration (e.g. sari cloth filtration)	Bacteria	1	2	Particle or plankton association increases removal of microbes, notably copepod-associated guinea worm (*Dracunculus medinensis*) and plankton-associated *Vibrio cholerae*; larger protozoa (> 20 µm) may be removed; ineffective for viruses, dispersed bacteria and small protozoa (e.g. *Giardia intestinalis*, 8–12 µm, and *Cryptosporidium* 4–6 µm)
	Viruses	0	0	
	Protozoa	0	1	

Table 7.8 (continued)

Treatment process	Enteric pathogen group	Baseline removal (LRV)	Maximum removal (LRV)	Notes
**Granular media filtration**				
Rapid granular, diatomaceous earth, biomass and fossil fuel–based (granular and powdered activated carbon, wood and charcoal ash, burnt rice hulls, etc.) filters	Bacteria	1	4+	Varies considerably with media size and properties, flow rate and operating conditions; some options are more practical than others for use in developing countries
	Viruses	1	4+	
	Protozoa	1	4+	
Household-level intermittently operated slow sand filtration	Bacteria	1	3	Varies with filter maturity, operating conditions, flow rate, grain size and filter bed contact time
	Viruses	0.5	2	
	Protozoa	2	4	

Text S1: Search strings for Table 7.8

January 1st 1997 up to March 21, 2021

PubMed and Web of Science:

((((((water OR groundwater)) AND (adsorption OR advanced oxidation OR alum OR aluminium chloride OR aluminium sulfate OR aluminium sulphate OR aluminum chloride OR aluminum sulfate OR aluminum sulphate OR anthracite OR AOP OR aquifer recharge OR aquifer storage OR BAC OR boiling OR brominated compounds OR brominated hydantoinylated resins OR bromine OR bromo-chloro compounds OR calcium hydroxide OR carbon OR chitosan OR chloramine OR chlorinated OR chlorinated hydantoinylated resins OR chlorination OR chlorine OR chlorine dioxide OR coagulant OR coagulation OR collimated beam OR combination treatment OR copper OR diatomaceous earth OR disinfectant OR disinfection OR electrochemical OR electrolysis OR electrolytic OR electroporation OR ferrate OR ferric chloride OR ferric sulfate OR ferric sulphate OR filter OR filtration OR flocculant OR flocculation OR flotation OR forward osmosis OR heat OR hydrogen peroxide OR iodinated OR iodine compounds OR ion exchange OR iron OR lime OR membranes OR metals OR microfilter OR microfiltration OR microporous OR mixed media OR mixed oxidants OR mixed-oxidants OR monochloramine OR multi-barrier treatment OR multi-media OR multiple mechanisms OR nanofiltration OR oxidative catalysts OR ozonation OR ozone OR PAA OR peracetic acid OR perchlorate OR prechlorination OR peroxide OR porous media OR power ultrasound OR reverse osmosis OR sand OR sediment infiltration OR sedimentation OR silver OR soil infiltration OR soil passage OR solar OR staining OR thermal OR titanium dioxide OR ultrafiltration OR ultraviolet OR up conversion OR UV OR uvao OR uvaol OR uv-led OR zeolite)) AND (acanthamoeba OR acinetobacter OR adenovirus OR aerobic spores OR aeromonas OR aichi virus OR anaerobic spores OR ascaris OR astrovirus OR bacteria OR bacteriophage OR bacteroidales OR bacteroides OR bacteroides fragilis OR balantidium OR blastocystis OR burkholderia OR burkholderia OR calicivirus OR campylobacter OR chlamydia OR clostridium OR coliform OR coliphage OR coronavirus OR coxsackievirus OR cronobacter OR cryptosporidium OR cyanobacteria OR cyclospora OR cysts OR dracunculus medinensis OR ebola OR echovirus OR elizabethkingia OR entamoeba OR enteric virus OR enterobacter OR enterobacteriaceae OR enterococci OR enterococcus OR enterocytozoon OR enterovirus OR entamoeba OR escherichia OR faecal coliform OR fasciola OR FC OR fecal coliform OR francisella OR fungi OR giardia OR guinea worm OR HAV OR helicobacter OR hepatitis a OR hepatitis e OR herpesvirus OR HEV OR histoplasma OR isospora OR klebsiella OR kobuvirus OR legionella OR leptospira OR mamastrovirus OR microsporidia OR mycobacteria OR naegleria OR norovirus OR norwalk virus OR onchocerca OR oocyst OR orthomyxovirus OR papovavirus OR parechovirus OR pasteurella OR phage OR polio OR poliovirus OR polyomavirus OR protozoa OR pseudomonas OR reovirus OR reticuloendotheliosis virus OR rotavirus OR salmonella OR sapovirus OR sars OR schistosoma OR shigella OR somatic coliphage OR spores OR thermotolerant coliform OR toxoplasma OR tsukamurella OR TTC OR vibrio OR vibrio cholerae OR virus OR yersinia)) AND (household OR point of entry OR point of use OR decentralized OR emergency OR humanitarian)) AND (inactivation OR log kill OR lrv OR reduction OR removal OR removal efficiency OR treatment effect))

Agricola via ProQuest and SCOPUS:

(all(water) OR all(groundwater)) AND (all(adsorption) OR all("advanced oxidation") OR all(alum) OR all("aluminium chloride") OR all("aluminium sulfate") OR all("aluminium sulphate") OR all("aluminum chloride") OR all("aluminum sulfate") OR all("aluminum sulphate") OR all(anthracite) OR all(AOP) OR all("aquifer recharge") OR all("aquifer storage") OR all(BAC) OR all(boiling) OR all("brominated compounds") OR all("brominated hydantoinylated resins") OR all(bromine) OR all("bromo-chloro compounds") OR all("calcium hydroxide") OR all(carbon) OR all(chitosan) OR all(chloramine) OR all(chlorinated) OR all("chlorinated hydantoinylated resins") OR all(chlorination) OR all(Chlorine) OR all("chlorine dioxide") OR all(coagulant) OR all(coagulation) OR all("collimated beam") OR all("combination treatment") OR all(copper) OR all("diatomaceous earth") OR all(disinfectant) OR all(disinfection) OR all(electrochemical) OR all(electrolysis) OR all(electrolytic) OR all(electroporation) OR all(ferrate) OR all("ferric chloride") OR all("ferric sulfate") OR all("ferric sulphate") OR all(filter) OR all(filtration) OR all(flocculant) OR all(flocculation) OR all(flotation) OR all("forward osmosis") OR all(heat) OR all("hydrogen peroxide") OR all(iodinated) OR all("iodine compounds") OR all("Ion Exchange") OR all(iron) OR all(lime) OR all(membranes) OR all(metals) OR all(microfilter) OR all(microfiltration) OR all(microporous) OR all("mixed media") OR all("mixed oxidants") OR all("mixed-oxidants") OR all("monochloramine") OR all("multi-barrier treatment") OR all("multi-media") OR all("multiple mechanisms") OR all(nanofiltration) OR all("oxidative catalysts") OR all(ozonation) OR all(ozone) OR all(PAA) OR all("peracetic acid") OR all(perchlorinate) OR all(perchlorination) OR all(peroxide) OR all("porous media") OR all("power ultrasound") OR all("reverse osmosis") OR all(sand) OR all("sediment infiltration") OR all(sedimentation) OR all(silver) OR all("soil infiltration") OR all("soil passage") OR all(solar) OR all(straining) OR all(thermal) OR all("titanium dioxide") OR all(ultrafiltration) OR all(ultraviolet) OR all(upconversion) OR all(UV) OR all(UVAO) OR all(UVAOP) OR all("UV-LED") OR all(zeolite)) AND (all(Acanthamoeba) OR all(Acinetobacter) OR all(Adenovirus) OR all("Aerobic spores") OR all(Aeromonas) OR all(Aichivirus) OR all("Anaerobic spores") OR all(Ascaris) OR all(Astrovirus) OR all(Bacteria) OR all(Bacteriophage) OR all(Bacteroidales) OR all(Bacteroides) OR all("Bacteroides fragilis") OR all(Balantidium) OR all(Blastocystis) OR all(Burkholderia) OR all(Burkholderia) OR all(Calicivirus) OR all(Campylobacter) OR all(Chlamydia) OR all(Clostridium) OR all(Coliform) OR all(Coliphage) OR all(Coronavirus) OR all(Coxsackievirus) OR all(Cronobacter) OR all(Cryptosporidium) OR all(Cyanobacteria) OR all(Cyclospora) OR all(Cysts) OR all("Dracunculus medinensis") OR all(Ebola) OR all(Echovirus) OR all(Elizabethkingia) OR all(Entamoeba) OR all("Enteric virus") OR all(Enterobacter) OR all(Enterobacteriaceae) OR all(Enterococci) OR all(Enterococcus) OR all(Enterocytozoon) OR all(Enterovirus) OR all(Entomeba) OR all(Escherichia) OR all("Faecal coliform") OR all(Fasciola) OR all(FC) OR all("Fecal coliform") OR all(Francisella) OR all(Fungi) OR all(Giardia) OR all("Guinea worm") OR all(HAV) OR all(Helicobacter) OR all("Hepatitis A") OR all("Hepatitis E") OR all(Hepevirus) OR all(HEV) OR all(Histoplasma) OR all(Isospora) OR all(Klebsiella) OR all(Kobuvirus) OR all(Legionella) OR all(Leptospira) OR all(Mamastrovirus) OR all(Microsporidia) OR all(Mycobacteria) OR all(Naegleria) OR all(Norovirus) OR all("Norwalk virus") OR all(Onchocerca) OR all(Oocyst) OR all(Orthomyxovirus) OR all(Papovavirus) OR all(Parechovirus) OR all(Pasteurella) OR all(Phage) OR all(Polio) OR all(Poliovirus) OR all(Polyomavirus) OR all(Protozoa) OR all(Pseudomonas) OR all(Reovirus) OR all("Reticuloendotheliosis virus") OR all(Rotavirus) OR all(Salmonella) OR all(Sapovirus) OR all(SARS) OR all(Schistosoma) OR all(Shigella) OR all("Somatic coliphage") OR all(Spores) OR all("Thermotolerant coliform") OR all(Toxoplasma) OR all(Tsukamurella) OR all(TTC) OR all(Vibrio) OR all("Vibrio cholerae") OR all(Virus) OR all(Yersinia)) AND (all(Household) OR all("Point of entry") OR all("Point of use") OR all(Decentralized) OR all(Emergency) OR all(Humanitarian)) AND (all(inactivation) OR all("log kill") OR all(lrv) OR all(reduction) OR all(removal) OR all("removal efficiency") OR all("treatment effect"))

Text S2: References for Household Water Treatment Technologies

1. Chlorine[1–34]

2. Ceramic filters[1,7,9,11,15,16,23,23,28,32,33,35–117]

3. Carbon block[38,118–120]

4. Membrane Filtration:
    a. Microfiltration[28,60,121–129]
    b. Ultrafiltration[28,91,93,117,122,124,130–133,133–136]
    c. Nanofiltration or Reverse Osmosis[38,137,60,91,116,138,139,28]

5. Granular Media Filtration[36,37,140–144,38,145–147,39,148–169,11,170–175,120,79,176,18,177,81–83,178–183,93,184–186,23,187–192,28,193–197]
6. Thermal (Boil)[198–201,57,202–205,29,16,206,207,98,208,209,27]
7. Solar Treatment[210–252,205,253–277,23,278–282,28,283–286,27]
8. UV[287,218,288–290,4,291–297,238,298–306,12,307–310,205,29,311–321,22,322–331,28,284,332,333,285,334,27,335]
9. Combination - Coagulation/Disinfection[1,7,15,16,23,34,46,336–342]

References

(1) Albert, J.; Luoto, J.; Levine, D. End-User Preferences for and Performance of Competing POU Water Treatment Technologies among the Rural Poor of Kenya. *Environ. Sci. Technol.* **2010**, *44* (12), 4426–4432. https://doi.org/10.1021/es1000566.

(2) Bommer, A.; Böhler, O.; Johannsen, E.; Dobrindt, U.; Kuczius, T. Effect of Chlorine on Cultivability of Shiga Toxin Producing Escherichia Coli (STEC) and β-Lactamase Genes Carrying E. Coli and Pseudomonas Aeruginosa. *International Journal of Medical Microbiology* **2018**, *308* (8), 1105–1112. https://doi.org/10.1016/j.ijmm.2018.09.004.

(3) Braun, L.; Sylivester, Y. D.; Zerefa, M. D.; Maru, M.; Allan, F.; Zewge, F.; Emery, A. M.; Kinung'hi, S.; Templeton, M. R. Chlorination of Schistosoma Mansoni Cercariae. *PLoS Neglected Tropical Diseases* **2020**, *14* (8), 1–16. https://doi.org/10.1371/journal.pntd.0008665.

(4) Carratalà, A.; Bachmann, V.; Julian, T. R.; Kohn, T. Adaptation of Human Enterovirus to Warm Environments Leads to Resistance against Chlorine Disinfection. *Environ Sci Technol* **2020**, *54* (18), 11292–11300. https://doi.org/10.1021/acs.est.0c03199.

(5) Coulliette, A. D.; Peterson, L. A.; Mosberg, J. A. W.; Rose, J. B. Evaluation of a New Disinfection Approach: Efficacy of Chlorine and Bromine Halogenated Contact Disinfection for Reduction of Viruses and Microcystin Toxin. *Am. J. Trop. Med. Hyg.* **2010**, *82* (2), 279–288. https://doi.org/10.4269/ajtmh.2010.09-0279.

(6) Coulliette, A. D.; Enger, K. S.; Weir, M. H.; Rose, J. B. Risk Reduction Assessment of Waterborne Salmonella and Vibrio by a Chlorine Contact Disinfectant Point-of-Use Device. *Int J Hyg Environ Health* **2013**, *216* (3), 355–361. https://doi.org/10.1016/j.ijheh.2012.08.007.

(7) Crump, J. A.; Okoth, G. O.; Slutsker, L.; Ogaja, D. O.; Keswick, B. H.; Luby, S. P. Effect of Point-of-Use Disinfection, Flocculation and Combined Flocculation-Disinfection on

Drinking Water Quality in Western Kenya. *J. Appl. Microbiol.* **2004**, *97* (1), 225–231. https://doi.org/10.1111/j.1365-2672.2004.02309.x.
(8) Elmaksoud, S. A.; Patel, N.; Maxwell, S. L.; Sifuentes, L. Y.; Gerba, C. P. Use of Household Bleach for Emergency Disinfection of Drinking Water. *J Environ Health* **2014**, *76* (9), 22–25.
(9) Gerba, C. P.; Abd-Elmaksoud, S.; Newick, H.; El-Esnawy, N. A.; Barakat, A.; Ghanem, H. Assessment of Coliphage Surrogates for Testing Drinking Water Treatment Devices. *Food and Environmental Virology* **2014**, *7* (1), 27–31. https://doi.org/10.1007/s12560-014-9173-1.
(10) Keithley, S. E.; Fakhreddine, S.; Kinney, K. A.; Kirisits, M. J. Effect of Treatment on the Quality of Harvested Rainwater for Residential Systems. *JOURNAL AMERICAN WATER WORKS ASSOCIATION* **2018**, *110* (7), E1–E11. https://doi.org/10.1002/awwa.1054.
(11) Lantagne, D.; Clasen, T. Effective Use of Household Water Treatment and Safe Storage in Response to the 2010 Haiti Earthquake. **2013**, *89* (3), 426–433. https://doi.org/10.4269/ajtmh.13-0179.
(12) Leifels, M.; Shoults, D.; Wiedemeyer, A.; Ashbolt, N. J.; Sozzi, E.; Hagemeier, A.; Jurzik, L. Capsid Integrity QPCR-An Azo-Dye Based and Culture-Independent Approach to Estimate Adenovirus Infectivity after Disinfection and in the Aquatic Environment. *WATER* **2019**, *11* (6). https://doi.org/10.3390/w11061196.
(13) Levy, K.; Anderson, L.; Robb, K. A.; Cevallos, W.; Trueba, G.; Eisenberg, J. N. S. Household Effectiveness vs. Laboratory Efficacy of Point-of-Use Chlorination. *Water Res.* **2014**, *54*, 69–77. https://doi.org/10.1016/j.watres.2014.01.037.
(14) McLaughlin, L. A.; Levy, K.; Beck, N. K.; Shin, G.-A.; Meschke, J. S.; Eisenberg, J. N. An Observational Study on the Effectiveness of Point-of-Use Chlorination. *J Environ Health* **2009**, *71* (8), 48–53.
(15) McLennan, S. D.; Peterson, L. A.; Rose, J. B. Comparison of Point-of-Use Technologies for Emergency Disinfection of Sewage-Contaminated Drinking Water. *Applied and environmental microbiology AEM.* **2009**, *75* (22), 7283–7286.
(16) Mohamed, H.; Clasen, T.; Njee, R. M.; Malebo, H. M.; Mbuligwe, S.; Brown, J. Microbiological Effectiveness of Household Water Treatment Technologies under Field Use Conditions in Rural Tanzania. *Trop. Med. Int. Health* **2016**, *21* (1), 33–40. https://doi.org/10.1111/tmi.12628.
(17) Morgenthau, A.; Nicolae, A. M.; Laursen, A. E.; Foucher, D. A.; Wolfaardt, G. M.; Hausner, M. Assessment of the Working Range and Effect of Sodium Dichloroisocyanurate on Pseudomonas Aeruginosa Biofilms and Planktonic Cells. *Biofouling* **2012**, *28* (1), 111–120. https://doi.org/10.1080/08927014.2011.654335.
(18) Murray, A. L.; Napotnik, J. A.; Rayner, J. S.; Mendoza, A.; Mitro, B.; Norville, J.; Faith, S. H.; Eleveld, A.; Jellison, K. L.; Lantagne, D. S. Evaluation of Consistent Use, Barriers to Use, and Microbiological Effectiveness of Three Prototype Household Water Treatment Technologies in Haiti, Kenya, and Nicaragua. *Sci Total Environ* **2020**, *718*, 134685. https://doi.org/10.1016/j.scitotenv.2019.134685.
(19) Murray, A. L.; Kumpel, E.; Peletz, R.; Khush, R. S.; Lantagne, D. S. The Effect of Sodium Thiosulfate Dechlorination on Fecal Indicator Bacteria Enumeration: Laboratory and Field Data. *Journal of water and health* **2018**, *16* (1), 70–77. https://doi.org/10.2166/wh.2017.077.

(20) Noguez, I. Y.; De Velásquez, M. T. O.; Reyes, K. A.; Garfias, M. B. M. Alternative Treatment to Remove Resistant Strains of Vibrio Cholerae in Water. *Journal of Environmental Engineering (United States)* **2020**, *146* (10). https://doi.org/10.1061/(ASCE)EE.1943-7870.0001795.
(21) Rashid, M.-U.; George, C. M.; Monira, S.; Mahmud, T.; Rahman, Z.; Mustafiz, M.; Saif-Ur-Rahman, K. M.; Parvin, T.; Bhuyian, S. I.; Zohura, F.; Begum, F.; Biswas, S. K.; Akhter, S.; Zhang, X.; Sack, D.; Sack, R. B.; Alam, M. Chlorination of Household Drinking Water Among Cholera Patients' Households to Prevent Transmission of Toxigenic Vibrio Cholerae in Dhaka, Bangladesh: CHoBI7 Trial. *Am. J. Trop. Med. Hyg.* **2016**, *95* (6), 1299–1304. https://doi.org/10.4269/ajtmh.16-0420.
(22) Edirisinghe, E. A. R.; Dissanayake, D. R. A.; Abayasekera, C. L.; Arulkanthan, A. Efficacy of Calcium Hypochlorite and Ultraviolet Irradiation against Mycobacterium Fortuitum and Mycobacterium Marinum. *Int. J. Mycobact.* **2017**, *6* (3), 311–314. https://doi.org/10.4103/ijmy.ijmy_88_17.
(23) Sobsey, M. D.; Stauber, C. E.; Casanova, L. M.; Brown, J. M.; Elliott, M. A. Point of Use Household Drinking Water Filtration: A Practical, Effective Solution for Providing Sustained Access to Safe Drinking Water in the Developing World. *Environmental Science and Technology* **2008**, *42* (12), 4261–4267. https://doi.org/10.1021/es702746n.
(24) Tang, A.; Bi, X.; Li, X.; Li, F.; Liao, X.; Zou, J.; Sun, W.; Yuan, B. The Inactivation of Bacteriophage MS2 by Sodium Hypochlorite in the Presence of Particles. *Chemosphere* **2021**, *266*. https://doi.org/10.1016/j.chemosphere.2020.129191.
(25) Venczel, L. V.; Likirdopulos, C. A.; Robinson, C. E.; Sobsey, M. D. Inactivation of Enteric Microbes in Water by Electro-Chemical Oxidant from Brine (NaCl) and Free Chlorine. *Water Sci. Technol.* **2004**, *50* (1), 141–146.
(26) Wilhelm, N.; Kaufmann, A.; Blanton, E.; Lantagne, D. Sodium Hypochlorite Dosage for Household and Emergency Water Treatment: Updated Recommendations. *J. Water Health* **2018**, *16* (1), 112–125. https://doi.org/10.2166/wh.2017.012.
(27) Zhong, Q.; Carratalà, A.; Ossola, R.; Bachmann, V.; Kohn, T. Cross-Resistance of UV- or Chlorine Dioxide-Resistant Echovirus 11 to Other Disinfectants. *Front Microbiol* **2017**, *8*, 1928. https://doi.org/10.3389/fmicb.2017.01928.
(28) World Health Organization. *Results of Round II of the WHO International Scheme to Evaluate Household Water Treatment Technologies*; World Health Organization: Geneva, 2019.
(29) Meister, S.; Verbyla, M. E.; Klinger, M.; Kohn, T. Variability in Disinfection Resistance between Currently Circulating Enterovirus B Serotypes and Strains. *Environ. Sci. Technol.* **2018**, *52* (6), 3696–3705. https://doi.org/10.1021/acs.est.8b00851.
(30) Luby, S.; Agboatwalla, M.; Raza, A.; Sobel, J.; Mintz, E.; Baier, K.; Rahbar, M.; Qureshi, S.; Hassan, R.; Ghouri, F.; Hoekstra, R. M.; Gangarosa, E. A Low-Cost Intervention for Cleaner Drinking Water in Karachi, Pakistan. *International Journal of Infectious Diseases* **2001**, *5* (3), 144–150. https://doi.org/10.1016/S1201-9712(01)90089-X.
(31) Quick, R. E.; Venczel, L. V.; Mintz, E. D.; Soleto, L.; Aparicio, J.; Gironaz, M.; Hutwagner, L.; Greene, K.; Bopp, C.; Maloney, K.; Chavez, D.; Sobsey, M.; Tauxe, R. V. Diarrhoea Prevention in Bolivia through Point-of-Use Water Treatment and Safe Storage: A Promising New Strategy. *Epidemiol. Infect.* **1999**, *122* (1), 83–90. https://doi.org/10.1017/S0950268898001782.

(32) Mellor, J. E.; Kallman, E.; Oyanedel-Craver, V.; Smith, J. A. Comparison of Three Household Water Treatment Technologies in San Mateo Ixtatan, Guatemala. *J. Environ. Eng.-ASCE* **2015**, *141* (5), 04014085. https://doi.org/10.1061/(ASCE)EE.1943-7870.0000914.
(33) Yoon, Y. J.; Kwon, M. H.; Jung, Y. M.; Moon, J. H.; Kang, J. W. Development of Point-of-Use Water Disinfection Technology Using Ceramicwater Filter and Electrochemical Hybrid System. *Water Sci. Technol.-Water Supply* **2013**, *13* (4), 1174–1180. https://doi.org/10.2166/ws.2013.124.
(34) Powers, E. M.; Hernandez, C.; Boutros, S. N.; Harper, B. G. Biocidal Efficacy of a Flocculating Emergency Water Purification Tablet. *Appl Environ Microbiol* **1994**, *60* (7), 2316–2323. https://doi.org/10.1128/aem.60.7.2316-2323.1994.
(35) Abebe, L. S.; Su, Y.-H.; Guerrant, R. L.; Swami, N. S.; Smith, J. A. Point-of-Use Removal of Cryptosporidium Parvum from Water: Independent Effects of Disinfection by Silver Nanoparticles and Silver Ions and by Physical Filtration in Ceramic Porous Media. *Environ. Sci. Technol.* **2015**, *49* (21), 12958–12967. https://doi.org/10.1021/acs.est.5b02183.
(36) Achio, S.; Kutsanedzie, F.; Ameko, E. Comparative Analysis on the Effectiveness of Various Filtration Methods on the Potability of Water. *Water Qual. Res. J. Canada* **2016**, *51* (1), 42–46. https://doi.org/10.2166/wqrjc.2015.014.
(37) Adeyemo, F. E.; Kamika, I.; Momba, M. N. B. Comparing the Effectiveness of Five Low-Cost Home Water Treatment Devices for Cryptosporidium, Giardia and Somatic Coliphages Removal from Water Sources. *Desalin. Water Treat.* **2015**, *56* (9), 2351–2367. https://doi.org/10.1080/19443994.2014.960457.
(38) Alsulaili, A.; Al-Harbi, M.; Elsayed, K. The Influence of Household Filter Types on Quality of Drinking Water. *Process Safety and Environmental Protection* **2020**, *143*, 204–211. https://doi.org/10.1016/j.psep.2020.06.051.
(39) Baumgartner, J.; Murcott, S.; Ezzati, M. Reconsidering "Appropriate Technology": The Effects of Operating Conditions on the Bacterial Removal Performance of Two Household Drinking-Water Filter Systems. *Environ. Res. Lett.* **2007**, *2* (2), 024003. https://doi.org/10.1088/1748-9326/2/2/024003.
(40) Bielefeldt, A. R.; Kowalski, K.; Summers, R. S. Bacterial Treatment Effectiveness of Point-of-Use Ceramic Water Filters. *Water Res.* **2009**, *43* (14), 3559–3565. https://doi.org/10.1016/j.watres.2009.04.047.
(41) Brown, J.; Sobsey, M. D.; Loomis, D. Local Drinking Water Filters Reduce Diarrheal Disease in Cambodia: A Randomized, Controlled Trial of the Ceramic Water Purifier. *Am. J. Trop. Med. Hyg.* **2008**, *79* (3), 394–400.
(42) Brown, J.; Sobsey, M. D. Ceramic Media Amended with Metal Oxide for the Capture of Viruses in Drinking Water. *Environmental Technology* **2009**, *30* (4), 379–391. https://doi.org/10.1080/09593330902753461.
(43) Brown, J.; Sobsey, M. D. Microbiological Effectiveness of Locally Produced Ceramic Filters for Drinking Water Treatment in Cambodia. *J Water Health* **2010**, *8* (1), 1–10. https://doi.org/10.2166/wh.2009.007.
(44) Brown, J.; Chai, R.; Wang, A.; Sobsey, M. D. Microbiological Effectiveness of Mineral Pot Filters in Cambodia. *Environ. Sci. Technol.* **2012**, *46* (21), 12055–12061. https://doi.org/10.1021/es3027852.

(45) Brown, D.; Farrow, C.; McBean, E. A.; Gharabaghi, B.; Beauchamp, J. Advancing Performance Evaluation Standards for Household Water Treatment Technologies. *Journal of Water and Health* **2019**, *17* (2), 266–273. https://doi.org/10.2166/wh.2018.266.
(46) Casanova, L. M.; Sobsey, M. D. Reduction of Acid-Fast and Non-Acid-Fast Bacteria by Point of Use Coagulation-Flocculation-Disinfection. *Int J Environ Res Public Health* **2015**, *12* (11), 14420–14428. https://doi.org/10.3390/ijerph121114420.
(47) Clark, K. N.; Elmore, A. C. Bacteria Removal Effectiveness of Ceramic Pot Filters Not Applied with Colloidal Silver. *Water Sci. Technol.-Water Supply* **2011**, *11* (6), 765–772. https://doi.org/10.2166/ws.2011.012.
(48) Clasen, T.; Menon, S. Microbiological Performance of Common Water Treatment Devices for Household Use in India. *Int J Environ Health Res* **2007**, *17* (2), 83–93. https://doi.org/10.1080/09603120701217695.
(49) Ehdaie, B.; Krause, C.; Smith, J. A. Porous Ceramic Tablet Embedded with Silver Nanopatches for Low-Cost Point-of-Use Water Purification. *Environ. Sci. Technol.* **2014**, *48* (23), 13901–13908. https://doi.org/10.1021/es503534c.
(50) Ehdaie, B.; Rento, C. T.; Son, V.; Turner, S. S.; Samie, A.; Dillingham, R. A.; Smith, J. A. Evaluation of a Silver-Embedded Ceramic Tablet as a Primary and Secondary Point-of-Use Water Purification Technology in Limpopo Province, S. Africa. *PLoS ONE* **2017**, *12* (1), e0169502. https://doi.org/10.1371/journal.pone.0169502.
(51) Ehdaie, B.; Su, Y.-H.; Swami Nathan, S.; Smith James, A. Protozoa and Virus Disinfection by Silver- and Copper-Embedded Ceramic Tablets for Water Purification. *Journal of Environmental Engineering* **2020**, *146* (4), 04020015. https://doi.org/10.1061/(ASCE)EE.1943-7870.0001664.
(52) Ekpunobi, U. E.; Agbo, S. U.; Ajiwe, V. I. E. Evaluation of the Mixtures of Clay, Diatomite, and Sawdust for Production of Ceramic Pot Filters for Water Treatment Interventions Using Locally Sourced Materials. *Journal of Environmental Chemical Engineering* **2019**, *7* (1), 102791. https://doi.org/10.1016/j.jece.2018.11.036.
(53) Farrow, C.; McBean, E.; Huang, G.; Yang, A. L.; Wu, Y. C.; Liu, Z.; Dai, Z. N.; Fu, H. Y.; Cawte, T.; Li, Y. P. Ceramic Water Filters: A Point-of-Use Water Treatment Technology to Remove Bacteria from Drinking Water in Longhai City, Fujian Province, China. *JOURNAL OF ENVIRONMENTAL INFORMATICS* **2018**, *32* (2), 63–68. https://doi.org/10.3808/jei.201800388.
(54) Galvan, M.; deVictorica, J. Assessment of a Water Filtration Device for Household Use in Rural Communities in Mexico. *Water Sci. Technol.* **1997**, *35* (11–12), 65–69. https://doi.org/10.1016/S0273-1223(97)00236-9.
(55) Gardner, T. D.; Guggenberger, J. D. Use of Ceramic Pot Filter (CPF) Technology under Pressure in an in-Line Pumping System. *Water Supply* **2018**, *18* (3), 843–852. https://doi.org/10.2166/ws.2017.157.
(56) Guerrero-Latorre, L.; Rusinol, M.; Hundesa, A.; Garcia-Valles, M.; Martinez, S.; Joseph, O.; Bofill-Mas, S.; Girones, R. Development of Improved Low-Cost Ceramic Water Filters for Viral Removal in the Haitian Context. *J. Wate Sanit. Hyg. Dev.* **2015**, *5* (1), 28–38. https://doi.org/10.2166/washdev.2014.121.
(57) Guerrero-Latorre, L.; Balseca-Enriquez, P.; Moyota-Tello, C.; Bravo-Camino, R.; Davila-Chavez, S.; Bonifaz-Arcos, E.; Romero-Carpio, B.; Chico-Terán, M. Performance of Black Ceramic Water Filters and Their Implementation in Rural Ecuador. *Journal of*

Water, Sanitation and Hygiene for Development **2019**, *9* (4), 694–702. https://doi.org/10.2166/washdev.2019.185.
(58) Hansen, M. L.; Vieira, A.; Antizar-Ladislao, B. Efficacy of Ceramic Water Purifiers Coated with Colloidal Ag Doped TiO 2 against Polyaromatic Hydrocarbons and Pathogens in Sierra Leone. *Water Science and Technology: Water Supply* **2012**, *12* (2), 133–139. https://doi.org/10.2166/ws.2012.105.
(59) He, Y.; Huang, G.; An, C.; Huang, J.; Zhang, P.; Chen, X.; Xin, X. Reduction of Escherichia Coli Using Ceramic Disk Filter Decorated by Nano-TiO2: A Low-Cost Solution for Household Water Purification. *Sci. Total Environ.* **2018**, *616*, 1628–1637. https://doi.org/10.1016/j.scitotenv.2017.10.149.
(60) Hörman, A.; Rimhanen-Finne, R.; Maunula, L.; von Bonsdorff, C. H.; Rapala, J.; Lahti, K.; Hänninen, M. L. Evaluation of the Purification Capacity of Nine Portable, Small-Scale Water Purification Devices. *Water Sci. Technol.* **2004**, *50* (1), 179–183.
(61) Huang, J.; Huang, G.; An, C.; Xin, X.; Chen, X.; Zhao, Y.; Feng, R.; Xiong, W. Exploring the Use of Ceramic Disk Filter Coated with Ag/ZnO Nanocomposites as an Innovative Approach for Removing Escherichia Coli from Household Drinking Water. *Chemosphere* **2020**, *245*, 125545. https://doi.org/10.1016/j.chemosphere.2019.125545.
(62) Jackson, K. N.; Smith, J. A. A New Method for the Deposition of Metallic Silver on Porous Ceramic Water Filters. *Journal of Nanotechnology* **2018**, *2018*, 2573015. https://doi.org/10.1155/2018/2573015.
(63) Jackson, K. N.; Smith, J. A.; Edokpayi, J. N. New Method for the Deposition of Metallic Silver and Metallic Copper on Full-Size Porous Ceramic Water Filters. *Environmental Engineering Science* **2019**, *36* (1), 2–11. https://doi.org/10.1089/ees.2018.0149.
(64) Jackson Kathryn, N.; Kahler David, M.; Kucharska, I.; Rekosh, D.; Hammarskjold, M.-L.; Smith James, A. Inactivation of MS2 Bacteriophage and Adenovirus with Silver and Copper in Solution and Embedded in Ceramic Water Filters. *Journal of Environmental Engineering* **2020**, *146* (3), 04019130. https://doi.org/10.1061/(ASCE)EE.1943-7870.0001634.
(65) Kahler, D. M.; Koermer, N. T.; Reichl, A. R.; Samie, A.; Smith, J. A. Performance and Acceptance of Novel Silver-Impregnated Ceramic Cubes for Drinking Water Treatment in Two Field Sites: Limpopo Province, South Africa and Dodoma Region, Tanzania. *Water* **2016**, *8* (3), 95. https://doi.org/10.3390/w8030095.
(66) Kallman, E. N.; Oyanedel-Craver, V. A.; Smith, J. A. Ceramic Filters Impregnated with Silver Nanoparticles for Point-of-Use Water Treatment in Rural Guatemala. *J. Environ. Eng.-ASCE* **2011**, *137* (6), 407–415. https://doi.org/10.1061/(ASCE)EE.1943-7870.0000330.
(67) Kaufman, A. R.; Casanova, L. M.; Sobsey, M. D. Efficacy of a Ceramic Siphon Household Water Filter for Removal of Pathogenic Microorganisms: Lifespan Volume Test. *Journal of Water Sanitation and Hygiene for Development* **2011**, *1* (2), 102–111. https://doi.org/10.2166/washdev.2011.037.
(68) Kendarto, D. R.; Mulyawan, A.; Dwiratna NP, S.; Bafdal, N.; Suryadi, E. Effectiveness of Ceramics Water Filter Pots with Addition of Silver Nitrate to Reduce of Escherichia Coli Contents. *International Journal on Advanced Science, Engineering and Information Technology* **2019**, *9* (2), 526. https://doi.org/10.18517/ijaseit.9.2.7142.
(69) Lantagne, D.; Klarman, M.; Mayer, A.; Preston, K.; Napotnik, J.; Jellison, K. Effect of Production Variables on Microbiological Removal in Locally-Produced Ceramic Filters

for Household Water Treatment. *Int J Environ Health Res* **2010**, *20* (3), 171–187. https://doi.org/10.1080/09603120903440665.
(70) Lemons, A.; Branz, A.; Kimirei, M.; Hawkins, T.; Lantagne, D. Assessment of the Quality, Effectiveness, and Acceptability of Ceramic Water Filters in Tanzania. *Journal of Water, Sanitation and Hygiene for Development* **2016**, *6* (2), 195–204. https://doi.org/10.2166/washdev.2016.006.
(71) Lucier, K. J.; Dickson-Anderson, S. E.; Schuster-Wallace, C. J. Effectiveness of Silver and Copper Infused Ceramic Drinking Water Filters in Reducing Microbiological Contaminants. *J. Water Supply Res Technol.-Aqua* **2017**, *66* (7), 528–536. https://doi.org/10.2166/aqua.2017.028.
(72) Matthies, K.; Bitter, H.; Deobald, N.; Heinle, M.; Diedel, R.; Obst, U.; Brenner-Weiss, G. Morphology, Composition and Performance of a Ceramic Filter for Household Water Treatment in Indonesia. *Water Practice and Technology* **2015**, *10* (2), 361–370. https://doi.org/10.2166/wpt.2015.044.
(73) Meierhofer, R.; Bänziger, C.; Deppeler, S.; Kunwar, B.; Bhatta, M. From Water Source to Tap of Ceramic Filters—Factors That Influence Water Quality Between Collection and Consumption in Rural Households in Nepal. *IJERPH* **2018**, *15* (11), 2439. https://doi.org/10.3390/ijerph15112439.
(74) Meierhofer, R.; Odhiambo, A.; Oremo, J.; Rubli, P. Does Activated Silver Reduce Recontamination Risks in the Reservoirs of Ceramic Water Filters? *Water* **2019**, *11* (5). https://doi.org/10.3390/w11051108.
(75) Michen, B.; Meder, F.; Rust, A.; Fritsch, J.; Aneziris, C.; Graule, T. Virus Removal in Ceramic Depth Filters Based on Diatomaceous Earth. *Environ. Sci. Technol.* **2012**, *46* (2), 1170–1177. https://doi.org/10.1021/es2030565.
(76) Michen, B.; Fritsch, J.; Aneziris, C.; Graule, T. Improved Virus Removal in Ceramic Depth Filters Modified with MgO. *Environ. Sci. Technol.* **2013**, *47* (3), 1526–1533. https://doi.org/10.1021/es303685a.
(77) Morris, J. F.; Murphy, J.; Fagerli, K.; Schneeberger, C.; Jaron, P.; Moke, F.; Juma, J.; Ochieng, J. B.; Omore, R.; Roellig, D.; Xiao, L.; Priest, J. W.; Narayanan, J.; Montgomery, J. M.; Hill, V.; Mintz, E.; Ayers, T. L.; O'Reilly, C. E. A Randomized Controlled Trial to Assess the Impact of Ceramic Water Filters on Prevention of Diarrhea and Cryptosporidiosis in Infants and Young Children—Western Kenya, 2013. *The American Journal of Tropical Medicine and Hygiene* **2018**, *98* (5), 1260–1268. https://doi.org/10.4269/ajtmh.17-0731.
(78) Muhammad, N.; Sinha, R.; Krishnan, E. R.; Patterson, C. L. Ceramic Filter for Small System Drinking Water Treatment: Evaluation of Membrane Pore Size and Importance of Integrity Monitoring. *J. Environ. Eng.-ASCE* **2009**, *135* (11), 1181–1191. https://doi.org/10.1061/(ASCE)EE.1943-7870.0000084.
(79) Murphy, H. M.; McBean, E. A.; Farahbakhsh, K. A Critical Evaluation of Two Point-of-Use Water Treatment Technologies: Can They Provide Water That Meets WHO Drinking Water Guidelines? *J Water Health* **2010**, *8* (4), 611–630. https://doi.org/10.2166/wh.2010.156.
(80) Murphy, H. M.; Sampson, M.; McBean, E.; Farahbakhsh, K. Influence of Household Practices on the Performance of Clay Pot Water Filters in Rural Cambodia. *Desalination* **2009**, *248* (1–3), 562–569. https://doi.org/10.1016/j.desal.2008.05.102.

(81) Mwabi, J. K.; Mamba, B. B.; Momba, M. N. B. Removal of Escherichia Coli and Faecal Coliforms from Surface Water and Groundwater by Household Water Treatment Devices/Systems: A Sustainable Solution for Improving Water Quality in Rural Communities of the Southern African Development Community Region. *Int J Environ Res Public Health* **2012**, *9* (1), 139–170. https://doi.org/10.3390/ijerph9010139.
(82) Mwabi, J. K.; Adeyemo, F. E.; Mahlangu, T. O.; Mamba, B. B.; Brouckaert, B. M.; Swartz, C. D.; Offringa, G.; Mpenyana-Monyatsi, L.; Momba, M. N. B. Household Water Treatment Systems: A Solution to the Production of Safe Drinking Water by the Low-Income Communities of Southern Africa. *Phys. Chem. Earth* **2011**, *36* (14–15), 1120–1128. https://doi.org/10.1016/j.pce.2011.07.078.
(83) Mwabi, J. K.; Mamba, B. B.; Momba, M. N. B. Removal of Waterborne Bacteria from Surface Water and Groundwater by Cost-Effective Household Water Treatment Systems (HWTS): A Sustainable Solution for Improving Water Quality in Rural Communities of Africa. *Water SA* **2013**, *39* (4), 445–456. https://doi.org/10.4314/wsa.v39i4.2.
(84) Ndebele, N.; Edokpayi, J. N.; Odiyo, J. O.; Smith, J. A. Field Investigation and Economic Benefit of a Novel Method of Silver Application to Ceramic Water Filters for Point-Of-Use Water Treatment in Low-Income Settings. *WATER* **2021**, *13* (3). https://doi.org/10.3390/w13030285.
(85) Ngoc Dung, T. T.; Phan Thi, L.-A.; Nam, V. N.; Nhan, T. T.; Quang, D. V. Preparation of Silver Nanoparticle-Containing Ceramic Filter by in-Situ Reduction and Application for Water Disinfection. *Journal of Environmental Chemical Engineering* **2019**, *7* (3), 103176. https://doi.org/10.1016/j.jece.2019.103176.
(86) Nigay, P.-M.; Salifu, A. A.; Obayemi, J. D.; White, C. E.; Nzihou, A.; Soboyejo, W. O. Ceramic Water Filters for the Removal of Bacterial, Chemical, and Viral Contaminants. *J. Environ. Eng.* **2019**, *145* (10), 04019066. https://doi.org/10.1061/(ASCE)EE.1943-7870.0001579.
(87) Nigay, P. M.; Salifu, A. A.; Obayemi, J. D.; White, C. E.; Nzihou, A.; Soboyejo, W. O. Assessment of Ceramic Water Filters for the Removal of Bacterial, Chemical, and Viral Contaminants. *Journal of Environmental Engineering (United States)* **2020**, *146* (7). https://doi.org/10.1061/(ASCE)EE.1943-7870.0001749.
(88) Nighojkar, A. K.; Vijay, A.; Kumavat, A.; Gupta, S.; Satankar, R. K.; Plappally, A. Use of Marble and Iron Waste Additives for Enhancing Arsenic and E. Coli Contaminant Removal Capacity and Strength of Porous Clay Ceramic Materials for Point of Use Drinking Water Treatment. *DWT* **2019**, *157*, 290–302. https://doi.org/10.5004/dwt.2019.23553.
(89) Oyanedel-Craver, V. A.; Smith, J. A. Sustainable Colloidal-Silver-Impregnated Ceramic Filter for Point-of-Use Water Treatment. *Environ. Sci. Technol.* **2008**, *42* (3), 927–933.
(90) Perez-Vidal, A.; Patricia Rivera-Sanchez, S.; Janeth Florez-Elvira, L.; Antonio Silva-Leal, J.; Diaz-Gomez, J.; Fernanda Herrera-Cuero, L.; Lopez Botero, L. P. Removal of E. Coli and Salmonella in Pot Ceramic Filters Operating at Different Filtration Rates. *WATER RESEARCH* **2019**, *159*, 358–364. https://doi.org/10.1016/j.watres.2019.05.028.
(91) Pérez-Vidal, A.; Diaz-Gómez, J.; Castellanos-Rozo, J.; Usaquen-Perilla, O. L. Long-Term Evaluation of the Performance of Four Point-of-Use Water Filters. *Water Res.* **2016**, *98*, 176–182. https://doi.org/10.1016/j.watres.2016.04.016.
(92) Rahman, S.; Karim, M. R.; Mahmud, Z. H. Laboratory Investigation of the Microbiological Performance of Ceramic Water Filters as Household Water Treatment

Technology. In *7th Brunei International Conference on Engineering and Technology 2018 (BICET 2018)*; Institution of Engineering and Technology: Bandar Seri Begawan, Brunei, 2018; p 10 (4 pp.)-10 (4 pp.). https://doi.org/10.1049/cp.2018.1507.
(93) Rayner, J.; Murray, A.; Joseph, M.; Branz, A.; Lantagne, D. Evaluation of Household Drinking Water Filter Distribution Programs in Haiti. *Journal of Water, Sanitation and Hygiene for Development* **2016**, *6* (1), 42–54. https://doi.org/10.2166/washdev.2016.121.
(94) Rayner, J.; Zhang, H.; Schubert, J.; Lennon, P.; Lantagne, D.; Oyanedel-Craver, V. Laboratory Investigation into the Effect of Silver Application on the Bacterial Removal Efficacy of Filter Material for Use on Locally Produced Ceramic Water Filters for Household Drinking Water Treatment. *ACS Sustainable Chemistry & Engineering* **2013**, *1* (7), 737–745. https://doi.org/10.1021/sc400068p.
(95) Rivera-Sánchez, S. P.; Ocampo-Ibáñez, I. D.; Silva-Leal, J. A.; Flórez-Elvira, L. J.; Castaño-Hincapié, A. V.; Dávila-Estupiñan, A.; Martínez-Rivera, J. I.; Pérez-Vidal, A. A Novel Filtration System Based on Ceramic Silver-Impregnated Pot Filter Combined with Adsorption Processes to Remove Waterborne Bacteria. *Scientific Reports* **2020**, *10* (1). https://doi.org/10.1038/s41598-020-68192-y.
(96) Salsali, H.; McBean, E.; Brunsting, J. Virus Removal Efficiency of Cambodian Ceramic Pot Water Purifiers. *Journal of Water and Health* **2011**, *9* (2), 306–311. https://doi.org/10.2166/wh.2011.087.
(97) Salvinelli, C.; Elmore, A. C.; García Hernandez, B. R.; Drake, K. D. Ceramic Pot Filters Lifetime Study in Coastal Guatemala. *J Water Health* **2017**, *15* (1), 145–154. https://doi.org/10.2166/wh.2016.082.
(98) Sari, Y.; Alfian, A. R.; Respati, T.; Agustian, D.; Raksanagara, A. Comparison of Drinking Water Quality Following Boiling , Household Filtration and Water-Refill in Urban-Slum Area; 2019.
(99) Servi, A. T.; Kang, P. K.; Frey, D.; Murcott, S. *A Holistic Optimization Framework for Improving Ceramic Pot Filter Performance*; Ieee: New York, 2013.
(100) Shepard, Z. J.; Lux, E. M.; Oyanedel-Craver, V. A. Performance of Silver Nanoparticle-Impregnated Ovoid Ceramic Water Filters. *Environmental Science: Nano* **2020**, *7* (6), 1772–1780. https://doi.org/10.1039/d0en00115e.
(101) Simonis, J. J.; Basson, A. K. Manufacturing a Low-Cost Ceramic Water Filter and Filter System for the Elimination of Common Pathogenic Bacteria. *Phys. Chem. Earth* **2012**, *50–52*, 269–276. https://doi.org/10.1016/j.pce.2012.05.001.
(102) Simonis, J. J.; Basson, A. K. Manufacture of a Low-Cost Ceramic Microporous Filter for the Elimination of Microorganisms Causing Common Diseases. *J. Wate Sanit. Hyg. Dev.* **2013**, *3* (1), 42–50. https://doi.org/10.2166/washdev.2013.110.
(103) Simonis, J. J.; Basson, A. K.; Selepe, T. Removal of Microbes to World Health Organization Requirements Using a Locally Developed, Low Cost, Micro-Porous, Ceramic Water Filter. *J. Wate Sanit. Hyg. Dev.* **2014**, *4* (4), 620–624. https://doi.org/10.2166/washdev.2014.042.
(104) Singh, R.; Rento, C.; Son, V.; Turner, S.; Smith, J. A. Optimization of Silver Ion Release from Silver-Ceramic Porous Media for Household Level Water Purification. *Water* **2019**, *11* (4), 816. https://doi.org/10.3390/w11040816.
(105) Soliman, M. Y. M.; van Halem, D.; Medema, G. Virus Removal by Ceramic Pot Filter Disks: Effect of Biofilm Growth and Surface Cleaning. *Int J Hyg Environ Health* **2020**, *224*, 113438. https://doi.org/10.1016/j.ijheh.2019.113438.

(106) Soppe, A. I. A.; Heijman, S. G. J.; Gensburger, I.; Shantz, A.; van Halem, D.; Kroesbergen, J.; Wubbels, G. H.; Smeets, P. W. M. H. Critical Parameters in the Production of Ceramic Pot Filters for Household Water Treatment in Developing Countries. *J. Water Health* **2015**, *13* (2), 587–599. https://doi.org/10.2166/wh.2014.090.
(107) van der Laan, H.; van Halem, D.; Smeets, P. W. M. H.; Soppe, A. I. A.; Kroesbergen, J.; Wubbels, G.; Nederstigt, J.; Gensburger, I.; Heijman, S. G. J. Bacteria and Virus Removal Effectiveness of Ceramic Pot Filters with Different Silver Applications in a Long Term Experiment. *Water Res.* **2014**, *51*, 47–54. https://doi.org/10.1016/j.watres.2013.11.010.
(108) van Halem, D.; van der Laan, H.; Soppe, A. I. A.; Heijman, S. G. J. High Flow Ceramic Pot Filters. *Water Res.* **2017**, *124*, 398–406. https://doi.org/10.1016/j.watres.2017.07.045.
(109) Van Halem, D.; Heijman, S. G. J.; Soppe, A. I. A.; Van Dijk, J. C.; Amy, G. L. Ceramic Silver-Impregnated Pot Filters for Household Drinking Water Treatment in Developing Countries: Material Characterization and Performance Study. *Water Science and Technology: Water Supply* **2007**, *7* (5–6), 9–17. https://doi.org/10.2166/ws.2007.142.
(110) Wegmann, M.; Michen, B.; Luxbacher, T.; Fritsch, J.; Graule, T. Modification of Ceramic Microfilters with Colloidal Zirconia to Promote the Adsorption of Viruses from Water. *Water Res.* **2008**, *42* (6–7), 1726–1734. https://doi.org/10.1016/j.watres.2007.10.030.
(111) White, E. P.; Langenfeld, J. K.; Bradford, E. L.; Haywood, H. G.; Salvinelli, C.; Elmore, A. C. Assessment of Flow Rate as a Quality Control Test for Ceramic Pot Filters. *World Environmental and Water Resources Congress 2015: Floods, Droughts, and Ecosystems* **2015**, 1378–1387.
(112) Yakub, I.; Plappally, A.; Leftwich, M.; Malatesta, K.; Friedman, K. C.; Obwoya, S.; Nyongesa, F.; Maiga, A. H.; Soboyejo, A. B. O.; Logothetis, S.; Soboyejo, W. Porosity, Flow, and Filtration Characteristics of Frustum-Shaped Ceramic Water Filters. *Journal of Environmental Engineering (United States)* **2013**, *139* (7), 986–994. https://doi.org/10.1061/(ASCE)EE.1943-7870.0000669.
(113) Yang, H.; Min, X.; Xu, S.; Bender, J.; Wang, Y. Development of Effective and Fast-Flow Ceramic Porous Media for Point-of-Use Water Treatment: Effect of Pore Size Distribution. *ACS sustainable chemistry & engineering* **2020**, *8* (6), 2531–2539. https://doi.org/10.1021/acssuschemeng.9b07177.
(114) Zhang, H.; Oyanedel-Craver, V. Comparison of the Bacterial Removal Performance of Silver Nanoparticles and a Polymer Based Quaternary Amine Functiaonalized Silsesquioxane Coated Point-of-Use Ceramic Water Filters. *J. Hazard. Mater.* **2013**, *260*, 272–277. https://doi.org/10.1016/j.jhazmat.2013.05.025.
(115) Varkey, A. J.; Dlamini, M. D. Point-of-Use Water Purification Using Clay Pot Water Filters and Copper Mesh. *Water SA* **2012**, *38* (5), 721–726. https://doi.org/10.4314/wsa.v38i5.10.
(116) Sangsanont, J.; The Dan, D.; Thi Viet Nga, T.; Katayama, H.; Furumai, H. Detection of Pepper Mild Mottle Virus as an Indicator for Drinking Water Quality in Hanoi, Vietnam, in Large Volume of Water after Household Treatment. *J Environ Sci Health A Tox Hazard Subst Environ Eng* **2016**, *51* (13), 1100–1106. https://doi.org/10.1080/10934529.2016.1199650.
(117) Bettin, C.; Schwarz, B.; Kornmueller, A. Practical Challenge Testing of a Ceramic Membrane Module in a Full-Scale Mobile Drinking Water Treatment System. *J. Water Supply Res Technol.-Aqua* **2013**, *62* (3), 176–182. https://doi.org/10.2166/aqua.2013.126.

(118) Gerba, C. P.; Naranjo, J. E. Microbiological Water Purification without the Use of Chemical Disinfection. *Wilderness Environ Med* **2000**, *11* (1), 12–16.
(119) Gerba, C. P.; Naranjo, J. E.; Jones, E. L. Virus Removal from Water by a Portable Water Treatment Device. *Wildern. Environ. Med.* **2008**, *19* (1), 45–49. https://doi.org/10.1580/07-WEME-BR-109.1.
(120) Muhammad, N.; Sinha, R.; Krishnan, E. R.; Piao, H.; Patterson, C. L.; Cotruvo, J.; Cumberland, S. L.; Nero, V. P.; Delandra, C. Evaluating Surrogates for Cryptosporidium Removal in Point-of-Use Systems. *J. Am. Water Work Assoc.* **2008**, *100* (12), 98–107.
(121) da Silva, F. V.; Yamaguchi, N. U.; Lovato, G. A.; da Silva, F. A.; Miranda Reis, M. H.; Pessoa Sousa de Amorim, M. T.; Granhen Tavares, C. R.; Bergamasco, R. Effects of Coconut Granular Activated Carbon Pretreatment on Membrane Filtration in a Gravitational Driven Process to Improve Drinking Water Quality. *Environ. Technol.* **2012**, *33* (6), 711–716. https://doi.org/10.1080/09593330.2011.589133.
(122) Davey, J.; Schaefer, A. I. *Ultrafiltration to Supply Drinking Water in International Development: A Review of Opportunities*; Yanful, E. K., Ed.; Springer: Dordrecht, 2009.
(123) Hosseini, S. S.; Khodadadi, H.; Bakhshi, B. Fabrication, Tuning and Performance Analysis of Polyacrylonitrile (PAN)-Derived Microfiltration Membranes for Bacteria Removal from Drinking Water. *KOREAN JOURNAL OF CHEMICAL ENGINEERING* **2021**, *38* (1), 32–45. https://doi.org/10.1007/s11814-020-0666-3.
(124) Matsushita, T.; Shirasaki, N.; Tatsuki, Y.; Matsui, Y. Investigating Norovirus Removal by Microfiltration, Ultrafiltration, and Precoagulation-Microfiltration Processes Using Recombinant Norovirus Virus-like Particles and Real-Time Immuno-PCR. *Water research* **2013**, *47* (15), 5819–5827. https://doi.org/10.1016/j.watres.2013.07.004.
(125) Murray, A. L.; Stewart, B.; Hopper, C.; Tobin, E.; Rivera, J.; Mut-Tracy, H.; Stewart, P.; Stewart, C.; Tobin, C.; Goeb, M.; Meub, C.; Lantagne, D. S. Laboratory Efficacy and Field Effectiveness of Hollow Fiber Membrane Microfilters Used for Household Water Treatment in Honduras. *Journal of Water, Sanitation and Hygiene for Development* **2017**, *7* (1), 74–84. https://doi.org/10.2166/washdev.2017.156.
(126) Ensink, J. H. J.; Bastable, A.; Cairncross, S. Assessment of a Membrane Drinking Water Filter in an Emergency Setting. *J Water Health* **2015**, *13* (2), 362–370. https://doi.org/10.2166/wh.2014.025.
(127) Jordan, F. L.; Seaman, R.; Riley, J. J.; Yoklic, M. R. Effective Removal of Microbial Contamination from Harvested Rainwater Using a Simple Point of Use Filtration and UV-Disinfection Device. *Urban Water Journal* **2008**, *5* (3), 209–218. https://doi.org/10.1080/15730620801977174.
(128) Madaeni, S. S.; Fane, A. G.; Grohmann, G. S. Virus Removal from Water and Wastewater Using Membranes. *Journal of Membrane Science* **1995**, *102*, 65–75. https://doi.org/10.1016/0376-7388(94)00252-T.
(129) Sheffer, P. J.; Stout, J. E.; Wagener, M. M.; Muder, R. R. Efficacy of New Point-of-Use Water Filter for Preventing Exposure to Legionella and Waterborne Bacteria. *Am J Infect Control* **2005**, *33* (5 Suppl 1), S20-25. https://doi.org/10.1016/j.ajic.2005.03.012.
(130) Clasen, T.; Naranjo, J.; Frauchiger, D.; Gerba, C. Laboratory Assessment of a Gravity-Fed Ultrafiltration Water Treatment Device Designed for Household Use in Low-Income Settings. *Am. J. Trop. Med. Hyg.* **2009**, *80* (5), 819–823.
(131) Fagerli, K.; Gieraltowski, L.; Nygren, B.; Foote, E.; Gaines, J.; Oremo, J.; Odhiambo, A.; Kim, S.; Quick, R. Use, Acceptability, Performance, and Health Impact of Hollow Fiber

Ultrafilters for Water Treatment in Rural Kenyan Households, 2009-2011. *Am J Trop Med Hyg* **2020**, *103* (1), 465–471. https://doi.org/10.4269/ajtmh.19-0862.
(132) Peletz, R.; Simunyama, M.; Sarenje, K.; Baisley, K.; Filteau, S.; Kelly, P.; Clasen, T. Assessing Water Filtration and Safe Storage in Households with Young Children of HIV-Positive Mothers: A Randomized, Controlled Trial in Zambia. *PLoS One* **2012**, *7* (10), e46548. https://doi.org/10.1371/journal.pone.0046548.
(133) Zhang, X.; He, Y.; Zhang, B.; Qin, L.; Yang, Q.; Huang, H. Factors Affecting Microbiological Quality of Household Drinking Water Supplied by Small-Scale Ultrafiltration Systems: A Field Study. *Science of The Total Environment* **2019**, *689*, 725–733. https://doi.org/10.1016/j.scitotenv.2019.06.327.
(134) Boisson, S.; Kiyombo, M.; Sthreshley, L.; Tumba, S.; Makambo, J.; Clasen, T. Field Assessment of a Novel Household-Based Water Filtration Device: A Randomised, Placebo-Controlled Trial in the Democratic Republic of Congo. *PLoS ONE* **2010**, *5* (9), e12613. https://doi.org/10.1371/journal.pone.0012613.
(135) Francis, M. R.; Sarkar, R.; Roy, S.; Jaffar, S.; Mohan, V. R.; Kang, G.; Balraj, V. Effectiveness of Membrane Filtration to Improve Drinking Water: A Quasi-Experimental Study from Rural Southern India. *Am. J. Trop. Med. Hyg.* **2016**, *95* (5), 1192–1200. https://doi.org/10.4269/ajtmh.15-0675.
(136) Pooi, C. K.; Ng, H. Y. Review of Low-Cost Point-of-Use Water Treatment Systems for Developing Communities. *npj Clean Water* **2018**, *1* (1), 11. https://doi.org/10.1038/s41545-018-0011-0.
(137) Brady-Estévez, A. S.; Nguyen, T. H.; Gutierrez, L.; Elimelech, M. Impact of Solution Chemistry on Viral Removal by a Single-Walled Carbon Nanotube Filter. *Water Res.* **2010**, *44* (13), 3773–3780. https://doi.org/10.1016/j.watres.2010.04.023.
(138) Torii, S.; Hashimoto, T.; Do, A. T.; Furumai, H.; Katayama, H. Impact of Repeated Pressurization on Virus Removal by Reverse Osmosis Membranes for Household Water Treatment. *Environ. Sci.: Water Res. Technol.* **2019**, *5* (5), 910–919. https://doi.org/10.1039/C8EW00944A.
(139) Torii, S.; Hashimoto, T.; Do, A. T.; Furumai, H.; Katayama, H. Repeated Pressurization as a Potential Cause of Deterioration in Virus Removal by Aged Reverse Osmosis Membrane Used in Households. *Science of The Total Environment* **2019**, *695*, 133814. https://doi.org/10.1016/j.scitotenv.2019.133814.
(140) Ahammed, M. M.; Meera, V. Metal Oxide/Hydroxide-Coated Dual-Media Filter for Simultaneous Removal of Bacteria and Heavy Metals from Natural Waters. *J. Hazard. Mater.* **2010**, *181* (1–3), 788–793. https://doi.org/10.1016/j.jhazmat.2010.05.082.
(141) Ahammed, M. M.; Davra, K. Performance Evaluation of Biosand Filter Modified with Iron Oxide-Coated Sand for Household Treatment of Drinking Water. *Desalination.* **2011**, *276* (1–3), 287–293.
(142) Ahammed, M. M.; Meera, V. Iron Hydroxide-Coated Sand Filter for Household Drinking Water from Roof-Harvested Rainwater. *J. Water Supply Res Technol.-Aqua* **2006**, *55* (7–8), 493–498. https://doi.org/10.2166/aqua.2006.052.
(143) Mansoor Ahammed, M.; Chaudhuri, M. A Low-Cost Home Water Filter. *Journal of Water Supply: Research and Technology—AQUA* **1999**, *48* (6), 263–267. https://doi.org/10.2166/aqua.1999.0029.

(144) Aiken, B. A.; Stauber, C. E.; Ortiz, G. M.; Sobsey, M. D. An Assessment of Continued Use and Health Impact of the Concrete Biosand Filter in Bonao, Dominican Republic. *Am. J. Trop. Med. Hyg.* **2011**, *85* (2), 309–317. https://doi.org/10.4269/ajtmh.2011.09-0122.
(145) Andreoli, F. C.; Sabogal-Paz, L. P. Household Slow Sand Filter to Treat Groundwater with Microbiological Risks in Rural Communities. *Water Research* **2020**, *186*. https://doi.org/10.1016/j.watres.2020.116352.
(146) Arnold, N.; Archer, A.; Barkdoll, B. Bacterial Adaptation and Performance of Household Biosand Water Filters in Differing Temperatures. *Water Sci. Technol.-Water Supply* **2016**, *16* (3), 794–801. https://doi.org/10.2166/ws.2015.192.
(147) Baig, S. A.; Mahmood, Q.; Nawab, B.; Shafqat, M. N.; Pervez, A. Improvement of Drinking Water Quality by Using Plant Biomass through Household Biosand Filter - A Decentralized Approach. *Ecol. Eng.* **2011**, *37* (11), 1842–1848. https://doi.org/10.1016/j.ecoleng.2011.06.011.
(148) Bradley, I.; Straub, A.; Maraccini, P.; Markazi, S.; Nguyen, T. H. Iron Oxide Amended Biosand Filters for Virus Removal. *Water Res.* **2011**, *45* (15), 4501–4510. https://doi.org/10.1016/j.watres.2011.05.045.
(149) Curry, K. D.; Morgan, M.; Peang, S. H.; Seang, S. Biosand Water Filters for Floating Villages in Cambodia: Safe Water Does Not Prevent Recontamination. *J. Wate Sanit. Hyg. Dev.* **2015**, *5* (2), 213–219. https://doi.org/10.2166/washdev.2015.120.
(150) D'Alessio, M.; El-Swaify, G.; Yoneyama, B.; Ray, C. A Low-Cost Water-Treatment System for Potable Water Supplies in Developing Countries and after a Natural Disaster: Ability to Remove Total Coliforms and E-Coli. *Clean Technol. Environ. Policy* **2016**, *18* (3), 925–934. https://doi.org/10.1007/s10098-015-1074-y.
(151) Danley-Thomson, A. A.; Huang, E. C.; Worley-Morse, T.; Gunsch, C. K. Evaluating the Role of Total Organic Carbon in Predicting the Treatment Efficacy of Biosand Filters for the Removal of *Vibrio Cholerae* in Drinking Water during Startup. *J Appl Microbiol* **2018**, *125* (3), 917–928. https://doi.org/10.1111/jam.13909.
(152) Duke, W. F.; Nordin, R. N.; Baker, D.; Mazumder, A. The Use and Performance of BioSand Filters in the Artibonite Valley of Haiti: A Field Study of 107 Households. *Rural Remote Health* **2006**, *6* (3), 570.
(153) Duran Romero, D. A.; de Almeida Silva, M. C.; Chauque, B. J. M.; Benetti, A. D. Biosand Filter as a Point-of-Use Water Treatment Technology: Influence of Turbidity on Microorganism Removal Efficiency. *WATER* **2020**, *12* (8). https://doi.org/10.3390/w12082302.
(154) Elliott, M. A.; Stauber, C. E.; Koksal, F.; DiGiano, F. A.; Sobsey, M. D. Reductions of E. Coli, Echovirus Type 12 and Bacteriophages in an Intermittently Operated Household-Scale Slow Sand Filter. *Water Res.* **2008**, *42* (10–11), 2662–2670. https://doi.org/10.1016/j.watres.2008.01.016.
(155) Elliott, M. A.; DiGiano, F. A.; Sobsey, M. D. Virus Attenuation by Microbial Mechanisms during the Idle Time of a Household Slow Sand Filter. *Water Research* **2011**, *45* (14), 4092–4102. https://doi.org/10.1016/j.watres.2011.05.008.
(156) Elliott, M.; Stauber, C. E.; DiGiano, F. A.; de Aceituno, A. F.; Sobsey, M. D. Investigation of E. Coli and Virus Reductions Using Replicate, Bench-Scale Biosand Filter Columns and Two Filter Media. *Int J Environ Res Public Health* **2015**, *12* (9), 10276–10299. https://doi.org/10.3390/ijerph120910276.

(157) Fiore, M. M.; Minnings, K.; Fiore, L. D. Assessment of Biosand Filter Performance in Rural Communities in Southern Coastal Nicaragua: An Evaluation of 199 Households. *Rural Remote Health* **2010**, *10* (3), 1483.
(158) Frank, T. E.; Scheie, M. L.; Cachro, V.; Munoz, A. S. The Effect of Increasing Grain Size in Biosand Water Filters in Combination with Ultraviolet Disinfection. *J. Wate Sanit. Hyg. Dev.* **2014**, *4* (2), 206–213. https://doi.org/10.2166/washdev.2013.171.
(159) Ghebremichael, K.; Wasala, L. D.; Kennedy, M.; Graham, N. J. D. Comparative Treatment Performance and Hydraulic Characteristics of Pumice and Sand Biofilters for Point-of-Use Water Treatment. *J. Water Supply Res Technol.-Aqua* **2012**, *61* (4), 201–209. https://doi.org/10.2166/aqua.2012.100.
(160) Guan, P.; Prasher, S. O.; Afzal, M. T.; George, S.; Ronholm, J.; Dhiman, J.; Patel, R. M. Removal of Escherichia Coli from Lake Water in a Biochar-Amended Biosand Filtering System. *Ecological Engineering* **2020**, *150*. https://doi.org/10.1016/j.ecoleng.2020.105819.
(161) Hussain, G.; Haydar, S.; Bari, A. J.; Aziz, J. A.; Anis, M.; Asif, Z. Evaluation of Plastic Household Biosand Filter (BSF) In Combination with Solar Disinfection (SODIS) For Water Treatment. *J. Chem. Soc. Pak.* **2015**, *37* (2), 352–362.
(162) Jenkins, M. W.; Tiwari, S. K.; Darby, J. Bacterial, Viral and Turbidity Removal by Intermittent Slow Sand Filtration for Household Use in Developing Countries: Experimental Investigation and Modeling. *Water Research* **2011**, *45* (18), 6227–6239. https://doi.org/10.1016/j.watres.2011.09.022.
(163) Juarez, H.; Carrasco, M.; Vega, V.; Gomez, J.; Waarnars, M.; Prain, G. Water and Health at the Household Level in Eastern Lima, Peru: An Urban Ecosystem Approach. In *Sustainable City V: Urban Regeneration and Sustainability*; Gospodini, A., Brebbia, C. A., Eds.; Wit Press/Computational Mechanics Publications: Southampton, 2009; Vol. 117, pp 567–575.
(164) Kabir, A. H. M. E.; Sekine, M.; Ghosh, G. C. Towards Safely Managed Drinking Water Supply in the Hard-to-Reach Areas in Bangladesh: Modified Biosand Filters and Safe Storage. *DESALINATION AND WATER TREATMENT* **2020**, *173*, 177–185. https://doi.org/10.5004/dwt.2020.24815.
(165) Kang, J.-K.; Lee, C.-G.; Park, J.-A.; Kim, S.-B.; Choi, N.-C.; Park, S.-J. Adhesion of Bacteria to Pyrophyllite Clay in Aqueous Solution. *Environ. Technol.* **2013**, *34* (6), 703–710. https://doi.org/10.1080/09593330.2012.715677.
(166) Kennedy, T. J.; Hernandez, E. A.; Morse, A. N.; Anderson, T. A. Hydraulic Loading Rate Effect on Removal Rates in a BioSand Filter: A Pilot Study of Three Conditions. *Water, air and soil pollution.* **2012**, *223* (7), 4527–4537.
(167) Kennedy, T. J.; Anderson, T. A.; Hernandez, E. A.; Morse, A. N. Determining the Operational Limits of the Biosand Filter. *Water Sci. Technol.-Water Supply* **2013**, *13* (1), 56–65. https://doi.org/10.2166/ws.2012.075.
(168) Kim, S.; Bradshaw, R.; Kulkarni, P.; Allard, S.; Chiu, P. C.; Sapkota, A. R.; Newell, M. J.; Handy, E. T.; East, C. L.; Kniel, K. E.; Sharma, M. Zero-Valent Iron-Sand Filtration Reduces Escherichia Coli in Surface Water and Leafy Green Growing Environments. *Frontiers in Sustainable Food Systems* **2020**, *4*. https://doi.org/10.3389/fsufs.2020.00112.
(169) Lackey, L.; Semmendinger, K.; MacCarthy, M. Biological Sand Filter Performance Test Using Multiple Methods for Pathogen Detection: A Longitudinal Field Study in Kenya. *Water Air Soil Pollut* **2019**, *230* (7), 165. https://doi.org/10.1007/s11270-019-4218-6.

(170) Lynn, T. J.; Wanjugi, P.; Harwood, V. J.; Ergas, S. J. Dynamic Performance of Biosand Filters. *Journal - American Water Works Association* **2013**, *105* (10). https://doi.org/10.5942/jawwa.2013.105.0116.
(171) Maciel, P. M. F.; Sabogal-Paz, L. P. Household Slow Sand Filters with and without Water Level Control: Continuous and Intermittent Flow Efficiencies. *Environ Technol* **2020**, *41* (8), 944–958. https://doi.org/10.1080/09593330.2018.1515988.
(172) Mahmood, Q.; Baig, S. A.; Nawab, B.; Shafqat, M. N.; Pervez, A.; Zeb, B. S. Development of Low Cost Household Drinking Water Treatment System for the Earthquake Affected Communities in Northern Pakistan. *Desalination.* **2011**, *273* (2–3), 316–320.
(173) McKenzie, E. R.; Jenkins, M. W.; Tiwari, S.-S. K.; Darby, J.; Saenyi, W.; Gichaba, C. M. In-Home Performance and Variability of Biosand Filters Treating Turbid Surface and Rain Water in Rural Kenya. *J. Wate Sanit. Hyg. Dev.* **2013**, *3* (2), 189–198. https://doi.org/10.2166/washdev.2013.050.
(174) Medeiros, R. C.; de M. N. Fava, N.; Freitas, B. L. S.; Sabogal-Paz, L. P.; Hoffmann, M. T.; Davis, J.; Fernandez-Ibañez, P.; Byrne, J. A. Drinking Water Treatment by Multistage Filtration on a Household Scale: Efficiency and Challenges. *Water Research* **2020**, *178*. https://doi.org/10.1016/j.watres.2020.115816.
(175) Moropeng, R.; Budeli, P.; Mpenyana-Monyatsi, L.; Momba, M. Dramatic Reduction in Diarrhoeal Diseases through Implementation of Cost-Effective Household Drinking Water Treatment Systems in Makwane Village, Limpopo Province, South Africa. *IJERPH* **2018**, *15* (3), 410. https://doi.org/10.3390/ijerph15030410.
(176) Murphy, H. M.; McBean, E. A.; Farahbakhsh, K. Nitrification, Denitrification and Ammonification in Point-of-Use Biosand Filters in Rural Cambodia. *J. Water Health* **2010**, *8* (4), 803–817. https://doi.org/10.2166/wh.2010.163.
(177) Mutemi, S.; Hoko, Z.; Makurira, H. Investigating Feasibility of Use of Bio-Sand Filters for Household Water Treatment in Epworth, Zimbabwe. *Physics and Chemistry of the Earth* **2020**, *117*. https://doi.org/10.1016/j.pce.2020.102864.
(178) Nair, A. T.; Ahammed, M. M.; Davra, K. Influence of Operating Parameters on the Performance of a Household Slow Sand Filter. *Water Sci. Technol.-Water Supply* **2014**, *14* (4), 643–649. https://doi.org/10.2166/ws.2014.021.
(179) Napotnik, J. A.; Baker, D.; Jellison, K. L. Influence of Sand Depth and Pause Period on Microbial Removal in Traditional and Modified Biosand Filters. *Water Research* **2021**, *189*. https://doi.org/10.1016/j.watres.2020.116577.
(180) Nasser Fava, N. de M.; Terin, U. C.; Freitas, B. L. S.; Sabogal-Paz, L. P.; Fernandez-Ibañez, P.; Anthony Byrne, J. Household Slow Sand Filters in Continuous and Intermittent Flows and Their Efficiency in Microorganism's Removal from River Water. *Environmental Technology* **2020**, 1–10. https://doi.org/10.1080/09593330.2020.1841834.
(181) Ngwenya, B. T.; Curry, P.; Kapetas, L. Transport and Viability of Escherichia Coli Cells in Clean and Iron Oxide Coated Sand Following Coating with Silver Nanoparticles. *J. Contam. Hydrol.* **2015**, *179*, 35–46. https://doi.org/10.1016/j.jconhyd.2015.05.005.
(182) Pompei, C. M. E.; Ciric, L.; Canales, M.; Karu, K.; Vieira, E. M.; Campos, L. C. Influence of PPCPs on the Performance of Intermittently Operated Slow Sand Filters for Household Water Purification. *Sci. Total Environ.* **2017**, *581–582*, 174–185. https://doi.org/10.1016/j.scitotenv.2016.12.091.

(183) Rao, S. M.; Malini, R.; Lydia, A.; Lee, Y. Contaminants Removal by Bentonite Amended Slow Sand Filter. *J. Water. Chem. Technol.* **2013**, *35* (1), 23–29. https://doi.org/10.3103/S1063455X13010049.
(184) Sabogal-Paz, L. P.; Campos, L. C.; Bogush, A.; Canales, M. Household Slow Sand Filters in Intermittent and Continuous Flows to Treat Water Containing Low Mineral Ion Concentrations and Bisphenol A. *SCIENCE OF THE TOTAL ENVIRONMENT* **2020**, *702*. https://doi.org/10.1016/j.scitotenv.2019.135078.
(185) Singer, S.; Skinner, B.; Cantwell, R. E. Impact of Surface Maintenance on BioSand Filter Performance and Flow. *J Water Health* **2017**, *15* (2), 262–272. https://doi.org/10.2166/wh.2017.129.
(186) Sisson, A. J.; Wampler, P. J.; Rediske, R. R.; McNair, J. N.; Frobish, D. J. Long-Term Field Performance of Biosand Filters in the Artibonite Valley, Haiti. *Am. J. Trop. Med. Hyg.* **2013**, *88* (5), 862–867. https://doi.org/10.4269/ajtmh.12-0345.
(187) Stauber, C. E.; Kominek, B.; Liang, K. R.; Osman, M. K.; Sobsey, M. D. Evaluation of the Impact of the Plastic BioSand Filter on Health and Drinking Water Quality in Rural Tamale, Ghana. *Int J Environ Res Public Health* **2012**, *9* (11), 3806–3823. https://doi.org/10.3390/ijerph9113806.
(188) Stauber, C. E.; Elliott, M. A.; Koksal, F.; Ortiz, G. M.; DiGiano, F. A.; Sobsey, M. D. Characterisation of the Biosand Filter for E. Coli Reductions from Household Drinking Water under Controlled Laboratory and Field Use Conditions. *Water Sci. Technol.* **2006**, *54* (3), 1–7. https://doi.org/10.2166/wst.2006.440.
(189) Tellen, V.; Nkeng, G.; Dentel, S. Improved Filtration Technology for Pathogen Reduction in Rural Water Supplies. *Water* **2010**, *2* (2), 285–306. https://doi.org/10.3390/w2020285.
(190) Terin, U. C.; Sabogal-Paz, L. P. Microcystis Aeruginosa and Microcystin-LR Removal by Household Slow Sand Filters Operating in Continuous and Intermittent Flows. *Water Res* **2019**, *150*, 29–39. https://doi.org/10.1016/j.watres.2018.11.055.
(191) Tundia, K. R.; Ahammed, M. M.; George, D. The Effect of Operating Parameters on the Performance of a Biosand Filter: A Statistical Experiment Design Approach. *Water Sci. Technol.-Water Supply* **2016**, *16* (3), 775–782. https://doi.org/10.2166/ws.2015.191.
(192) Vanderzwaag, J. C.; Atwater, J. W.; Bartlett, K. H.; Baker, D. Field Evaluation of Long-Term Performance and Use of Biosand Filters in Posoltega, Nicaragua. *Water Qual. Res. J. Canada* **2009**, *44* (2), 111–121.
(193) Wang, H.; Narihiro, T.; Straub, A. P.; Pugh, C. R.; Tamaki, H.; Moor, J. F.; Bradley, I. M.; Kamagata, Y.; Liu, W.-T.; Nguyen, T. H. MS2 Bacteriophage Reduction and Microbial Communities in Biosand Filters. *Environ. Sci. Technol.* **2014**, *48* (12), 6702–6709. https://doi.org/10.1021/es500494s.
(194) Wegelin, M.; Schertenleib, R.; Boller, M. Decade of Roughing Filters. Development of a Rural Water-Treatment Process for Developing Countries. *Aqua- Journal of Water Supply: Research and Technology* **1991**, *40* (5), 304–316.
(195) Yildiz, B. S. Performance Assessment of Modified Biosand Filter with an Extra Disinfection Layer. *J. Water Supply Res Technol.-Aqua* **2016**, *65* (3), 266–276. https://doi.org/10.2166/aqua.2016.103.
(196) Young-Rojanschi, C.; Madramootoo, C. Intermittent versus Continuous Operation of Biosand Filters. *Water Res.* **2014**, *49*, 1–10. https://doi.org/10.1016/j.watres.2013.11.011.

(197) Young-Rojanschi, C.; Madramootoo, C. Comparing the Performance of Biosand Filters Operated with Multiday Residence Periods. *J. Water Supply Res Technol.-Aqua* **2015**, *64* (2), 157–167. https://doi.org/10.2166/aqua.2014.027.
(198) Brown, J.; Sobsey, M. D. Boiling as Household Water Treatment in Cambodia: A Longitudinal Study of Boiling Practice and Microbiological Effectiveness. *Am. J. Trop. Med. Hyg.* **2012**, *87* (3), 394–398. https://doi.org/10.4269/ajtmh.2012.11-0715.
(199) Christen, A.; Navarro, C. M.; Mäusezahl, D. Safe Drinking Water and Clean Air: An Experimental Study Evaluating the Concept of Combining Household Water Treatment and Indoor Air Improvement Using the Water Disinfection Stove (WADIS). *Int J Hyg Environ Health* **2009**, *212* (5), 562–568. https://doi.org/10.1016/j.ijheh.2009.01.001.
(200) Clasen, T. F.; Thao, D. H.; Boisson, S.; Shipin, O. Microbiological Effectiveness and Cost of Boiling to Disinfect Drinking Water in Rural Vietnam. *Environ. Sci. Technol.* **2008**, *42* (12), 4255–4260.
(201) Clasen, T.; McLaughlin, C.; Nayaar, N.; Boisson, S.; Gupta, R.; Desai, D.; Shah, N. Microbiological Effectiveness and Cost of Disinfecting Water by Boiling in Semi-Urban India. *Am. J. Trop. Med. Hyg.* **2008**, *79* (3), 407–413.
(202) Gupta, S. K.; Islam, M. S.; Johnston, R.; Ram, P. K.; Luby, S. P. The Chulli Water Purifier: Acceptability and Effectiveness of an Innovative Strategy for Household Water Treatment in Bangladesh. *Am. J. Trop. Med. Hyg.* **2008**, *78* (6), 979–984.
(203) Islam, M. F.; Johnston, R. B. Household Pasteurization of Drinking-Water: The Chulli Water-Treatment System. *J. Heatlh Popul. Nutr.* **2006**, *24* (3), 356–362.
(204) Liu, Y.; Kumblathan, T.; Uppal, G. K.; Zhou, A.; Moe, B.; Hrudey, S. E.; Li, X.-F. A Hidden Risk: Survival and Resuscitation of Escherichia Coli O157:H7 in the Viable but Nonculturable State after Boiling or Microwaving. *Water Research* **2020**, *183*. https://doi.org/10.1016/j.watres.2020.116102.
(205) McGuigan, K. G.; Joyce, T. M.; Conroy, R. M.; Gillespie, J. B.; Elmore-Meegan, M. Solar Disinfection of Drinking Water Contained in Transparent Plastic Bottles: Characterizing the Bacterial Inactivation Process. *J. Appl. Microbiol.* **1998**, *84* (6), 1138–1148.
(206) Psutka, R.; Peletz, R.; Michelo, S.; Kelly, P.; Clasen, T. Assessing the Microbiological Performance and Potential Cost of Boiling Drinking Water in Urban Zambia. *Environmental science & technology* **2011**, *45* (14), 6095–6101. https://doi.org/10.1021/es2004045.
(207) Rosa, G.; Miller, L.; Clasen, T. Microbiological Effectiveness of Disinfecting Water by Boiling in Rural Guatemala. *Am. J. Trop. Med. Hyg.* **2010**, *82* (3), 473–477. https://doi.org/10.4269/ajtmh.2010.09-0320.
(208) Sodha, S. V.; Menon, M.; Trivedi, K.; Ati, A.; Figueroa, M. E.; Ainslie, R.; Wannemuehler, K.; Quick, R. Microbiologic Effectiveness of Boiling and Safe Water Storage in South Sulawesi, Indonesia. *Journal of water and health* **2011**, *9* (3), 577–585. https://doi.org/10.2166/wh.2011.255.
(209) Theitler, D. J.; Nasser, A.; Gerchman, Y.; Kribus, A.; Mamane, H. Synergistic Effect of Heat and Solar UV on DNA Damage and Water Disinfection of E. Coli and Bacteriophage MS2. *Journal of Water and Health* **2012**, *10* (4), 605–618. https://doi.org/10.2166/wh.2012.072.
(210) Ahammed, M. M.; Dave, S. Effect of Source Water Quality on Solar Disinfection Rate under Multiple Experimental Conditions. *Journal of Water Sanitation and Hygiene for Development* **2014**, *4* (4), 714–719. https://doi.org/10.2166/washdev.2014.120.

(211) Akintola, O. A.; Sangodoyin, A. Y. Design, Development, and Performance Evaluation of Solar Heating System for Disinfection of Domestic Roof-Harvested Rainwater. *Int Sch Res Notices* **2015**, *2015*, 529527. https://doi.org/10.1155/2015/529527.
(212) Ali, S. I.; MacDonald, M.; Jincy, J.; Sampath, K. A.; Vinothini, G.; Philip, L.; Hall, K.; Aronson, K. Efficacy of an Appropriate Point-of-Use Water Treatment Intervention for Low-Income Communities in India Utilizing Moringa Oleifera, Sari-Cloth Filtration and Solar UV Disinfection. *Journal of Water Sanitation and Hygiene for Development* **2011**, *1* (2), 112–123. https://doi.org/10.2166/washdev.2011.043.
(213) Alotaibi, M. A.; Heaselgrave, W. Solar Disinfection of Water for Inactivation of Enteric Viruses and Its Enhancement by Riboflavin. *Food and Environmental Virology* **2011**, *3* (2), 70–73. https://doi.org/10.1007/s12560-011-9058-5.
(214) Alrousan, D. M. A.; Polo-Lopez, M. I.; Dunlop, P. S. M.; Fernandez-Ibanez, P.; Byrne, J. A. Solar Photocatalytic Disinfection of Water with Immobilised Titanium Dioxide in Re-Circulating Flow CPC Reactors. *Appl. Catal. B-Environ.* **2012**, *128*, 126–134. https://doi.org/10.1016/j.apcatb.2012.07.038.
(215) Amin, M. T.; Han, M. Y. Roof-Harvested Rainwater for Potable Purposes: Application of Solar Collector Disinfection (SOCO-DIS). *Water Res.* **2009**, *43* (20), 5225–5235. https://doi.org/10.1016/j.watres.2009.08.041.
(216) Amin, M. T.; Alazba, A. A.; Amin, M. N.; Han, M. Y. Cost-Effective and Sustainable Solutions to Enhance the Solar Disinfection Efficiency Improving the Microbiological Quality of Rooftop-Harvested Rainwater. *Desalin. Water Treat.* **2014**, *52* (28–30), 5252–5263. https://doi.org/10.1080/19443994.2013.808591.
(217) Asiimwe, J. K.; Quilty, B.; Muyanja, C. K.; McGuigan, K. G. Field Comparison of Solar Water Disinfection (SODIS) Efficacy between Glass and Polyethylene Terephalate (PET) Plastic Bottles under Sub-Saharan Weather Conditions. *J Water Health* **2013**, *11* (4), 729–737. https://doi.org/10.2166/wh.2013.197.
(218) Berney, M.; Weilenmann, H.-U.; Egli, T. Flow-Cytometric Study of Vital Cellular Functions in Escherichia Coli during Solar Disinfection (SODIS). *Microbiology (Reading, Engl.)* **2006**, *152* (Pt 6), 1719–1729. https://doi.org/10.1099/mic.0.28617-0.
(219) Bigoni, R.; Kötzsch, S.; Sorlini, S.; Egli, T. Solar Water Disinfection by a Parabolic Trough Concentrator (PTC): Flow-Cytometric Analysis of Bacterial Inactivation. *Journal of Cleaner Production* **2014**, *67*, 62–71. https://doi.org/10.1016/j.jclepro.2013.12.014.
(220) Bosshard, F.; Armand, F.; Hamelin, R.; Kohn, T. Mechanisms of Human Adenovirus Inactivation by Sunlight and UVC Light as Examined by Quantitative PCR and Quantitative Proteomics. *Applied and environmental microbiology* **2013**, *79* (4), 1325–1332. https://doi.org/10.1128/aem.03457-12.
(221) Bosshard, F.; Berney, M.; Scheifele, M.; Weilenmann, H. U.; Egli, T. Solar Disinfection (SODIS) and Subsequent Dark Storage of Salmonella Typhimurium and Shigella Flexneri Monitored by Flow Cytometry. *Microbiology (Reading, England)* **2009**, *155* (Pt 4), 1310–1317. https://doi.org/10.1099/mic.0.024794-0.
(222) Boyle, M.; Sichel, C.; Fernández-Ibáñez, P.; Arias-Quiroz, G. B.; Iriarte-Puña, M.; Mercado, A.; Ubomba-Jaswa, E.; McGuigan, K. G. Bactericidal Effect of Solar Water Disinfection under Real Sunlight Conditions. *Applied and environmental microbiology* **2008**, *74* (10), 2997–3001. https://doi.org/10.1128/aem.02415-07.

(223) Carielo, G.; Calazans, G.; Lima, G.; Tiba, C. Solar Water Pasteurizer: Productivity and Treatment Efficiency in Microbial Decontamination. *Renew. Energy* **2017**, *105*, 257–269. https://doi.org/10.1016/j.renene.2016.12.042.
(224) Castro-Alferez, M.; Inmaculada Polo-Lopez, M.; Fernandez-Ibanez, P. Intracellular Mechanisms of Solar Water Disinfection. *Sci Rep* **2016**, *6*, 38145. https://doi.org/10.1038/srep38145.
(225) Castro-Alférez, M.; Inmaculada Polo-López, M.; Marugán, J.; Fernández-Ibáñez, P. Validation of a Solar-Thermal Water Disinfection Model for Escherichia Coli Inactivation in Pilot Scale Solar Reactors and Real Conditions. *Chemical Engineering Journal* **2018**, *331*, 831–840. https://doi.org/10.1016/j.cej.2017.09.015.
(226) Chaudhari, R.; Ahammed, M. M.; Dave, S. Solar Disinfection of Natural Waters with Modified Solar Concentrators. *Water Sci. Technol.-Water Supply* **2013**, *13* (2), 462–468. https://doi.org/10.2166/ws.2013.042.
(227) Clements, T.; Reyneke, B.; Strauss, A.; Khan, W. Persistence of Viable Bacteria in Solar Pasteurised Harvested Rainwater. *Water Air Soil Pollut* **2019**, *230* (6), 130. https://doi.org/10.1007/s11270-019-4184-z.
(228) Dejung, S.; Fuentes, I.; Almanza, G.; Jarro, R.; Navarro, L.; Arias, G.; Urquieta, E.; Torrico, A.; Fenandez, W.; Iriarte, M.; Birrer, C.; Stahel, W. A.; Wegelin, M. Effect of Solar Water Disinfection (SODIS) on Model Microorganisms under Improved and Field SODIS Conditions. *Journal of Water Supply: Research and Technology-Aqua* **2007**, *56* (4), 245–256. https://doi.org/10.2166/aqua.2007.058.
(229) Dessie, A.; Alemayehu, E.; Mekonen, S.; Legesse, W.; Kloos, H.; Ambelu, A. Solar Disinfection: An Approach for Low-Cost Household Water Treatment Technology in Southwestern Ethiopia. *J Environ Health Sci Eng* **2014**, *12* (1), 25. https://doi.org/10.1186/2052-336X-12-25.
(230) Figueredo-Fernandez, M.; Gutierrez-Alfaro, S.; Acevedo-Merino, A.; Manzano, M. A. Estimating Lethal Dose of Solar Radiation for Enterococcus Inactivation through Radiation Reaching the Water Layer. Application to Solar Water Disinfection (SODIS). *Sol. Energy* **2017**, *158*, 303–310. https://doi.org/10.1016/j.solener.2017.09.006.
(231) Fisher, M. B.; Iriarte, M.; Nelson, K. L. Solar Water Disinfection (SODIS) of Escherichia Coli, Enterococcus Spp., and MS2 Coliphage: Effects of Additives and Alternative Container Materials. *Water Res.* **2012**, *46* (6), 1745–1754. https://doi.org/10.1016/j.watres.2011.12.048.
(232) Fontan-Sainz, M.; Gomez-Couso, H.; Fernandez-Ibanez, P.; Ares-Mazas, E. Evaluation of the Solar Water Disinfection Process (SODIS) Against Cryptosporidium Parvum Using a 25-L Static Solar Reactor Fitted with a Compound Parabolic Collector (CPC). *Am. J. Trop. Med. Hyg.* **2012**, *86* (2), 223–228. https://doi.org/10.4269/ajtmh.2012.11-0325.
(233) Fujioka, R. S.; Yoneyama, B. S. Sunlight Inactivation of Human Enteric Viruses and Fecal Bacteria. *Water science and technology : a journal of the International Association on Water Pollution Research* **2002**, *46* (11–12), 291–295.
(234) Gomez-Couso, H.; Fontan-Sainz, M.; Fernandez-Ibanez, P.; Ares-Mazas, E. Speeding up the Solar Water Disinfection Process (SODIS) against Cryptosporidium Parvum by Using 2.5 l Static Solar Reactors Fitted with Compound Parabolic Concentrators (CPCs). *Acta Trop.* **2012**, *124* (3), 235–242. https://doi.org/10.1016/j.actatropica.2012.08.018.
(235) Gomez-Couso, H.; Fontan-Sainz, M.; Navntoft, C.; Fernandez-Ibanez, P.; Ares-Mazas, E. Comparison of Different Solar Reactors for Household Disinfection of Drinking Water in

Developing Countries: Evaluation of Their Efficacy in Relation to the Waterborne Enteropathogen Cryptosporidium Parvum. *Trans. Roy. Soc. Trop. Med. Hyg.* **2012**, *106* (11), 645–652. https://doi.org/10.1016/j.trstmh.2012.07.014.
(236) Gutierrez-Alfaro, S.; Acevedo, A.; Figueredo, M.; Saladin, M.; Manzano, M. A. Accelerating the Process of Solar Disinfection (SODIS) by Using Polymer Bags. *J. Chem. Technol. Biotechnol.* **2017**, *92* (2), 298–304. https://doi.org/10.1002/j.ctb.5005.
(237) Haider, H.; Ali, W.; Haydar, S.; Tesfamariam, S.; Sadiq, R. Modeling Exposure Period for Solar Disinfection (SODIS) under Varying Turbidity and Cloud Cover Conditions. *Clean Technol. Environ. Policy* **2014**, *16* (5), 861–874. https://doi.org/10.1007/s10098-013-0677-4.
(238) Harding, A. S.; Schwab, K. J. Using Limes and Synthetic Psoralens to Enhance Solar Disinfection of Water (SODIS): A Laboratory Evaluation with Norovirus, Escherichia Coli, and MS2. *Am. J. Trop. Med. Hyg.* **2012**, *86* (4), 566–572. https://doi.org/10.4269/ajtmh.2012.11-0370.
(239) Heaselgrave, W.; Kilvington, S. The Efficacy of Simulate d Solar Disinfection (SODIS) against Coxsackievirus, Poliovirus and Hepatitis A Virus. *Journal of Water and Health* **2012**, *10* (4), 531–538. https://doi.org/10.2166/wh.2012.128.
(240) Helali, S.; Inmaculada Polo-Lopez, M.; Fernandez-Ibanez, P.; Ohtani, B.; Amano, F.; Malato, S.; Guillard, C. Solar Photocatalysis: A Green Technology for E. Coli Contaminated Water Disinfection. Effect of Concentration and Different Types of, Suspended Catalyst. *J. Photochem. Photobiol. A-Chem.* **2014**, *276*, 31–40. https://doi.org/10.1016/j.jphotochem.2013.11.011.
(241) Hindiyeh, M.; Ali, A. Investigating the Efficiency of Solar Energy System for Drinking Water Disinfection. *Desalination* **2010**, *259* (1–3), 208–215. https://doi.org/10.1016/j.desal.2010.04.004.
(242) Islam, M. A.; Azad, A. K.; Akber, M. A.; Rahman, M.; Sadhu, I. Effectiveness of Solar Disinfection (SODIS) in Rural Coastal Bangladesh. *J. Water Health* **2015**, *13* (4), 1113–1122. https://doi.org/10.2166/wh.2015.186.
(243) Joyce, T. M.; McGuigan, K. G.; Elmore-Meegan, M.; Conroy, R. M. Inactivation of Fecal Bacteria in Drinking Water by Solar Heating. *Applied and environmental microbiology* **1996**, *62* (2), 399–402.
(244) Kehoe, S. C.; Joyce, T. M.; Ibrahim, P.; Gillespie, J. B.; Shahar, R. A.; McGuigan, K. G. Effect of Agitation, Turbidity, Aluminium Foil Reflectors and Container Volume on the Inactivation Efficiency of Batch-Process Solar Disinfectors. *Water Research* **2001**, *35* (4), 1061–1065. https://doi.org/10.1016/S0043-1354(00)00353-5.
(245) Keogh, M. B.; Castro-Alférez, M.; Polo-López, M. I.; Fernández Calderero, I.; Al-Eryani, Y. A.; Joseph-Titus, C.; Sawant, B.; Dhodapkar, R.; Mathur, C.; McGuigan, K. G.; Fernández-Ibáñez, P. Capability of 19-L Polycarbonate Plastic Water Cooler Containers for Efficient Solar Water Disinfection (SODIS): Field Case Studies in India, Bahrain and Spain. *Solar Energy* **2015**, *116*, 1–11. https://doi.org/10.1016/j.solener.2015.03.035.
(246) King, B. J.; Hoefel, D.; Daminato, D. P.; Fanok, S.; Monis, P. T. Solar UV Reduces Cryptosporidium Parvum Oocyst Infectivity in Environmental Waters. *J. Appl. Microbiol.* **2008**, *104* (5), 1311–1323. https://doi.org/10.1111/j.1365-2672.2007.03658.x.
(247) Kruti, J.; Shilpa, D. Solar Water Disinfection under Real Conditions with or Solar Collector. *J. Environ. Res. Develop* **2012**, *7* (2), 1085–1089.

(248) Lamore, Y.; Beyene, A.; Megersa, M.; Fekadu, S. Solar Disinfection Potentials of Aqua Lens, Photovoltaic and Glass Bottle Subsequent to Plant-Based Coagulant: For Low-Cost Household Water Treatment Systems. *Applied water science* **2018**, *8* (4), 100–100. https://doi.org/10.1007/s13201-018-0739-1.
(249) Mani, S. K.; Kanjur, R.; Bright Singh, I. S.; Reed, R. H. Comparative Effectiveness of Solar Disinfection Using Small-Scale Batch Reactors with Reflective, Absorptive and Transmissive Rear Surfaces. *Water Res.* **2006**, *40* (4), 721–727. https://doi.org/10.1016/j.watres.2005.11.039.
(250) Mansoor Ahammed, M.; Dave, S.; Nair, A. T. Effect of Water Quality Parameters on Solar Water Disinfection: A Statistical Experiment Design Approach. *Desalination and Water Treatment* **2015**, *56* (2), 315–326. https://doi.org/10.1080/19443994.2014.940398.
(251) Martinez-Garcia, A.; Vincent, M.; Rubiolo, V.; Domingos, M.; Canela, M. C.; Oller, I.; Fernandez-Ibanez, P.; Inmaculada Polo-Lopez, M. Assessment of a Pilot Solar V-Trough Reactor for Solar Water Disinfection. *CHEMICAL ENGINEERING JOURNAL* **2020**, *399*. https://doi.org/10.1016/j.cej.2020.125719.
(252) Mbonimpa, E. G.; Vadheim, B.; Blatchley, E. R. Continuous-Flow Solar UVB Disinfection Reactor for Drinking Water. *Water Res.* **2012**, *46* (7), 2344–2354. https://doi.org/10.1016/j.watres.2012.02.003.
(253) McGuigan, K. G.; Méndez-Hermida, F.; Castro-Hermida, J. A.; Ares-Mazás, E.; Kehoe, S. C.; Boyle, M.; Sichel, C.; Fernández-Ibáñez, P.; Meyer, B. P.; Ramalingham, S.; Meyer, E. A. Batch Solar Disinfection Inactivates Oocysts of Cryptosporidium Parvum and Cysts of Giardia Muris in Drinking Water. *J. Appl. Microbiol.* **2006**, *101* (2), 453–463. https://doi.org/10.1111/j.1365-2672.2006.02935.x.
(254) Meera, V.; Ahammed, M. M. Solar Disinfection for Household Treatment of Roof-Harvested Rainwater. *Water Science and Technology: Water Supply* **2008**, *8* (2), 153–160. https://doi.org/10.2166/ws.2008.054.
(255) Mendez-Hermida, F.; Ares-Mazas, E.; McGuigan, K. G.; Boyle, M.; Sichel, C.; Fernandez-Ibanez, P. Disinfection of Drinking Water Contaminated with Cryptosporidium Parvum Oocysts under Natural Sunlight and Using the Photocatalyst TiO2. *J. Photochem. Photobiol. B-Biol.* **2007**, *88* (2–3), 105–111. https://doi.org/10.1016/j.jphotobiol.2007.05.004.
(256) Meyer, V.; Reed, R. SOLAIR Disinfection of Coliform Bacteria in Hand-Drawn Drinking Water. *J Water SA* **2001**, *27* (1), 49–52.
(257) Mtapuri-Zinyowera, S.; Midzi, N.; Muchaneta-Kubara, C. E.; Simbini, T.; Mduluza, T. Impact of Solar Radiation in Disinfecting Drinking Water Contaminated with Giardia Duodenalis and Entamoeba Histolytica/Dispar at a Point-of-Use Water Treatment. *J. Appl. Microbiol.* **2009**, *106* (3), 847–852. https://doi.org/10.1111/j.1365-2672.2008.04054.x.
(258) Mustafa, A.; Scholz, M.; Khan, S.; Ghaffar, A. Application of Solar Disinfection for Treatment of Contaminated Public Water Supply in a Developing Country: Field Observations. *Journal of Water and Health* **2013**, *11* (1), 135–145. https://doi.org/10.2166/wh.2012.119.
(259) Nalwanga, R.; Quilty, B.; Muyanja, C.; Fernandez-Ibanez, P.; McGuigan, K. G. Evaluation of Solar Disinfection of E. Coli under Sub-Saharan Field Conditions Using a 25L Borosilicate Glass Batch Reactor Fitted with a Compound Parabolic Collector. *Sol. Energy* **2014**, *100*, 195–202. https://doi.org/10.1016/j.solener.2013.12.011.

(260) Ndounla, J.; Pulgarin, C. Solar Light (Hv) and H2O2/Hv Photo-Disinfection of Natural Alkaline Water (PH 8.6) in a Compound Parabolic Collector at Different Day Periods in Sahelian Region. *Environmental Science and Pollution Research* **2015**, *22* (21), 17082–17094. https://doi.org/10.1007/s11356-015-4784-0.
(261) Nwankwo, E. J.; Agunwamba, J. C.; Nnaji, C. C. Effect of Radiation Intensity, Water Temperature and Support-Base Materials on the Inactivation Efficiency of Solar Water Disinfection (SODIS). *Water Resour Manage* **2019**, *33* (13), 4539–4551. https://doi.org/10.1007/s11269-019-02407-4.
(262) Oates, P. M.; Shanahan, P.; Polz, M. F. Solar Disinfection (SODIS): Simulation of Solar Radiation for Global Assessment and Application for Point-of-Use Water Treatment in Haiti. *Water Res.* **2003**, *37* (1), 47–54.
(263) Okurut, K.; Wozei, E.; Kulabako, R.; Nabasirye, L.; Kinobe, J. Calibrating an Optimal Condition Model for Solar Water Disinfection in Peri-Urban Household Water Treatment in Kampala, Uganda. *J Water Health* **2013**, *11* (1), 98–109. https://doi.org/10.2166/wh.2012.199.
(264) Ozores Diez, P.; Giannakis, S.; Rodríguez-Chueca, J.; Wang, D.; Quilty, B.; Devery, R.; McGuigan, K.; Pulgarin, C. Enhancing Solar Disinfection (SODIS) with the Photo-Fenton or the Fe2+/Peroxymonosulfate-Activation Process in Large-Scale Plastic Bottles Leads to Toxicologically Safe Drinking Water. *Water Research* **2020**, *186*. https://doi.org/10.1016/j.watres.2020.116387.
(265) Pham Diep N.; Le Minh-Vien; Hoang Hoang Anh; Tran Xuan T.T.; Nguyen Tuan-Anh. Fabrication of TiO2 Monolithic Photocatalyst and Evaluation of Its Antibacterial Activity under Simulated Solar Irradiation. *Chemical Engineering Transactions* **2020**, *78*, 355–360. https://doi.org/10.3303/CET2078060.
(266) Polo, D.; García-Fernández, I.; Fernández-Ibáñez, P.; Romalde, J. L. Solar Water Disinfection (SODIS): Impact on Hepatitis A Virus and on a Human Norovirus Surrogate under Natural Solar Conditions. *International microbiology : the official journal of the Spanish Society for Microbiology* **2015**, *18* (1), 41–49. https://doi.org/10.2436/20.1501.01.233.
(267) Polo-López, M. I.; Fernández-Ibáñez, P.; Ubomba-Jaswa, E.; Navntoft, C.; García-Fernández, I.; Dunlop, P. S. M.; Schmid, M.; Byrne, J. A.; McGuigan, K. G. Elimination of Water Pathogens with Solar Radiation Using an Automated Sequential Batch CPC Reactor. *J. Hazard. Mater.* **2011**, *196*, 16–21. https://doi.org/10.1016/j.jhazmat.2011.08.052.
(268) Polo-López, M. I.; Martínez-García, A.; Abeledo-Lameiro, M. J.; H, H. G.-C.; E, E. A.-M.; Reboredo-Fernández, A.; Morse, T. D.; Buck, L.; Lungu, K.; McGuigan, K. G.; Fernández-Ibáñez, P. Microbiological Evaluation of 5 L- and 20 L-Transparent Polypropylene Buckets for Solar Water Disinfection (SODIS). *Molecules (Basel, Switzerland)* **2019**, *24* (11). https://doi.org/10.3390/molecules24112193.
(269) Reddy Sajjala, S.; Al Dawery, S. K.; Ahmed, A.; Al jabri, M. A. N. Disinfection of Total Coliform Bacteria in Falaj Water by Solar Water Disinfection (SODIS). *Caspian Journal of Environmental Sciences* **2019**, *17* (4), 285–294. https://doi.org/10.22124/cjes.2019.3802.
(270) Reed, R. H. Solar Inactivation of Faecal Bacteria in Water: The Critical Role of Oxygen. *Lett. Appl. Microbiol.* **1997**, *24* (4), 276–280.

(271) Reed, R. H.; Mani, S. K.; Meyer, V. Solar Photo-Oxidative Disinfection of Drinking Water: Preliminary Field Observations. *Letters in applied microbiology* **2000**, *30* (6), 432–436. https://doi.org/10.1046/j.1472-765x.2000.00741.x.
(272) Reyneke, B.; Dobrowsky, P. H.; Ndlovu, T.; Khan, S.; Khan, W. EMA-QPCR to Monitor the Efficiency of a Closed-Coupled Solar Pasteurization System in Reducing Legionella Contamination of Roof-Harvested Rainwater. *Science of the Total Environment* **2016**, *553*, 662–670. https://doi.org/10.1016/j.scitotenv.2016.02.108.
(273) Rijal, G. K.; Fujioka, R. S. Synergistic Effect of Solar Radiation and Solar Heating to Disinfect Drinking Water Sources. *Water Sci. Technol.* **2001**, *43* (12), 155–162.
(274) Rommozzi, E.; Giannakis, S.; Giovannetti, R.; Vione, D.; Pulgarin, C. Detrimental vs. Beneficial Influence of Ions during Solar (SODIS) and Photo-Fenton Disinfection of E. Coli in Water: (Bi)Carbonate, Chloride, Nitrate and Nitrite Effects. *Applied Catalysis B: Environmental* **2020**, *270*. https://doi.org/10.1016/j.apcatb.2020.118877.
(275) Safapour, N.; Metcalf, R. H. Enhancement of Solar Water Pasteurization with Reflectors. *Applied and Environmental Microbiology* **1999**, *65* (2), 859–861.
(276) Saleemi, A. R.; Ramzan, N.; Hafeez, M. N. Investigating Catalytic Effect of Titanium Dioxide on Solar Disinfection of Water. *J. Chem. Soc. Pak.* **2009**, *31* (1), 26–30.
(277) Salih, F. M. Solar Water Disinfection and Possible Application for Household Purposes. *International Journal of Solar Energy* **2001**, *21* (4), 267–279.
(278) Sommer, B.; Mariño, A.; Solarte, Y.; Salas, M. L.; Dierolf, C.; Valiente, C.; Mora, D.; Rechsteiner, R.; Setter, P.; Wirojanagud, W.; Ajarmeh, H.; Al-Hassan, A.; Wegelin, M. SODIS - An Emerging Water Treatment Process. *Journal of Water Supply: Research and Technology - AQUA* **1997**, *46* (3), 127–137.
(279) Sreeja, S.; Shetty, V. K. Microbial Disinfection of Water with Endotoxin Degradation by Photocatalysis Using Ag@TiO2 Core Shell Nanoparticles. *Environ. Sci. Pollut. Res.* **2016**, *23* (18), 18154–18164. https://doi.org/10.1007/s11356-016-6841-8.
(280) Ubomba-Jaswa, E.; Fernández-Ibáñez, P.; Navntoft, C.; Polo-López, M. I.; McGuigan, K. G. Investigating the Microbial Inactivation Efficiency of a 25 L Batch Solar Disinfection (SODIS) Reactor Enhanced with a Compound Parabolic Collector (CPC) for Household Use. *Journal of chemical technology and biotechnology.* **2010**, *85* (8), 1028–1037.
(281) Villen, L.; Manjon, F.; Garcia-Fresnadillo, D.; Orellana, G. Solar Water Disinfection by Photocatalytic Singlet Oxygen Production in Heterogeneous Medium. *Appl. Catal. B-Environ.* **2006**, *69* (1–2), 1–9. https://doi.org/10.1016/j.apcatb.2006.05.015.
(282) Vivar, M.; Fuentes, M.; Pichel, N.; López-Vargas, A.; Rodrigo, M. J.; Srithar, K. Photovoltaic and Solar Disinfection Technology Meeting the Needs of Water and Electricity of a Typical Household in Developing Countries: From a Solar Home System to a Full-Functional Hybrid System. *Science of the Total Environment* **2020**, *747*. https://doi.org/10.1016/j.scitotenv.2020.141082.
(283) Wegelin, M., S., B. Solar Water Disinfection (SODIS)- Destined for Worldwide Use? *Waterlines* **1998**, *16* (3), 30–32.
(284) Wegelin, M.; Canonica, S.; Mechsner, K.; Fleischmann, T.; Pesaro, F.; Metzler, A. Solar Water Disinfection: Scope of the Process and Analysis of Radiation Experiments. *J Aqua* **1994**, *43* (4), 154–169.
(285) Younas, H.; Qazi, I. A.; Hashmi, I.; Ali Awan, M.; Mahmood, A.; Qayyum, H. A. Visible Light Photocatalytic Water Disinfection and Its Kinetics Using Ag-Doped Titania

Nanoparticles. *Environmental Science and Pollution Research* **2014**, *21* (1), 740–752. https://doi.org/10.1007/s11356-013-1980-7.
(286) Zaman, S.; Yousuf, A.; Begum, A.; Bari, M. L.; Rabbani, K. S. Evaluation of Adaptive Low Cost Solar Water Pasteurization Device for Providing Safe Potable Water in Rural Households. *Journal of Water and Health* **2019**, *17* (2), 274–286. https://doi.org/10.2166/wh.2019.268.
(287) Abd-Elmaksoud, S.; Naranjo, J. E.; Gerba, C. P. Assessment of a Portable Handheld UV Light Device for the Disinfection of Viruses and Bacteria in Water. *Food and Environmental Virology* **2013**, *5* (2), 87–90. https://doi.org/10.1007/s12560-013-9103-7.
(288) Boehm, A. B.; Soetjipto, C.; Wang, D. Solar Inactivation of Four Salmonella Serovars in Fresh and Marine Waters. *J Water Health* **2012**, *10* (4), 504–510. https://doi.org/10.2166/wh.2012.084.
(289) Brownell, S. A.; Chakrabarti, A. R.; Kaser, F. M.; Connelly, L. G.; Peletz, R. L.; Reygadas, F.; Lang, M. J.; Kammen, D. M.; Nelson, K. L. Assessment of a Low-Cost, Point-of-Use, Ultraviolet Water Disinfection Technology. *Journal of Water and Health* **2008**, *6* (1), 53–65. https://doi.org/10.2166/wh.2007.015.
(290) Butkus, M. A.; Talbot, M.; Labare, M. P. Feasibility of the Silver-UV Process for Drinking Water Disinfection. *Water Res.* **2005**, *39* (20), 4925–4932. https://doi.org/10.1016/j.watres.2005.09.037.
(291) Carratalà, A.; Dionisio Calado, A.; Mattle, M. J.; Meierhofer, R.; Luzi, S.; Kohn, T. Solar Disinfection of Viruses in Polyethylene Terephthalate Bottles. *Appl. Environ. Microbiol.* **2015**, *82* (1), 279–288. https://doi.org/10.1128/AEM.02897-15.
(292) Chatterley, C.; Linden, K. Demonstration and Evaluation of Germicidal UV-LEDs for Point-of-Use Water Disinfection. *J Water Health* **2010**, *8* (3), 479–486. https://doi.org/10.2166/wh.2010.124.
(293) Fisher, M. B.; Keenan, C. R.; Nelson, K. L.; Voelker, B. M. Speeding up Solar Disinfection (SODIS): Effects of Hydrogen Peroxide, Temperature, PH, and Copper plus Ascorbate on the Photoinactivation of E-Coli. *J. Water Health* **2008**, *6* (1), 35–51. https://doi.org/10.2166/wh.2007.005.
(294) Garcia-Gil, A.; Jesus Abeledo-Lameiro, M.; Gomez-Couso, H.; Marugan, J. Kinetic Modeling of the Synergistic Thermal and Spectral Actions on the Inactivation of Cryptosporidium Parvum in Water by Sunlight. *WATER RESEARCH* **2020**, *185*. https://doi.org/10.1016/j.watres.2020.116226.
(295) Garcia-Gil, A.; Martinez, A.; Inmaculada Polo-Lopez, M.; Marugan, J. Kinetic Modeling of the Synergistic Thermal and Spectral Actions on the Inactivation of Viruses in Water by Sunlight. *WATER RESEARCH* **2020**, *183*. https://doi.org/10.1016/j.watres.2020.116074.
(296) Garcia-Gil, A.; Valverde, R.; Garcia-Munoz, R. A.; McGuigan, K. G.; Marugan, J. Solar Water Disinfection in High-Volume Containers: Are Naturally Occurring Substances Attenuating Factors of Radiation? *CHEMICAL ENGINEERING JOURNAL* **2020**, *399*. https://doi.org/10.1016/j.cej.2020.125852.
(297) Gemici, B. T.; Karel, F. B.; Karaer, F.; Koparal, A. S. WATER DISINFECTION WITH ADVANCED METHODS: SUCCESSIVE AND HYBRID APPLICATION OF ANTIBACTERIAL COLUMN WITH SILVER, ULTRASOUND AND UV RADIATION. *Appl. Ecol. Env. Res.* **2018**, *16* (4), 4667–4680. https://doi.org/10.15666/aeer/1604_46674680.

(298) Heaselgrave, W.; Kilvington, S. The Efficacy of Simulated Solar Disinfection (SODIS) against Ascaris, Giardia, Acanthamoeba, Naegleria, Entamoeba and Cryptosporidium. *Acta tropica* **2011**, *119* (2–3), 138–143. https://doi.org/10.1016/j.actatropica.2011.05.004.
(299) Heaselgrave, W.; Patel, N.; Kilvington, S.; Kehoe, S. C.; McGuigan, K. G. Solar Disinfection of Poliovirus and Acanthamoeba Polyphaga Cysts in Water - A Laboratory Study Using Simulated Sunlight. *Letters in Applied Microbiology* **2006**, *43* (2), 125–130. https://doi.org/10.1111/j.1472-765X.2006.01940.x.
(300) Hessling, M.; Gross, A.; Hoenes, K.; Rath, M.; Stangl, F.; Tritschler, H.; Sift, M. Efficient Disinfection of Tap and Surface Water with Single High Power 285 Nm LED and Square Quartz Tube. *Photonics* **2016**, *3* (1), 7. https://doi.org/10.3390/photonics3010007.
(301) Hoenes, K.; Stangl, F.; Sift, M.; Hessling, M. Visible Optical Radiation Generates Bactericidal Effect Applicable for Inactivation of Health Care Associated Germs Demonstrated by Inactivation of E. Coli and B. Subtilis Using 405 Nm and 460 Nm Light Emitting Diodes. In *Novel Biophotonics Techniques and Applications Iii*; Amelink, A., Vitkin, I. A., Eds.; Spie-Int Soc Optical Engineering: Bellingham, 2015; Vol. 9540, p 95400T.
(302) Huffman, D. E.; Slifko, T. R.; Salisbury, K.; Rose, J. B. Inactivation of Bacteria, Virus and Cryptosporidium by a Point-of-Use Device Using Pulsed Broad Spectrum White Light. *Water Res.* **2000**, *34* (9), 2491–2498. https://doi.org/10.1016/S0043-1354(00)00014-2.
(303) Jeco, B. M. F. Y.; Larroder, A. C.; Oguma, K. Technosocial Feasibility Analysis of Solar-Powered UV-LED Water Treatment System in a Remote Island of Guimaras, Philippines. *JOURNAL OF PHOTONICS FOR ENERGY* **2019**, *9* (4). https://doi.org/10.1117/1.JPE.9.043105.
(304) Jenny, R. M.; Jasper, M. N.; Simmons, O. D.; Shatalov, M.; Ducoste, J. J. Heuristic Optimization of a Continuous Flow Point-of-Use UV-LED Disinfection Reactor Using Computational Fluid Dynamics. *Water Res.* **2015**, *83*, 310–318. https://doi.org/10.1016/j.watres.2015.06.031.
(305) Jenny, R. M.; Simmons, O. D.; Shatalov, M.; Ducoste, J. J. Modeling a Continuous Flow Ultraviolet Light Emitting Diode Reactor Using Computational Fluid Dynamics. *Chem. Eng. Sci.* **2014**, *116*, 524–535. https://doi.org/10.1016/j.ces.2014.05.020.
(306) Kehoe, S. C.; Barer, M. R.; Devlin, L. O.; McGuigan, K. G. Batch Process Solar Disinfection Is an Efficient Means of Disinfecting Drinking Water Contaminated with Shigella Dysenteriae Type I. *Lett. Appl. Microbiol.* **2004**, *38* (5), 410–414. https://doi.org/10.1111/j.1472-765X.2004.01515.x.
(307) Li, G.-Q.; Wang, W.-L.; Huo, Z.-Y.; Lu, Y.; Hu, H.-Y. Comparison of UV-LED and Low Pressure UV for Water Disinfection: Photoreactivation and Dark Repair of Escherichia Coli. *Water Res.* **2017**, *126*, 134–143. https://doi.org/10.1016/j.watres.2017.09.030.
(308) Liu, Y.; Dong, S.; Kuhlenschmidt, M. S.; Kuhlenschmidt, T. B.; Drnevich, J.; Nguyen, T. H. Inactivation Mechanisms of Cryptosporidium Parvum Oocysts by Solar Ultraviolet Irradiation. *Environ. Sci.-Wat. Res. Technol.* **2015**, *1* (2), 188–198. https://doi.org/10.1039/c4ew00079j.
(309) Lui, G. Y.; Roser, D.; Corkish, R.; Ashbolt, N. J.; Stuetz, R. Point-of-Use Water Disinfection Using Ultraviolet and Visible Light-Emitting Diodes. *Science of the total environment.* **2016**, 626–635.

(310) Mbonimpa, E. G.; Blatchley, E. R.; Applegate, B.; Harper, W. F. Ultraviolet A and B Wavelength-Dependent Inactivation of Viruses and Bacteria in the Water. *Journal of Water and Health* **2018**, *16* (5), 796–806. https://doi.org/10.2166/wh.2018.071.
(311) Méndez-Hermida, F.; Castro-Hermida, J. A.; Ares-Mazás, E.; Kehoe, S. C.; McGuigan, K. G. Effect of Batch-Process Solar Disinfection on Survival of Cryptosporidium Parvum Oocysts in Drinking Water. *Applied and Environmental Microbiology* **2005**, *71* (3), 1653–1654. https://doi.org/10.1128/AEM.71.3.1653-1654.2005.
(312) Naunovic, Z.; Lim, S.; Blatchley, E. R. Investigation of Microbial Inactivation Efficiency of a UV Disinfection System Employing an Excimer Lamp. *Water Res.* **2008**, *42* (19), 4838–4846. https://doi.org/10.1016/j.watres.2008.09.001.
(313) Nelson, K. Y.; McMartin, D. W.; Yost, C. K.; Runtz, K. J.; Ono, T. Point-of-Use Water Disinfection Using UV Light-Emitting Diodes to Reduce Bacterial Contamination. *Environ Sci Pollut Res Int* **2013**, *20* (8), 5441–5448. https://doi.org/10.1007/s11356-013-1564-6.
(314) Nourmoradi, H.; Nikaeen, M.; Stensvold, C. R.; Mirhendi, H. Ultraviolet Irradiation: An Effective Inactivation Method of Aspergillus Spp. in Water for the Control of Waterborne Nosocomial Aspergillosis. *Water Research* **2012**, *46* (18), 5935–5940. https://doi.org/10.1016/j.watres.2012.08.015.
(315) Nuanualsuwan, S.; Mariam, T.; Himathongkham, S.; Cliver, D. O. Ultraviolet Inactivation of Feline Calicivirus, Human Enteric Viruses and Coliphages. *Photochem. Photobiol.* **2002**, *76* (4), 406–410.
(316) Nyangaresi, P. O.; Qin, Y.; Chen, G.; Zhang, B.; Lu, Y.; Shen, L. Effects of Single and Combined UV-LEDs on Inactivation and Subsequent Reactivation of E. Coli in Water Disinfection. *Water Research* **2018**, *147*, 331–341. https://doi.org/10.1016/j.watres.2018.10.014.
(317) Nyangaresi, P. O.; Qin, Y.; Chen, G.; Zhang, B.; Lu, Y.; Shen, L. Comparison of the Performance of Pulsed and Continuous UVC-LED Irradiation in the Inactivation of Bacteria. *Water Res* **2019**, *157*, 218–227. https://doi.org/10.1016/j.watres.2019.03.080.
(318) Oguma, K.; Kanazawa, K.; Kasuga, I.; Takizawa, S. Effects of UV Irradiation by Light Emitting Diodes on Heterotrophic Bacteria in Tap Water. *Photochem Photobiol* **2018**, *94* (3), 570–576. https://doi.org/10.1111/php.12891.
(319) Pigeot-Rémy, S.; Simonet, F.; Atlan, D.; Lazzaroni, J. C.; Guillard, C. Bactericidal Efficiency and Mode of Action: A Comparative Study of Photochemistry and Photocatalysis. *Water Res.* **2012**, *46* (10), 3208–3218. https://doi.org/10.1016/j.watres.2012.03.019.
(320) Pimenta, A. I.; Guerreiro, D.; Madureira, J.; Margaça, F. M. A.; Cabo Verde, S. Tracking Human Adenovirus Inactivation by Gamma Radiation under Different Environmental Conditions. *Appl. Environ. Microbiol.* **2016**, *82* (17), 5166–5173. https://doi.org/10.1128/AEM.01229-16.
(321) Rattanakul, S.; Oguma, K. Inactivation Kinetics and Efficiencies of UV-LEDs against Pseudomonas Aeruginosa, Legionella Pneumophila, and Surrogate Microorganisms. *Water Research* **2018**, *130*, 31–37. https://doi.org/10.1016/j.watres.2017.11.047.
(322) Schmid, J.; Hoenes, K.; Rath, M.; Vatter, P.; Hessling, M. UV-C Inactivation of Legionella Rubrilucens. *GMD Hyg. Infect. Control* **2017**, *12*, Doc06. https://doi.org/10.3205/dgkh000291.

(323) Silverman, A. I.; Peterson, B. M.; Boehm, A. B.; McNeill, K.; Nelson, K. L. Sunlight Inactivation of Human Viruses and Bacteriophages in Coastal Waters Containing Natural Photosensitizers. *Environmental science & technology* **2013**, *47* (4), 1870–1878. https://doi.org/10.1021/es3036913.
(324) Silverman, A. I.; Nelson, K. L. Modeling the Endogenous Sunlight Inactivation Rates of Laboratory Strain and Wastewater E. Coli and Enterococci Using Biological Weighting Functions. *Environ. Sci. Technol.* **2016**, *50* (22), 12292–12301. https://doi.org/10.1021/acs.est.6b03721.
(325) Simons, R.; Gabbai, U. E.; Moram, M. A. Optical Fluence Modelling for Ultraviolet Light Emitting Diode-Based Water Treatment Systems. *Water Res.* **2014**, *66*, 338–349. https://doi.org/10.1016/j.watres.2014.08.031.
(326) Sisti, M.; Schiavano, G. F.; Santi, M. D.; Brandi, G. Ultraviolet Germicidal Irradiation in Tap Water Contaminated by Aspergillus Spp. *J Prev Med Hyg* **2017**, *58* (4), E315–E319. https://doi.org/10.15167/2421-4248/jpmh2017.58.4.777.
(327) Smith, R. J.; Kehoe, S. C.; McGuigan, K. G.; Barer, M. R. Effects of Simulated Solar Disinfection of Water on Infectivity of Salmonella Typhimurium. *Letters in applied microbiology* **2000**, *31* (4), 284–288. https://doi.org/10.1046/j.1472-765x.2000.00815.x.
(328) Song, K.; Mohseni, M.; Taghipour, F. Mechanisms Investigation on Bacterial Inactivation through Combinations of UV Wavelengths. *Water Res* **2019**, *163*, 114875. https://doi.org/10.1016/j.watres.2019.114875.
(329) Song, K.; Taghipour, F.; Mohseni, M. Microorganisms Inactivation by Wavelength Combinations of Ultraviolet Light-Emitting Diodes (UV-LEDs). *Sci Total Environ* **2019**, *665*, 1103–1110. https://doi.org/10.1016/j.scitotenv.2019.02.041.
(330) Garcia, L. A. T.; Barardi, C. R. M. Performance of a Storage Tank Coupled with UV Light on Enteric Virus Inactivation in Drinking Water. *Water Supply* **2019**, *19* (4), 1103–1109. https://doi.org/10.2166/ws.2018.161.
(331) Ubomba-Jaswa, E.; Boyle, M. a. R.; McGuigan, K. G. Inactivation of Enteropathogenic E. Coli by Solar Disinfection (SODIS) under Simulated Sunlight Conditions. In *Radiation Damage in Biomolecular Systems*; McGuigan, K. G., Ed.; Iop Publishing Ltd: Bristol, 2008; Vol. 101, p 012003.
(332) Woo, H.; Beck, S.; Boczek, L.; Carlson, K.; Brinkman, N.; Linden, K.; Lawal, O.; Hayes, S.; Ryu, H. Efficacy of Inactivation of Human Enteroviruses by Dual-Wavelength Germicidal Ultraviolet (UV-C) Light Emitting Diodes (LEDs). *Water* **2019**, *11* (6), 1131. https://doi.org/10.3390/w11061131.
(333) Wuertele, M. A.; Kolbe, T.; Lipsz, M.; Kuelberg, A.; Weyers, M.; Kneissl, M.; Jekel, M. Application of GaN-Based Ultraviolet-C Light Emitting Diodes - UV LEDs - for Water Disinfection. *Water Res.* **2011**, *45* (3), 1481–1489. https://doi.org/10.1016/j.watres.2010.11.015.
(334) Younis, B. A.; Mahoney, L. E.; Yao, S. Field Evaluation of a Novel UV Water Disinfection System for Use in Underserved Rural Communities. *Water Environ Res* **2019**, *91* (1), 75–82. https://doi.org/10.2175/106143017X15131012188141.
(335) Zou, X.-Y.; Lin, Y.-L.; Xu, B.; Cao, T.-C.; Tang, Y.-L.; Pan, Y.; Gao, Z.-C.; Gao, N.-Y. Enhanced Inactivation of E. Coli by Pulsed UV-LED Irradiation during Water Disinfection. *Science of The Total Environment* **2019**, *650*, 210–215. https://doi.org/10.1016/j.scitotenv.2018.08.367.

(336) Eloidin, O.; Dorea, C. C. Evaluation of Semidecentralized Emergency Drinking Water Treatment. *J Environ Sci Health A Tox Hazard Subst Environ Eng* **2015**, *50* (10), 1040–1045. https://doi.org/10.1080/10934529.2015.1038173.
(337) Marois-Fiset, J.-T.; Carabin, A.; Lavoie, A.; Dorea, C. C. Effects of Temperature and PH on Reduction of Bacteria in a Point-of-Use Drinking Water Treatment Product for Emergency Relief. *Appl. Environ. Microbiol.* **2013**, *79* (6), 2107–2109. https://doi.org/10.1128/AEM.03696-12.
(338) Marois-Fiset, J.-T.; Shaheed, A.; Brown, J.; Dorea, C. C. Laboratory Evaluation of a New Coagulant/Disinfectant Point-of-Use Water Treatment Product for Emergencies. *J. Appl. Microbiol.* **2016**, *121* (3), 892–902. https://doi.org/10.1111/jam.13206.
(339) Souter, P. F.; Cruickshank, G. D.; Tankerville, M. Z.; Keswick, B. H.; Ellis, B. D.; Langworthy, D. E.; Metz, K. A.; Appleby, M. R.; Hamilton, N.; Jones, A. L.; Perry, J. D. Evaluation of a New Water Treatment for Point-of-Use Household Applications to Remove Microorganisms and Arsenic from Drinking Water. *J Water Health* **2003**, *1* (2), 73–84.
(340) Légaré-Julien, F.; Lemay, O.; Vallée-Godbout, U.; Bouchard, C.; Dorea, C. Laboratory Efficacy and Disinfection By-Product Formation of a Coagulant/Disinfectant Tablet for Point-of-Use Water Treatment. *Water* **2018**, *10* (11), 1567. https://doi.org/10.3390/w10111567.
(341) Kfir, R.; Bateman, B. W.; Pitout, B. A.; Coubrough, P. Disinfection of Polluted Water by Chlorine-Flocculant Tablet. *Water Science and Technology* **1989**, *21* (3), 207–213. https://doi.org/10.2166/wst.1989.0101.
(342) Rodda, N.; Bateman, B.; Kfir, R. Removal of Salmonella Typhi, Shigella Dysenteriae, Vibrio Cholerae and Rotavirus from Water Using a Water Treatment Tablet. *Water Science and Technology* **1993**, *27* (3–4), 347–350. https://doi.org/10.2166/wst.1993.0373.
